# Construction of Unusual Indole-Based Heterocycles from Tetrahydro-1*H*-pyridazino[3,4-*b*]indoles

**DOI:** 10.3390/molecules25184124

**Published:** 2020-09-09

**Authors:** Cecilia Ciccolini, Lucia De Crescentini, Fabio Mantellini, Giacomo Mari, Stefania Santeusanio, Gianfranco Favi

**Affiliations:** Department of Biomolecular Sciences, Section of Chemistry and Pharmaceutical Technologies, University of Urbino “Carlo Bo”, Via I Maggetti 24, 61029 Urbino, Italy; c.ciccolini@campus.uniurb.it (C.C.); lucia.decrescentini@uniurb.it (L.D.C.); fabio.mantellini@uniurb.it (F.M.); giacomo.mari@uniurb.it (G.M.); stefania.santeusanio@uniurb.it (S.S.)

**Keywords:** indole-based heterocycles, C2-C3 indole oxidation, aromatization, ring-opening/ring-closing, umpolung

## Abstract

Herein, we report the successful syntheses of scarcely represented indole-based heterocycles which have a structural connection with biologically active natural-like molecules. The selective oxidation of indoline nucleus to indole, hydrolysis of ester and carbamoyl residues followed by decarboxylation with concomitant aromatization of the pyridazine ring starting from tetrahydro-1*H*-pyridazino[3,4-*b*]indole derivatives lead to fused indole-pyridazine compounds. On the other hand, non-fused indole-pyrazol-5-one scaffolds are easily prepared by subjecting the same C2,C3-fused indoline tetrahydropyridazines to treatment with trifluoroacetic acid (TFA). These methods feature mild conditions, easy operation, high yields in most cases avoiding the chromatographic purification, and broad substrate scope. Interestingly, the formation of indole linked pyrazol-5-one system serves as a good example of the application of the umpolung strategy in the synthesis of C3-alkylated indoles.

## 1. Introduction

**Figure 1:**
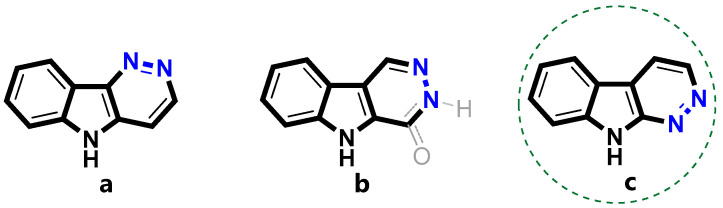
Different isomeric fused indole-pyridazine systems.

Given the intriguing structures and the medicinal importance of polycyclic indole-based molecules [1,2,3,4,5,6,7], we envisioned that the amalgamation of the indole moiety [8,9,10,11,12,13] with the pyridazine ring [14,15,16,17,18,19] that potentially generates different isomeric scaffolds (**a**–**c**) would lead to entities endowed with either amplified or new biological activities (Figure 1). A large number of reports dedicated to the synthesis of these appealing frameworks and studies on their pharmacologic activities appeared in the literature in the past few decades [20,21,22,23,24,25,26,27,28,29]. Among the heterocyclic architectures **a**–**c**, the tricyclic fused indole-pyridazine system **c** which can be considered to be aza-analogous (or bioisoster) of β-carboline, the unique tricyclic pyrido[3,4-*b*]indole core amenable to an important family of bioactive natural products widely distributed in nature [30], has attracted our attention.

Although many of the above cited examples deal with 5*H*-pyridazino[4,3-*b*]indoles (**a**) [20,21] and 5*H*-pyridazino[4,5-*b*]indoles or 3*H*-pyridazino[4,5-*b*]indol-4(5*H*)-ones (**b**) [22,23,24,25,26], the chemistry of 9*H*-pyridazine[3,4-*b*]indoles (**c**) [27,28,29] is much less known.

Specifically, in 1964, Kobayashi and Furukawa presented the synthesis of various 3-phenyl-9*H*-pyridazine[3,4-*b*]indoles by heating 3-phenacyl-oxindoles and hydrazine hydrate in acetic acid solution [27]. In 1992, Shimoji and co-workers synthesized a series of methyl 9*H*-pyridazino[3,4-*b*]indole-3-carboxylates and related compounds using a Diels-Alder reaction of 3-(1*H*-indol-3-yl)-2-propenoates with dibenzyl dicarboxylate [28]. Several of these compounds were found to have high affinity for the benzodiazepine receptor. Recently, the design, synthesis, biological evaluation, and molecular modeling studies of several 3-aryl-9-acetyl-pyridazino[3,4-*b*]indoles were reported by Nepali et al. [29].

Following our sustained efforts toward the construction of novel azaheterocycles, especially around privileged structures [31], we have recently disclosed a zinc-catalyzed synthesis of polycylic fused indoline scaffolds through a substrate-guided reactivity switch [32]. With this as a background, we herein describe a successful procedure providing the fused indole-pyridazine scaffold of type **c** (Figure 1)through oxidation and hydrolysis processes from previously synthesized tetrahydro-1*H*-pyridazino[3,4-*b*]indole compounds (**1**). Surprised to observe a ring-opening/ring-closing pathway during the C2-C3 indole oxidation step, a facile and robust access to unusual non-fused derivative indole-pyrazol-5-ones **4** starting from the same cycloadducts (**1**) has been also achieved in excellent yields.

## 2. Results and Discussion

In a preliminary experiment, the conversion of tetrahydro-1*H*-pyridazino[3,4-*b*]indole **1b** to fully aromatic 9*H*-pyridazino[3,4-*b*]indole **3b** was attempted by a two-step process consisting of decarboxylation of the ester motif with removal of carbamoyl residue followed by oxidation of pyridazine ring under basic conditions. However, although removal of the ester with alcoholic KOH efficiently took place affording a mixture of CH/NH tautomers, unexpectedly pyridazine ring aromatization did not occur. With this failure in hand, we first turned our attention to the crucial C2-C3 indole oxidation step. A variety of oxidizing agents (e.g., DDQ [33], BQ, NBS/TBPB, MnO_2_ [34,35,36], Na_2_Cr_2_O_7_, I_2_, PCC [37], Pd/C [38], CAN [39] and CuCl_2_∙2H_2_O [40]) was investigated (Table 1). Common reagents such as BQ, Na_2_Cr_2_O_7_, PCC, Pd/C, I_2_, and NBS in combination with TBPB, although frequently used for oxidation of the indoline ring, proved unsuccessful. With stoichiometric DDQ in dioxane, only trace of the aromatic indole-pyridazine **2b** was formed (Table 1, entry 1). The dehydrogenations reaction yield could be further improved by using toluene and CH_2_Cl_2_ as solvents (Table 1, entries 2 and 3). Manganese dioxide was reported in the literature to be suitable for the oxidation of indolines to indoles [34,35,36]. To our surprise, when compound **1b** was subjected to treatment with MnO_2_ in benzene at 70 °C, the sole demethylated indole derivative **2b’** was isolated in 55% yield [41] (see Supplementary Materials). Reducing the amount of MnO_2_ displayed an incomplete reaction, decreasing the reaction yield (40% yield) of the obtained product (Table 1, entries 6 and 7).

On the other hand, the use of oxidizing systems such as ceric ammonium nitrate (CAN) and catalytic copper (II) chloride dihydrate in DMSO did not afford the expected product **2b**, providing instead the non-fused indole-pyrazole **4c** (Table 1, entries 12 and 13).

**Scheme 1:**
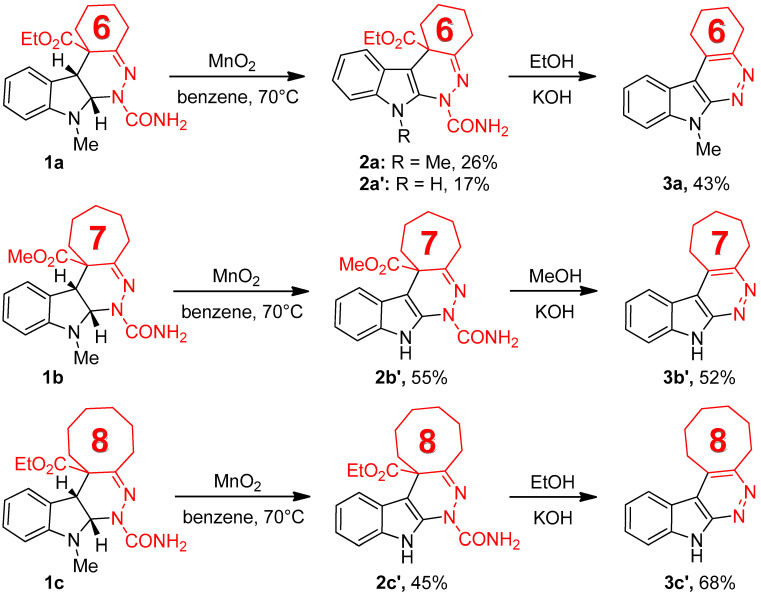
Two-step synthesis of cyclo-fused pyridazino[3,4-*b*]indoles **3a**, **3b’**, **3c’**.

With these results in hand, also tetracylic compounds incorporating 6 and 8 membered fused ring such as **1a** and **1c** were subjected to optimal oxidation conditions to obtain the relative compounds **2a**/**2a’** and **2c’** respectively (Scheme 1). Intrigued by the possibility of obtaining fully aromatic derivatives, the treatment of representative 6, 7, and 8 membered tetrahydro-1*H*-pyridazino[3,4-*b*]indole **2a**, **2b’**, **2c’** with alcoholic KOH was also carried out. To our delight, the formation of cyclo-fused pyridazino[3,4-*b*]indoles **3a**, **3b’** and **3c’** (via hydrolysis of ester and carbamoyl residues followed by decarboxylation with concomitant aromatization of the pyridazine ring) was registered with success (43–68% yields) (Scheme 1).

**Scheme 2:**
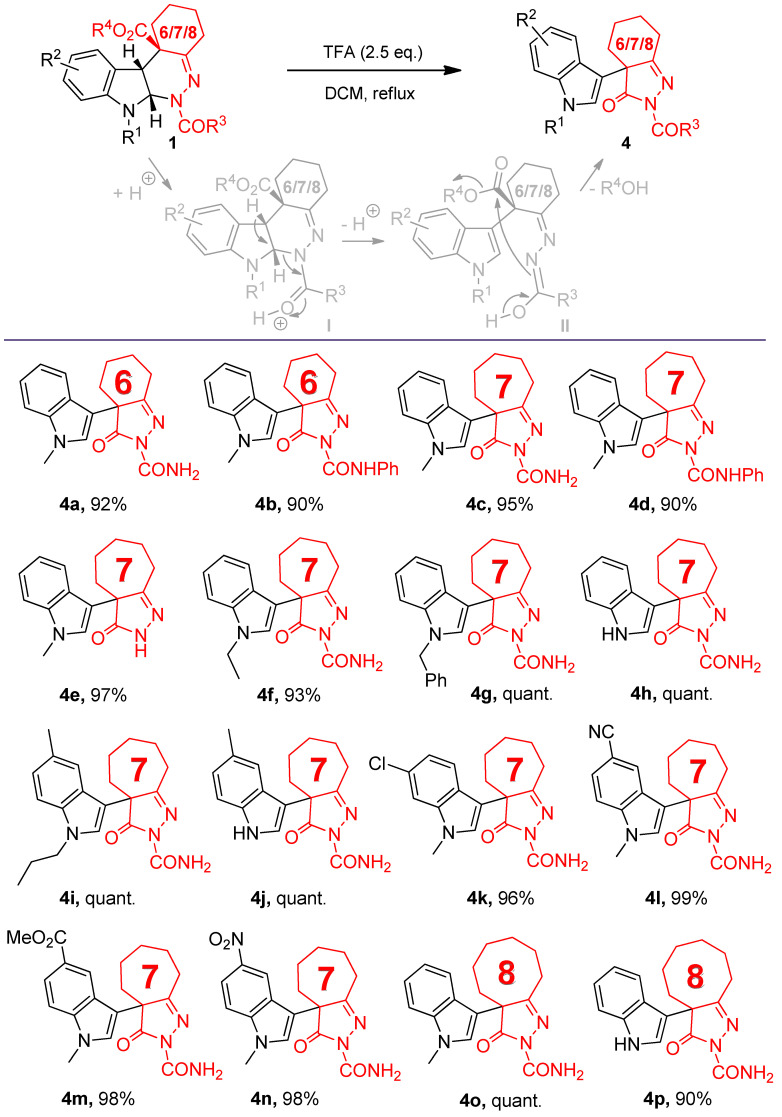
Substrate scope for the synthesis of indole linked pyrazol-5-ones **4**.

Encouraged by the unexpected formation of non-fused indole-pyrazol-5-one **4c** during the investigation for indoline oxidation to indole, we next focused our attention to exploring this transformation. We identified that the use of trifluoroacetic acid (TFA) as common Brønsted acid induced the ring-opening of the cycloadduct **1b** to give non-fused *N*-polyheterocyclic compound **4c** in excellent yield (95%). It was found that all the tetracyclic compounds embedding 6, 7 and 8 membered-ring well tolerated the acidic environment (Scheme 2). Changing the substituents (alkyl, benzyl) on the *N*-indole ring, the reaction proceeded satisfactorily (**4f**, **4g**, and **4i**). Also, the free *N*H-indole was proven to be a good candidate for this reaction furnishing the relative products **4h**, **4j**, and **4p** in very excellent yields. As expected, conversion to *N*H-pyrazol-5-one **4e** obtained by removal of the *t*-butoxycarbonyl (Boc) protective group was observed under such conditions. The wide functional groups tolerance of this procedure was validated from the introduction of electron-donor (**4i**, and **4j**) or electron-attractor (**4k**, **4l**, **4m**, **4n**) substituents on the aromatic component which led to the corresponding indole linked pyrazol-5-one systems in almost quantitative yields. Interesting to note that no purification of the obtained products by flash chromatography column was necessary because of their precipitation from the reaction medium. A plausible mechanism could involve TFA-induced ring-opening of the tetrahydropyridazine, followed by sequential intramolecular nucleophilic acyl substitution (ring-closing) to form the final product accompanied by elimination of an alcohol molecule. This pathway reflects the well-established tendency of polycyclic fused indoline structures without 3-substituents to rearomatization [42]. Also, the formation of NH tautomeric form of **II** previously described by our group [32] supports this.

**Scheme 3:**
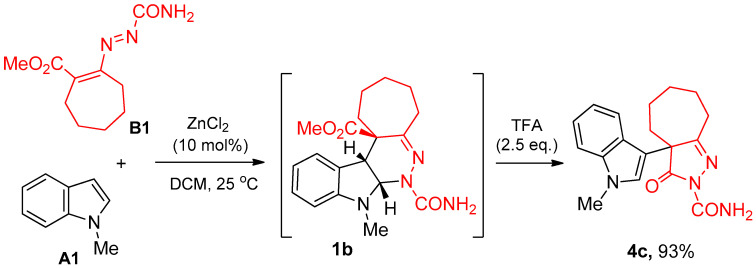
One-pot synthesis of **4c** from *N*-methyl indole **A1** and cyclic azoalkene **B1**.

To illustrate the synthetic potential of this transformation, a one-pot reaction involving the in situ formation of [4+2] cycloadduct **1b** from *N*-methyl indole **A1** and cyclic azoalkene **B1** [32] was also performed. The reaction proceeded very well, and desired product **4c** was obtained in 93% overall yield (Scheme 3).

To date, only one example of the synthesis of compound of type **4** was documented in the literature [43]. In their work, Shi and co-workers realized an umpolung of C3 indole reactivity, using 2-indolylmethanols (**C**) as an electrophile and pyrazol-5-ones (**D**) as a nucleophile to obtain, under the cooperative catalysis of Pd(0) and a chiral phosphoric acid, the strategic C-C bond formation.

**Scheme 4:**
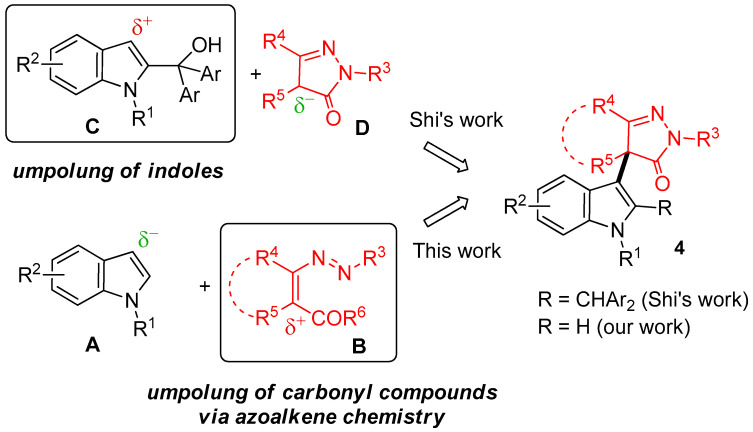
Different synthetic approaches to indole linked pyrazol-5-one derivatives **4**.

Differently from Shi’s work, in our case the same C-C bond formation is realized exploiting the reversal of polarity of the azoalkene (**B**) system (“umpoled” of carbonyl compounds) [44,45,46,47]. Taking advantage of the intrinsic reactivity of the pertinent substrates, a new and complementary synthetic approach toward these unusual *N*-polyheterocyclic structures can be successfully achieved (Scheme 4). We believe that the present method is of operational interest in terms of forming otherwise challenging-to-prepare C−C bonds.

## 3. Materials and Methods

### 3.1. General

All the commercially available reagents and solvents were used without further purification. Tetrahydro-1*H*-pyridazino[3,4-*b*]indoles **1** were prepared via a formal [4+2] cycloaddition reactions of 2,3-unsubstituted indoles with cyclic azoalkenes by known procedures [32]. Chromatographic purification of compounds was carried out on silica gel (60–200 μm). Thin-layer chromatography (TLC) analysis was performed on pre-loaded (0.25 mm) glass supported silica gel plates (Silica gel 60, F254, Merck; Darmstadt, Germany); compounds were visualized by exposure to UV light. Melting points (m.p.) were determined in open capillary tubes and are uncorrected.

All ^1^H-NMR and ^13^C-NMR spectra were recorded at 400 and 100 MHz, respectively at 25 °C on a Bruker Ultrashield 400 spectrometer (Bruker, Billerica, MA, USA). Proton and carbon spectra were referenced internally to residual solvent signals as follows: δ = 2.50 ppm for proton (middle peak) and δ = 39.50 ppm for carbon (middle peak) in DMSO-*d*_6_ and δ = 7.27 ppm for proton and δ = 77.00 ppm for carbon (middle peak) in CDCl_3_. The following abbreviations are used to describe peak patterns where appropriate: s = singlet, d = doublet, t = triplet q = quartet, m = multiplet and br = broad signal. All coupling constants (*J*) are given in Hz. FT-IR spectra were measured as Nujol mulls using a Nicolet Impact 400 (Thermo Scientific, Madison, WI, USA). Low-resolution mass spectra (LRMS) was performed on a Waters Micromass Q-ToF instrument (Waters, Milford, MA, USA) using an ESI source. Elemental analyses were within ± 0.4 of the theoretical values (C, H, N).

### 3.2. Two-Step Procedure for the Synthesis of ***3***

#### 3.2.1. Procedure for the Oxidation of Indolines 1 to Indoles 2

To a stirred solution of compound **1** (0.5 mmol) in benzene (1 mL), MnO_2_ (25 eq) was added and the reaction was subsequently warmed up to 70 °C (oil bath). The reaction mixture was kept at this temperature until the reagent had been completely consumed as monitored by TLC (20 h). The crude mixture was then filtered and purified by column chromatography on silica gel using hexane-ethyl acetate as the eluent to afford product **2** (or **2’**).

*Ethyl 6-carbamoyl-7-methyl-2,3,4,6,7,11c-hexahydro-1H-indolo[2,3-c]cinnoline-11c-carboxylate* (**2a**). Yield 26% (46.1 mg) as a whitish solid; m.p. 179–181 °C; ^1^H-NMR, 400 MHz, CDCl_3_): δ 1.18 (t, *J* = 7.2 Hz, 3 H), 1.49–1.89 (m, 4 H), 1.96–2.01 (m, 1 H), 2.47 (dt, *J*_1_ = 14.4 Hz, *J*_2_ = 5.2 Hz, 1 H), 2.69–2.75 (m, 1 H), 3.45–3.52 (m, 1 H), 3.72 (s, 3 H), 4.05–4.20 (m, 2 H), 5.14 (br, 1 H), 6.58 (br, 1 H), 7.09 (dt, *J*_1_ = 8.0 Hz, *J*_2_ = 1.2 Hz, 1 H), 7.17 (dt, *J*_1_ = 8.0 Hz, *J*_2_ = 1.2 Hz, 1 H), 7.29 (d, *J* = 8.0 Hz, 1 H), 7.66 (d, *J* = 8.0 Hz, 1 H) ppm; ^13^C-NMR (100 MHz, CDCl_3_): δ 14.1, 22.9, 26.0, 33.4, 33.6, 35.4, 50.8, 61.8, 96.5, 109.9, 119.8, 120.1, 121.3, 123.6, 131.3, 136.8, 154.4, 156.2, 170.5 ppm; IR (Nujol, cm^−1^): ν_max_ 3491, 3363, 1734, 1703; MS (ESI): *m*/*z* 355 [M + H]^+^; anal. calcd. for C_19_H_22_N_4_O_3_ (354.40): C 64.39, H 6.26, N 15.81; found: C 64.25, H 6.32, N 15.94.

*Ethyl 6-carbamoyl-2,3,4,6,7,11c-hexahydro-1H-indolo[2,3-c]cinnoline-11c-carboxylate* (**2a’**). Yield 17% (28.9 mg) as a whitish solid; m.p. 159–161 °C; ^1^H-NMR, 400 MHz, CDCl_3_): δ 1.22 (t, *J* = 7.2 Hz, 3 H), 1.51–1.89 (m, 4 H), 1.95−2.02 (m, 1 H), 2.48 (dt, *J*_1_ = 13.2 Hz, *J*_2_ = 4.8 Hz, 1 H), 2.62 (d, *J* = 13.2 Hz, 1 H), 3.34 (d, *J* = 13.2 Hz, 1 H), 4.09–4.29 (m, 2 H), 5.26 (br, 1 H), 6.79 (br, 1 H), 7.07–7.14 (m, 2 H), 7.32 (d, *J* = 7.2 Hz, 1 H), 7.63 (d, *J* = 7.2 Hz, 1 H), 10.13 (s, 1 H) ppm; ^13^C-NMR (100 MHz, CDCl_3_): δ 14.3, 23.3, 26.8, 34.3, 36.4, 50.4, 61.8, 91.9, 111.1, 119.2, 120.2, 121.1, 123.8, 129.6, 132.7, 151.8, 155.1, 170.6 ppm; IR (Nujol, cm^−1^): ν_max_ 3455, 3425, 3297, 1720, 1686; MS (ESI): *m/z* 341 [M + H]^+^; C_18_H_20_N_4_O_3_ (340.37): C 63.52, H 5.92, N 16.46; found: C 63.69, H 5.84, N 16.34.

*Methyl 7-carbamoyl-8-methyl-1,2,3,4,5,7,8,12c-octahydrocyclohepta[5,6]pyridazino[3,4-b]indole-12c-carboxylate* (**2b**). Yield 27% (47.8 mg) as a whitish oil; ^1^H-NMR (400 MHz, DMSO-*d*_6_, 25 °C): δ 1.00−1.10 (m, 1 H), 1.17–1.29 (m, 1 H), 1.40−1.49 (m, 1 H), 1.58–1.67 (m, 1 H), 1.75–1.82 (m, 1 H), 1.89−1.97 (m, 1 H), 2.17–2.24 (m, 1 H), 2.55−2.63 (m, 1 H), 2.77–2.83 (m, 1 H), 3.06–3.13 (m, 1 H), 3.53 (s, 3 H), 3.61 (s, 3 H), 6.99 (br, 2 H), 7.05 (dt, *J*_1_ = 8.0 Hz, *J*_2_ = 0.8 Hz, 1 H), 7.13 (dt, *J*_1_ = 8.0 Hz, *J*_2_ = 0.8 Hz, 1 H), 7.41 (d, *J* = 8.0 Hz, 1 H), 7.56 (d, *J* = 7.2 Hz, 1 H); ^13^C-NMR (100 MHz, DMSO-*d*_6_, 25 °C): δ 23.6, 28.9, 29.3, 32.3, 32.6, 33.4, 52.5, 52.6, 94.7, 109.9, 118.4, 119.9, 120.6, 122.7, 133.8, 136.3, 153.1, 158.9, 171.7; IR (Nujol, cm^−1^): ν_max_ 3488, 3375, 1729, 1708; MS (ESI) *m/z* 355 [M + H]^+^; anal. calcd. for C_19_H_22_N_4_O_3_ (354.40): C 64.39, H 6.26, N 15.18; found: C 64.26, H 6.34, N 15.29.

*Methyl 7-carbamoyl-1,2,3,4,5,7,8,12c-octahydrocyclohepta[5,6]pyridazino[3,4-b]indole-12c-carboxylate* (**2b’**). Yield 55% (93.6 mg) as a white solid; m.p. 181–183 °C; ^1^H-NMR, 400 MHz, DMSO-*d*_6_): δ 1.10–1.23 (m, 1 H), 1.38–1.47 (m, 2 H), 1.65–1.74 (m, 2 H), 1.89–1.93 (m, 1 H), 2.19–2.35 (m, 2 H), 2.65–2.71 (m, 1 H), 2.84–2.90 (m, 1 H), 3.57 (s, 3 H), 6.93−7.01 (m, 2 H), 7.26 (s, 2 H), 7.44–7.50 (m, 2 H), 11.21 (s, 1 H) ppm; ^13^C-NMR (100 MHz, DMSO-*d*_6_): δ 22.9, 29.2, 29.6, 33.7, 33.8, 51.5, 52.4, 89.9, 112.2, 117.7, 119.5, 120.1, 123.1, 131.0, 133.4, 150.9, 154.1, 171.9 ppm; IR (Nujol, cm^−1^): ν_max_ 3491, 3430, 3297, 1734, 1707; MS (ESI): *m/z* 341 [M + H]^+^; anal. calcd. for C_18_H_20_N_4_O_3_ (340.38): C 63.52, H 5.92, N 16.46; found: C 63.39, H 6.01, N 16.57.

*Ethyl 8-carbamoyl-2,3,4,5,6,8,9,13c-octahydro-1H-cycloocta[5,6]pyridazino[3,4-b]indole-13c-carboxylate* (**2c’**). Yield 45% (82.9 mg) as a white solid; m.p. 192–194 °C; ^1^H-NMR, 400 MHz, CDCl_3_): δ 1.15 (t, *J* = 7.2 Hz, 3 H), 1.18–1.24 (m, 1 H), 1.35–1.55 (m, 4 H), 1.61−1.81 (m, 2 H), 1.94–2.01 (m, 1 H), 2.33−2.48 (m, 2 H), 2.65–2.71 (m, 1 H), 2.87−2.93 (m, 1 H), 4.01–4.20 (m, 2 H), 5.07 (br, 1 H), 6.76 (br, 1 H), 7.06–7.14 (m, 2 H), 7.32 (d, *J* = 8.0 Hz, 1 H), 7.60 (d, *J* = 8.0 Hz, 1 H); 10.13 (s, 1 H) ppm; ^13^C-NMR (100 MHz, CDCl_3_): δ 14.2, 23.7, 24.2, 24.9, 28.3, 31.3, 32.9, 52.1, 61.7, 89.6, 111.2, 118.9, 120.3, 121.2, 124.3, 131.1, 132.8, 154.6, 155.0, 171.5 ppm; IR (Nujol, cm^−1^): ν_max_ 3410, 3276, 3220, 1724, 1698; MS (ESI): *m/z* 369 [M + H]^+^; anal. calcd. for C_20_H_24_N_4_O_3_ (368.43): C 65.20, H 6.57, N 15.21; found: C 65.06, H 6.63, N 15.30.

#### 3.2.2. Procedure for the Preparation of Fused Indole-Pyridazine 3 from **2**

To a stirred solution of KOH (10 eq.) in alcohol (3 mL), the compound **2** (or **2’**) (0.2 mmol) was added and the mixture was refluxed until the disappearance of the reagent (TLC check, 7 h). The crude mixture was then filtered and purified by column chromatography on silica gel to afford product **3**(or **3’**).

*1,2,3,4,5,8-Hexahydrocyclohepta[5,6]pyridazino[3,4-b]indole* (**3a**). Yield 43% (20.5 mg) as a yellowish solid; m.p. 167–169 °C; ^1^H-NMR (400 MHz, DMSO-*d*_6_): δ 1.92–1.96 (m, 3 H), 2.07–2.09 (m, 3 H), 2.13–2.20 (m, 2 H), 3.99 (s, 3 H), 7.31–7.36 (m, 1 H), 7.68−7.74 (m, 2 H), 8.20 (d, *J* = 8.0 Hz, 1 H) ppm; ^13^C-NMR (100 MHz, DMSO-*d*_6_): δ 21.6, 22.5, 25.7, 27.9, 29.8, 109.9, 116.4, 118.3, 120.4, 125.1, 129.4, 130.5, 141.5, 151.1, 152.2 ppm; IR (Nujol, cm^−1^): ν_max_ no significant signals were detected; MS (ESI): *m/z* 238 [M + H]^+^; anal. calcd. for C_15_H_15_N_3_ (237.39): C 75.92, H 6.37, N 17.71; found: C 76.03, H 6.45, N 17.59.

*1,2,3,4,5,8-Hexahydrocyclohepta[5,6]pyridazino[3,4-b]indole* (**3b’**). Yield 52% (24.7 mg) as a yellowish solid; m.p. 180–182 °C; ^1^H-NMR (400 MHz, DMSO-*d*_6_): δ 1.66–1.71 (m, 2 H), 1.75–1.81 (m, 2 H), 1.88–1.94 (m, 2 H), 3.30–3.34 (m, 2 H), 3.39–3.43 (m, 2 H), 7.26 (t, *J* = 8.0 Hz, 1 H), 7.53 (d, *J* = 8.0 Hz, 1 H), 7.59 (t, *J* = 8.0 Hz, 1 H), 8.35 (d, *J* = 8.0 Hz, 1 H), 12.10 (s, 1 H) ppm; ^13^C-NMR (100 MHz, DMSO-*d*_6_): δ 26.8, 26.9, 30.2, 32.2, 34.9, 113.1, 115.3, 119.1, 121.2, 125.0, 130.7, 138.7, 142.4, 153.3, 156.4 ppm; IR (Nujol, cm^−1^): ν_max_ no significant signals were detected; MS (ESI): *m/z* 238 [M + H]^+^; anal. calcd. for C_15_H_15_N_3_ (237.29): C 75.92, H 6.37, N 17.71; found: C 76.07, H 6.44, N 17.62.

*2,3,4,5,6,9-Hexahydro-1H-cycloocta[5,6]pyridazino[3,4-b]indole* (**3c’**). Yield 68% (34.2 mg) as a yellowish solid; m.p. 195–197 °C; ^1^H-NMR (400 MHz, DMSO-*d*_6_): δ 1.18–1.31 (m, 2 H), 1.38–1.44 (m, 2 H), 1.70–1.81 (m, 2 H), 1.83–1.95 (m, 2 H), 3.24–3.29 (m, 2 H), 3.31–3.41 (m, 2 H), 7.29 (t, *J* = 8.0 Hz, 1 H), 7.55 (d, *J* = 8.0 Hz, 1 H), 7.61 (t, *J* = 8.0 Hz, 1 H), 8.21 (d, *J* = 8.0 Hz, 1 H), 12.12 (s, 1 H) ppm; ^13^C-NMR (100 MHz, DMSO-*d*_6_): δ 25.5, 25.7, 26.5, 28.3, 31.4, 31.7, 111.8, 116.3, 118.5, 120.2, 124.6, 129.2, 133.5, 140.9, 153.8, 154.9 ppm; IR (Nujol, cm^−1^): ν_max_ no significant signals were detected; MS (ESI): *m/z* 252 [M + H]^+^; anal. calcd. for C_16_H_17_N_3_ (251.32): C 76.46, H 6.82, N 16.72; found: C 76.32, H 6.89, N 16.60.

### 3.3. General Procedure for the Synthesis of Indole Linked Pyrazol-5-ones ***4***

To a stirred solution of compound **1** (0.2 mmol) in methylene chloride (2 mL), TFA (2.5 eq) was added at room temperature. The reaction was refluxed until TLC indicated the disappearance of the reagent (TLC check, 2 h). Removed the solvent under reduced pressure, the crude mixture was diluted with water and extracted with EtOAc (2 × 10 mL). The organic phase was dried with Na_2_SO_4_ and solvent was evaporated in vacuo. The compound **4** was collected by precipitation as a white solid, filtered, and washed with ethyl ether.

*3a-(1-methyl-1H-indol-3-yl)-3-oxo-3,3a,4,5,6,7-hexahydro-2H-indazole-2-carboxamide* (**4a**). Yield 92% (57.2 mg) as a white solid; m.p. 192–194 °C; ^1^H-NMR (400 MHz, DMSO-*d*_6_): δ 1.39–1.52 (m, 1 H), 1.58–1.78 (m, 3 H), 1.92–1.99 (m, 1 H), 2.21 (dt, *J*_1_ = 13.2 Hz, *J*_2_ = 5.6 Hz, 1 H), 2.59 (d, *J* = 12.8 Hz, 1 H), 2.82 (d, *J* = 12.8 Hz, 1 H), 3.80 (s, 3 H), 7.02 (t, *J* = 8.0 Hz, 1 H), 7.18 (t, *J* = 8.0 Hz, 1 H), 7.24 (d, *J* = 8.0 Hz, 1 H), 7.26 (br, 1 H), 7.45 (d, *J* = 8.0 Hz, 1 H), 7.53 (br, 1 H), 7.57 (s, 1 H) ppm; ^13^C-NMR (100 MHz, DMSO-*d*_6_): δ 20.9, 27.0, 28.0, 32.6, 33.5, 54.3, 105.7, 110.3, 117.8, 119.5, 121.7, 125.1, 129.1, 136.9, 149.7, 166.5, 176,8 ppm; IR (Nujol, cm^−1^): ν_max_ 3399, 3251, 1726, 1702; MS (ESI): *m/z* 311 [M + H]^+^; anal. calcd. for C_17_H_18_N_4_O_2_ (310.35): C 65.79, H 5.85, N 18.05; found: C 65.64, H 5.92, N 17.92.

*3a-(1-methyl-1H-indol-3-yl)-3-oxo-N-phenyl-3,3a,4,5,6,7-hexahydro-2H-indazole-2-carboxamide* (**4b**). Yield 90% (69.6 mg) as a white solid; m.p. 220–222 °C; ^1^H-NMR (400 MHz, DMSO-*d*_6_): δ 1.44–1.57 (m, 1 H), 1.59–1.75 (m, 2 H), 1.86 (dt, *J*_1_ = 13.2 Hz, *J*_2_ = 5.6 Hz, 1 H), 1.96–2.01 (m, 1 H), 2.26 (dt, *J*_1_ = 13.2 Hz, *J*_2_ = 5.6 Hz, 1 H), 2.68 (d, *J* = 12.8 Hz, 1 H), 2.85 (d, *J* = 12.8 Hz, 1 H), 3.81 (s, 3 H), 7.04 (dt, *J*_1_ = 8.0 Hz, *J*_2_ = 0.8 Hz, 1 H), 7.09 (dt, *J*_1_ = 8.0 Hz, *J*_2_ = 0.8 Hz, 1 H), 7.18 (dt, *J*_1_ = 8.0 Hz, *J*_2_ = 0.8 Hz, 1 H), 7.27–7.38 (m, 3 H), 7.46 (d, *J* = 8.0 Hz, 1 H), 7.57 (d, *J* = 8.0 Hz, 2 H), 7.62 (s, 1 H), 9.82 (s, 1 H) ppm; ^13^C-NMR (100 MHz, DMSO-*d*_6_): δ 20.9, 27.1, 28.0, 32.6, 33.5, 54.5, 105.6, 110.4, 117.9, 119.7, 120.1, 121.7, 123.9, 125.2, 128.9, 129.2, 137.0, 137.4, 147.0, 167.2, 176.6 ppm; IR (Nujol, cm^−1^): ν_max_ 3236, 1744, 1709; MS (ESI): *m/z* 387 [M + H]^+^; anal. calcd. for C_23_H_22_N_4_O_2_ (386.45): C 71.48, H 5.74, N 14.50; found: C 71.35, H 5.83, N 14.62.

*8a-(1-Methyl-1H-indol-3-yl)-1-oxo-4,5,6,7,8,8a-hexahydrocyclohepta[c]pyrazole-2(1H)-carboxamide* (**4c**). Yield 95% (61.6 mg) as a white solid; m.p. 131–133 °C; ^1^H-NMR (400 MHz, DMSO-*d*_6_): δ 1.28–1.35 (m, 1 H), 1.48–1.69 (m, 4 H), 1.76–1.85 (m, 1 H), 2.15–2.23 (m, 1 H), 2.37–2.46 (m, 2 H), 2.69–2.75 (m, 1 H), 3.78 (s, 3 H), 7.02 (dt, *J*_1_ = 8.0 Hz, *J*_2_ = 0.8 Hz, 1 H), 7.17 (dt, *J*_1_ = 8.0 Hz, *J*_2_ = 0.8 Hz, 1 H), 7.24 (br, 1 H), 7.31 (d, *J* = 8.0 Hz, 1 H), 7.44 (d, *J* = 8.0 Hz, 1 H), 7.46 (s, 1 H), 7.53 (br, 1 H) ppm; ^13^C-NMR (100 MHz, DMSO-*d*_6_): δ 24.7, 25.9, 28.5, 29.3, 32.6, 32.8, 58.5, 106.9, 110.3, 118.4, 119.5, 121.7, 124.8, 128.5, 136.9, 149.4, 166.7, 176.6 ppm; IR (Nujol, cm^−1^): ν_max_ 3425, 3267, 1729, 1683; MS (ESI): *m/z* 325 (M + H)^+^; anal. calcd. for C_18_H_20_N_4_O_2_ (324.38): C 66.65, H 6.21, N 17.27; found: C 66.51, H 6.28, N 17.39; found: C 66.64, H 6.17, N 17.27.

*3a-(1-methyl-1H-indol-3-yl)-3-oxo-N-phenyl-3a,4,5,6,7,8-hexahydrocyclohepta[c]pyrazole-2(3H)-carboxamide* (**4d**). Yield 90% (72.1 mg) as a white solid; m.p. 166-168 °C; ^1^H-NMR (400 MHz, DMSO-*d*_6_): δ 1.35–1.45 (m, 1 H), 1.50–1.71 (m, 4 H), 1.78–1.87 (m, 1 H), 2.23–2.31 (m, 1 H), 2.43–2.55 (m, 2 H), 2.77–2.85 (m, 1 H), 3.79 (s, 3 H), 7.05 (t, *J* = 7.2 Hz, 1 H), 7.09 (t, *J* = 7.2 Hz, 1 H), 7.18 (t, *J* = 7.6 Hz, 1 H), 7.33 (t, *J* = 8.0 Hz, 2 H), 7.41 (d, *J* = 8.0 Hz, 1 H), 7.45 (d, *J* = 8.0 Hz, 1 H), 7.50 (s, 1 H), 7.58 (d, *J* = 8.0 Hz, 2 H), 9.78 (s, 1 H) ppm; ^13^C-NMR (100 MHz, DMSO-*d*_6_): δ 24.7, 25.7, 28.4, 29.4, 32.5, 32.8, 58.7, 106.7, 110.3, 118.6, 119.6, 120.1, 121.7, 123.9, 124.9, 128.6, 128.8, 136.9, 137.3, 146.7, 167.4, 176.4 ppm; IR (Nujol, cm^−1^): ν_max_ 3251, 1739, 1708; MS (ESI): *m/z* 401 (M + H)^+^; anal. calcd. for C_24_H_24_N_4_O_2_ (400.47): C 71.98, H 6.04, N 13.99; found: C 71.90, H 6.15, N 14.15.

*3a-(1-methyl-1H-indol-3-yl)-3a,4,5,6,7,8-hexahydrocyclohepta[c]pyrazol-3(2H)-one* (**4e**). Yield 97% (54.6 mg) as a white solid; m.p. 250–252 °C; ^1^H-NMR (400 MHz, DMSO-*d*_6_): δ 1.20–1.31 (m, 1 H), 1.41–1.63 (m, 4 H), 1.77–1.85 (m, 1 H), 1.98–2.05 (m, 1 H), 2.25–2.34 (m, 2 H), 2.56–2.63 (m, 1 H), 3.77 (s, 3 H), 6.98 (dt, *J*_1_ = 8.0 Hz, *J*_2_ = 0.8 Hz, 1 H), 7.14 (dt, *J*_1_ = 8.0 Hz, *J*_2_ = 0.8 Hz, 1 H), 7.32 (d, *J* = 8.0 Hz, 1 H), 7.37 (s, 1 H), 7.41 (d, *J* = 8.0 Hz, 1 H), 11.14 (s, 1 H) ppm; ^13^C-NMR (100 MHz, DMSO-*d*_6_): δ 24.8, 26.3, 28.7, 29.4, 32.4, 32.9, 55.4, 108.3, 109.9, 18.7, 119.0, 121.3, 125.2, 128.1, 136.8, 165.9, 178.9 ppm; IR (Nujol, cm^−1^): ν_max_ 3165, 1714; MS (ESI): *m/z* 282 [M + H]^+^; anal. calcd. for C_17_H_19_N_3_O (281.35): C 72.57, H 6.81, N 14.94; found: C 72.43, H 6.90, N 15.02.

*3a-(1-ethyl-1H-indol-3-yl)-3-oxo-3a,4,5,6,7,8-hexahydrocyclohepta[c]pyrazole-2(3H)-carboxamide* (**4f**). Yield 93% (62.9 mg) as a white solid; m.p. 191-193 °C; ^1^H-NMR (400 MHz, DMSO-*d*_6_): δ 1.27–1.38 (m, 1 H), 1.36 (t, *J* = 7.2 Hz, 3 H), 1.45–1.68 (m, 4 H), 1.77–1.85 (m, 1 H), 2.16–2.24 (m, 1 H), 2.40–2.46 (m, 2 H), 2.68–2.75 (m, 1 H), 4.21 (q, *J* = 7.2 Hz, 2 H), 7.01 (dt, *J*_1_ = 7.2 Hz, *J*_2_ = 0.8 Hz, 1 H), 7.16 (dt, *J*_1_ = 7.2 Hz, *J*_2_ = 0.8 Hz, 1 H), 7.25 (br, 1 H), 7.31 (d, *J* = 8.0 Hz, 1 H), 7.49 (d, *J* = 8.0 Hz, 1 H), 7.51 (s, 1 H), 7.53 (br, 1 H) ppm; ^13^C-NMR (100 MHz, DMSO-*d*_6_): δ 15.3, 24.7, 25.9, 28.5, 29.3, 32.8, 40.4, 58.5, 107.1, 110.3, 118.5, 119.5, 121.6, 124.9, 126.9, 135.9, 149.4, 166.7, 176.7 ppm; IR (Nujol, cm^−1^): ν_max_ 3384, 3175, 1734, 1683; MS (ESI): *m/z* 339 [M + H]^+^; anal. calcd. for C_19_H_22_N_4_O_2_ (338.40): C 67.44, H 6.55, N 16.56.; found: C 67.29, H 6.62, N 16.43.

*3a-(1-benzyl-1H-indol-3-yl)-3-oxo-3a,4,5,6,7,8-hexahydrocyclohepta[c]pyrazole-2(3H)-carboxamide* (**4g**). Yield quant. (80.0 mg) as a white solid; m.p. 181-183 °C; ^1^H-NMR (400 MHz, DMSO-*d*_6_): δ 1.30–1.41 (m, 1 H), 1.44–1.67 (m, 4 H), 1.77–1.83 (m, 1 H), 2.14–2.21 (m, 1 H), 2.43–2.49 (m, 2 H), 2.71–2.77 (m, 1 H), 5.44 (s, 2 H), 7.02 (t, *J* = 8.0 Hz, 1 H), 7.12 (t, *J* = 8.0 Hz, 1 H), 7.19–7.33 (m, 6 H), 7.35 (d, *J* = 8.0 Hz, 1 H), 7.45 (d, *J* = 8.0 Hz, 1 H), 7.59 (br, 1 H), 7.70 (s, 1 H) ppm; ^13^C-NMR (100 MHz, DMSO-*d*_6_): δ 24.8, 25.7, 28.4, 29.4, 32.9, 49.2, 58.5, 107.5, 110.8, 118.6, 119.7, 121.8, 125.2, 126.9, 127.4, 128.2, 128.5, 136.3, 137.9, 149.4, 166.7, 176.6 ppm; IR (Nujol, cm^−1^): ν_max_ 3409, 3257, 1759, 1724; MS (ESI): *m/z* 401 [M + H]^+^; anal. calcd. for C_24_H_24_N_4_O_2_ (400.47): C 71.98, H 6.04, N 13.99; found: C 72.12, H 5.95, N 13.85.

*3a-(1H-indol-3-yl)-3-oxo-3a,4,5,6,7,8-hexahydrocyclohepta[c]pyrazole-2(3H)-carboxamide* (**4h**). Yield quant. (62.1 mg) as a white solid; m.p. 201–203 °C; ^1^H-NMR (400 MHz, DMSO-*d*_6_): δ 1.17–1.81 (m, 6 H), 2.14–2.22 (m, 1 H), 2.30–2.45 (m, 2 H), 2.68–2.82 (m, 1 H), 6.98–7.57 (m, 7 H), 11.35 (s, 1 H) ppm; ^13^C-NMR (100 MHz, DMSO-*d*_6_): δ 24.7, 26.0, 28.5, 29.3, 32.7, 58.6, 107.8, 112.0, 118.2, 119.4, 121.6, 124.4, 124.5, 136.6, 149.5, 166.8, 176.8 ppm; IR (Nujol, cm^−1^): ν_max_ 3404, 3287, 3257, 1734, 1703; MS (ESI): *m/z* 311 [M + H]^+^; anal. calcd. for C_17_H_18_N_4_O_2_ (310.35): C 65.79, H 5.85, N 18.05; found: C 65.94, H 5.79, N 17.91.

*3a-(5-methyl-1-propyl-1H-indol-3-yl)-3-oxo-3a,4,5,6,7,8-hexahydrocyclohepta[c]pyrazole-2(3H)-carboxamide* (**4i**). Yield quant. (73.3 mg) as a white solid; m.p. 195–197 °C; ^1^H-NMR (400 MHz, DMSO-*d*_6_): δ 0.80 (t, *J* = 7.2 Hz, 3 H), 1.30–1.41 (m, 1 H), 1.44–1.63 (m, 4 H), 1.72 (sex, *J* = 7.2 Hz, 2 H), 1.79–1.86 (m, 1 H), 2.10–2.18 (m, 1 H), 2.33 (s, 3 H), 2.38–2.47 (m, 2 H), 2.67–2.78 (m, 1 H), 4.10 (t, *J* = 7.2 Hz, 2 H), 6.97 (d, *J* = 8.4 Hz, 1 H), 7.10 (s, 1 H), 7.28 (br, 1 H), 7.37 (d, *J* = 8.4 Hz, 1 H), 7.43 (s, 1 H), 7.57 (br, 1 H) ppm; ^13^C-NMR (100 MHz, DMSO-*d*_6_): δ 11.1, 21.3, 22.9, 24.8, 25.7, 28.4, 29.4, 32.9, 47.1, 58.5, 106.2, 110.2, 118.1, 123.1, 125.2, 127.7, 127.9, 134.7, 149.5, 166.7, 176.7 ppm; IR (Nujol, cm^−1^): ν_max_ 3369, 3175, 1734, 1688; MS (ESI): *m/z* 367 [M + H]^+^; anal. calcd. for C_21_H_26_N_4_O_2_ (366.45): C 68.83, H 7.15, N 15.29; found: C 68.99, H 7.06, N 15.16.

*3a-(5-methyl-1H-indol-3-yl)-3-oxo-3a,4,5,6,7,8-hexahydrocyclohepta[c]pyrazole-2(3H)-carboxamide* (**4j**). Yield quant. (64.9 mg) as a white solid; m.p. 209–211 °C; ^1^H-NMR (400 MHz, DMSO-*d*_6_): δ 1.23–1.37 (m, 1 H), 1.40–1.69 (m, 4 H), 1.73–1.84 (m, 1 H), 2.15–2.23 (m, 1 H), 2.32 (s, 3 H), 2.39–2.45 (m, 2 H), 2.69–2.75 (m, 1 H), 6.93 (d, *J* = 8.4 Hz, 1 H), 7.09 (s, 1 H), 7.28 (d, *J* = 8.4 Hz, 1 H), 7.31 (br, 1 H), 7.37 (d, *J* = 2.4 Hz, 1 H), 7.56 (br, 1 H), 11.21 (s, 1 H) ppm; ^13^C-NMR (100 MHz, DMSO-*d*_6_): δ 21.4, 24.7, 25.9, 28.5, 29.3, 32.8, 58.6, 107.2, 111.7, 117.8, 123.1, 124.3, 124.7, 127.8, 134.9, 149.5, 166.8, 176.8 ppm; IR (Nujol, cm^−1^): ν_max_ 3399, 3328, 3272, 1729, 1714; MS (ESI): *m/z* 325 [M + H]^+^; anal. calcd. for C_18_H_20_N_4_O_2_ (324.37): C 66.65, H 6.21, N 17.27; found: C 66.52, H 6.13, N 17.39.

*3a-(6-chloro-1-methyl-1H-indol-3-yl)-3-oxo-3a,4,5,6,7,8-hexahydrocyclohepta[c]pyrazole-2(3H)-carboxamide* (**4k**). Yield 96% (68.9 mg) as a white solid; m.p. 229–231 °C; ^1^H-NMR (400 MHz, DMSO-*d*_6_): δ 1.29–1.35 (m, 1 H), 1.44–1.67 (m, 4 H), 1.72–1.82 (m, 1 H), 2.15–2.23 (m, 1 H), 2.34– 2.48 (m, 2 H), 269–2.76 (m, 1 H), 3.78 (s, 3 H), 7.07 (dd, *J*_1_ = 8.8 Hz, *J*_2_ = 2.0 Hz, 1 H), 7.23 (br, 1 H), 7.33 (d, *J* = 8.8 Hz, 1 H), 7.51 (s, 1 H), 7.57 (br, 1 H), 7.60 (d, *J* = 2.0 Hz, 1 H) ppm; ^13^C-NMR (100 MHz, DMSO-*d*_6_): δ 24.7, 25.9, 28.4, 29.3, 32.7, 32.8, 58.4, 107.4, 110.3, 119.8, 119.9, 123.5, 126.7, 129.6, 137.4, 149.3, 166.5, 176.3 ppm; IR (Nujol, cm^−1^): ν_max_ 3420, 3262, 1754, 1724; MS (ESI): *m/z* 359 [M + H]^+^; anal. calcd. for C_18_H_19_ClN_4_O_2_ (358.82): C 60.25, H 5.34, N 15.61; found: C 60.41, H 5.27, N 15.50.

*3a-(5-cyano-1-methyl-1H-indol-3-yl)-3-oxo-3a,4,5,6,7,8-hexahydrocyclohepta[c]pyrazole-2(3H)-carboxamide* (**4l**). Yield 99% (69.2 mg) as a white solid; m.p. 176-178 °C; ^1^H-NMR (400 MHz, DMSO-*d*_6_): δ 1.23–1.38 (m, 1 H), 1.40–1.79 (m, 5 H), 2.26–2.55 (m, 3 H), 2.76–2.82 (m, 1 H), 3.84 (s, 3 H), 7.25 (br, 1 H), 7.54 (d, *J* = 8.4 Hz, 1 H), 7.57 (br, 1 H), 7.65 (s, 1 H), 7.66 (d, *J* = 8.4 Hz, 1 H), 7.88 (s, 1 H) ppm; ^13^C-NMR (100 MHz, DMSO-*d*_6_): δ 24.6, 26.0, 28.5, 29.4, 32.7, 32.8, 58.4, 101.7, 108.5, 111.9, 120.3, 124.2, 124.3, 124.4, 131.1, 138.6, 149.3, 166.2, 176.0 ppm; IR (Nujol, cm^−1^): ν_max_ 3399, 3302, 1739, 1724; MS (ESI): *m/z* 350 [M + H]^+^; anal. calcd. for C_19_H_19_N_5_O_2_ (349.38): C 65.32, H 5.48, N 20.04; found: C 65.47, H 5.37, N 19.96.

*Methyl 3-(2-carbamoyl-3-oxo-2,3,3a,4,5,6,7,8-octahydrocyclohepta[c]pyrazol-3a-yl)-1-methyl-1H-indole-5-carboxylate* (**4m**). Yield 98% (74.9 mg) as a white solid; m.p. 201–203 °C; ^1^H-NMR (400 MHz, DMSO-*d*_6_): δ 1.25–1.38 (m, 1 H), 1.45–1.83 (m, 5 H), 2.22–2.43 (m, 2 H), 2.49–2.56 (m, 2 H), 2.73–2.85 (m, 1 H), 3.82 (s, 3 H), 3.84 (s, 3 H), 7.25 (br, 1 H), 7.55 (d, *J* = 8.8 Hz, 1 H), 7.59 (s, 1 H), 7.79 (dd, *J*_1_ = 8.8 Hz, *J*_2_ = 1.2 Hz, 1 H), 8.17 (d, *J* = 1.2 Hz, 1 H) ppm; ^13^C-NMR (100 MHz, DMSO-*d*_6_): δ 24.7, 26.0, 28.0, 29.4, 32.8, 33.0, 51.8, 58.5, 108.8, 110.4, 120.9, 121.6, 122.5, 124.4, 130.3, 139.3, 149.3, 166.3, 166.9, 176.3 ppm; IR (Nujol, cm^−1^): ν_max_ 3440, 3343, 1754, 1709; MS (ESI): *m/z* 383 [M + H]^+^; anal. calcd. for C_20_H_22_N_4_O_4_ (382.41): C 62.82, H 5.80, N 14.65; found: C 62.98, H 5.74, N 14.53.

*3a-(1-methyl-5-nitro-1H-indol-3-yl)-3-oxo-3a,4,5,6,7,8-hexahydrocyclohepta[c]pyrazole-2(3H)-carboxamide* (**4n**). Yield 98% (72.4 mg) as a yellow solid; m.p. 232–234 °C; ^1^H-NMR (400 MHz, DMSO-*d*_6_): δ 1.25–1.75 (m, 6 H), 2.27–2.44 (m, 2 H), 2.48–2.58 (m, 1 H), 2.72–2.88 (m, 1 H), 3.87 (s, 3 H), 7.25 (br, 1 H), 7.61 (br, 1 H), 7.68 (d, *J* = 8.4 Hz, 1 H), 7.75 (s, 1 H), 8.07 (d, *J* = 8.4 Hz, 1 H), 8.40 (s, 1 H) ppm; ^13^C-NMR (100 MHz, DMSO-*d*_6_): δ 24.6, 26.1, 28.5, 29.4, 33.0, 33.2, 58.4, 110.1, 111.2, 115.9, 116.9, 123.8, 132.3, 139.9, 140.9, 149.2, 166.1, 175.9 ppm; IR (Nujol, cm^−1^): ν_max_ 3399, 3308, 1739, 1719; MS (ESI): *m/z* 370 [M + H]^+^; anal. calcd. for C_18_H_19_N_5_O_4_ (369.37): C 58.53, H 5.18, N 18.96; found: 58.68, H 5.11, N 18.84.

*3a-(1-methyl-1H-indol-3-yl)-3-oxo-3,3a,4,5,6,7,8,9-octahydro-2H-cycloocta[c]pyrazole-2-carboxamide* (**4o**). Yield quant. (67.7 mg) as a white solid; m.p. 207–209 °C; ^1^H-NMR (400 MHz, DMSO-*d*_6_): δ 0.89–1.04 (m, 1 H), 1.36–1.79 (m, 7 H), 2.12–2.26 (m, 1 H), 2.42–2.54 (m, 3 H), 3.79 (s, 3 H), 7.01 (t, *J* = 7.6 Hz, 1 H), 7.07 (d, *J* = 7.6 Hz, 1 H), 7.17 (t, *J* = 7.6 Hz, 1 H), 7.37 (br, 1 H), 7.44 (d, *J* = 7.6 Hz, 1 H), 7.57 (s, 1 H), 7.68 (br, 1 H) ppm; ^13^C-NMR (100 MHz, DMSO-*d*_6_): δ 22.4, 25.1, 25.5, 28.0, 28.9, 31.3, 32.6, 57.9, 107.5, 110.3, 117.3, 119.6, 121.7, 124.7, 128.8, 136.9, 149.3, 167.1, 176.9 ppm; IR (Nujol, cm^−1^): ν_max_ 3404, 3272, 1739, 1698; MS (ESI): *m/z* 339 [M + H]^+^; anal. calcd. for C_19_H_22_N_4_O_2_ (338.40): C 67.44, H 6.55, N 16.56; found: C 67.29, H 6.48, N 16.67.

*3a-(1H-indol-3-yl)-3-oxo-3,3a,4,5,6,7,8,9-octahydro-2H-cycloocta[c]pyrazole-2-carboxamide* (**4p**). Yield 90% (58.4 mg) as a white solid; m.p. 215–217 °C; ^1^H-NMR (400 MHz, DMSO-*d*_6_): δ 0.92–1.03 (m, 1 H), 1.38–1.80 (m, 7 H), 2.10–2.19 (m, 1 H), 2.41–2.51 (m, 2 H), 2.56–2.60 (m, 1 H), 6.96 (dt, *J*_1_ = 7.6 Hz, *J*_2_ = 1.2 Hz, 1 H), 7.02 (d, *J* = 7.6 Hz, 1 H), 7.09 (dt, *J*_1_ = 7.6 Hz, *J*_2_ = 1.2 Hz, 1 H), 7.36 (br, 1 H), 7.39 (d, *J* = 7.6 Hz, 1 H), 7.56 (d, *J* = 2.4 Hz, 1 H), 7.65 (br, 1 H), 11.37 (s, 1 H) ppm; ^13^C-NMR (100 MHz, DMSO-*d*_6_): δ 22.4, 25.1, 25.5, 28.0, 28.9, 31.2, 58.0, 108.3, 112.1, 117.1, 119.5, 121.6, 124.3, 124.7, 136.5, 149.4, 167.3, 177.0 ppm; IR (Nujol, cm^−1^): ν_max_ 3379, 3297, 3241, 1744, 1714; MS (ESI): *m/z* 325 [M + H]^+^; anal. calcd. for C_18_H_20_N_4_O_2_ (324.37): C 66.65, H 6.21, N 17.27; found: C 66.49, H 6.30, N 17.38.

### 3.4. One-Pot Procedure for the Synthesis of ***4c***

A mixture of *N*-methyl indole **A1** (1.4 mmol), cyclic azoalkene **B1** (1.0 mmol) and zinc dichloride (0.1 mmol) was stirred in dry dichloromethane (2 mL) at room temperature. After the disappearance of azoalkene **B1** (TLC check), TFA (2.5 eq) was directly added to the reaction medium. The reaction was refluxed until TLC indicated the disappearance of the intermediate (TLC check, 2 h). Removed the solvent under reduced pressure, the crude mixture was diluted with water and extracted with EtOAc (2 × 10 mL). The organic phase was dried with Na_2_SO_4_ and solvent was evaporated in vacuo. The crude mixture was purified by column chromatography on silica gel to afford product **4c** in 93% yield.

## 4. Conclusions

In conclusion, successful routes providing indole-based heterocycles such as fused indole-pyridazines and non-fused indole-pyrazol-5-ones have been accomplished. Mild and practical reaction conditions, wide substrate scope in conjunction with functional group tolerance make these protocols particularly attractive. Moreover, crude products are usually obtained in high purity and high yield by simple precipitation from the reaction medium. We expect that these methodologies and the chemistry described here would be a new addition to the indole chemistry and would find wide usage in both organic and medicinal chemistry.

## Figures and Tables

**Table 1:**
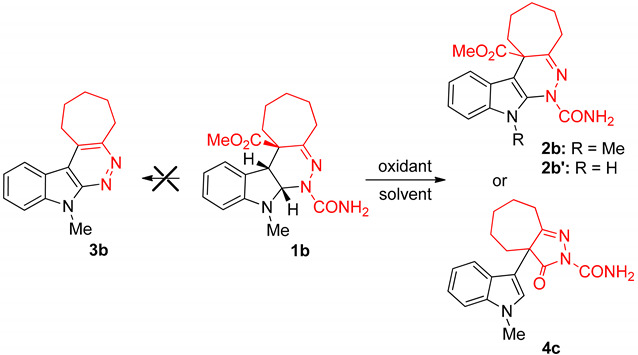
Optimization conditions.

Entry	Oxidant	Solvent	Temp. (°C)	Time (h)	2b(2b’)/4c	Yield (%)
1	DDQ (1 equiv)	dioxane	rt	2	**2b**	trace
2	DDQ (1 equiv)	toluene	reflux	24	**2b**	21
3	DDQ (1.5 equiv)	CH_2_Cl_2_	rt	15	**2b**	27
4	BQ (4 equiv)	toluene	reflux	12	–	–
5	NBS/TBPB (0.4 equiv)	CCl_4_	reflux	5	–	– ^a^
6	MnO_2_ (10 equiv)	benzene	70	48	**2b’**	40
7	MnO_2_ (25 equiv)	benzene	70	24	**2b’**	55
8	Na_2_Cr_2_O_7_ (1 equiv)	CHCl_3_	reflux	12	–	– ^a^
9	I_2_ (2 equiv)	CH_3_OH	reflux	24	–	– ^a^
10	PCC (3.3 equiv)	CH_2_Cl_2_	reflux	2	–	–
11	Pd/C (1 equiv)	AcOEt	reflux	24	–	– ^a^
12	CAN (1 equiv)	CH_2_Cl_2_	rt	24	**4c**	38
13	CuCl_2_∙2H_2_O (0.1 equiv)	DMSO	100	8	**4c**	80

^a^ Starting **1b** was recovered.

## Supplementary Materials

The following are available online: copies of ^1^H-NMR and ^13^C-MNR spectra of all newly synthesized compounds; copies of HMQC and HMBC of compound **2b’**.
