# Supplementary material for: Construction of Unusual Indole-Based Heterocycles from Tetrahydro-1H-pyridazino[3,4-b]indoles
Source: Molecules. 2020 Sep 9;25(18):4124. doi: 10.3390/molecules25184124 (PMC7571100; doi:10.3390/molecules25184124)
Supplement: Supplementary file 1 [file molecules-25-04124-s001.pdf]

Supplementary Material

Construction of Unusual Indole-Based Heterocycles from Tetrahydro-1*H*-pyridazino[3,4-*b*]indoles

Cecilia Ciccolini [1], Lucia De Crescentini [1], Fabio Mantellini [1], Giacomo Mari [1], Stefania Santeusanio [1], and Gianfranco Favi [1,*]

[1] Department of Biomolecular Sciences, Section of Chemistry and Pharmaceutical Technologies, University of Urbino "Carlo Bo", Via I Maggetti 24, 61029 Urbino (PU), Italy

* Correspondence: gianfranco.favi@uniurb.it

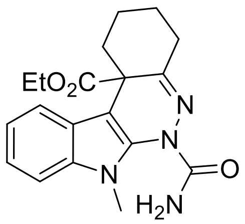
EtO2C
N
N
O
N
H2N

2a

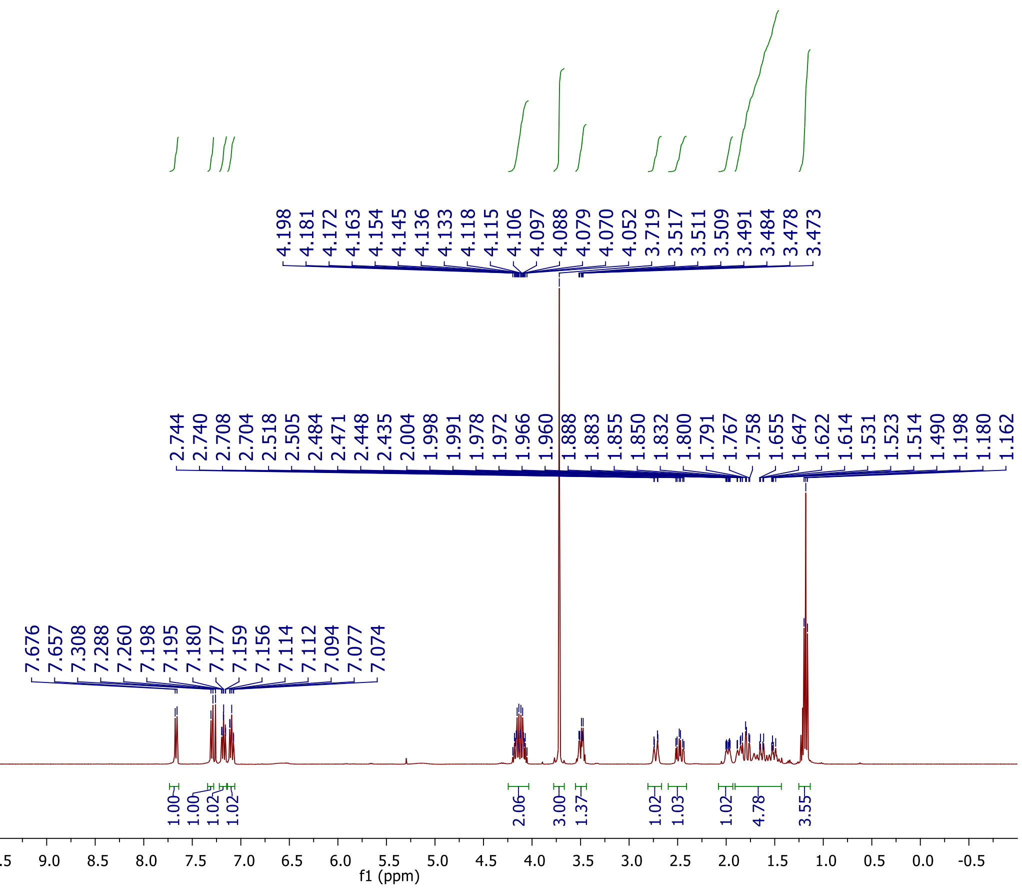
4.198
4.181
4.172
4.163
4.154
4.145
4.136
4.133
4.118
4.115
4.106
4.097
4.088
4.079
4.070
4.052
3.719
3.517
3.511
3.509
3.491
3.484
3.478
3.473
2.744
2.740
2.708
2.704
2.518
2.505
2.484
2.471
2.448
2.435
2.004
1.998
1.991
1.978
1.972
1.966
1.960
1.888
1.883
1.855
1.850
1.832
1.800
1.791
1.767
1.758
1.655
1.647
1.622
1.614
1.531
1.523
1.514
1.490
1.198
1.180
1.162
7.676
7.657
7.308
7.288
7.260
7.198
7.195
7.180
7.177
7.159
7.156
7.114
7.112
7.094
7.077
7.074
1.00
1.00
1.02
1.02
2.06
3.00
1.37
1.02
1.03
1.02
4.78
3.55
12.0 11.5 11.0 10.5 10.0 9.5 9.0 8.5 8.0 7.5 7.0 6.5 6.0 5.5 5.0 4.5 4.0 3.5 3.0 2.5 2.0 1.5 1.0 0.5 0.0 -0.5
f1 (ppm)

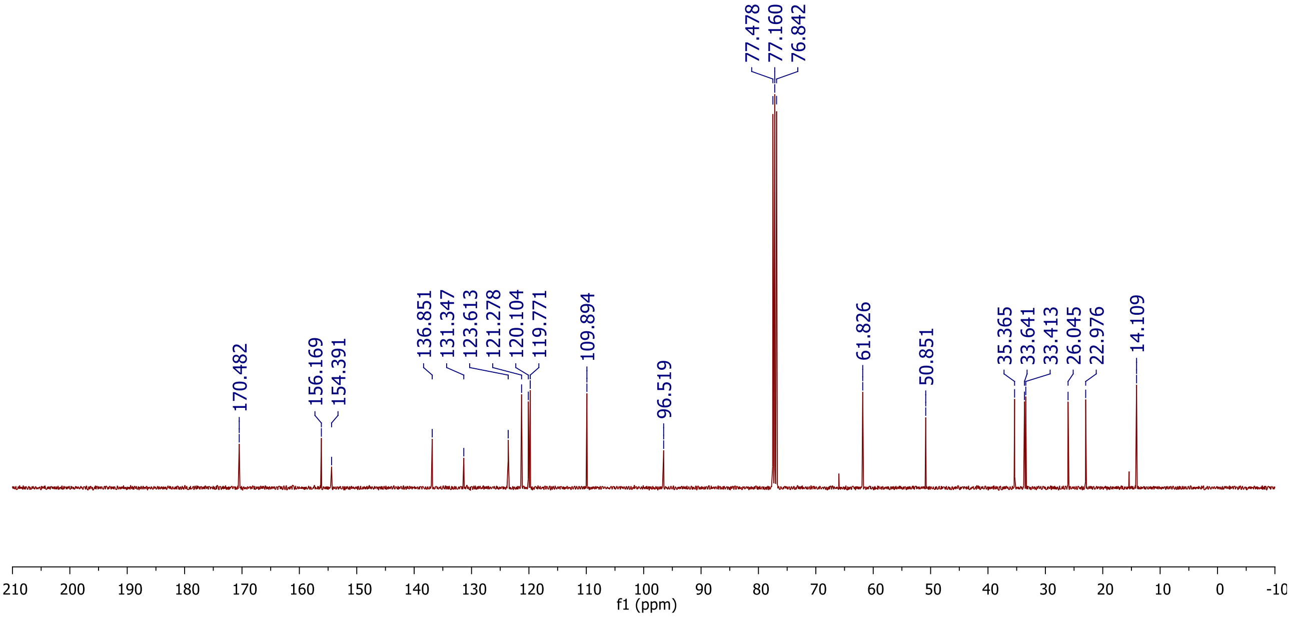
77.478
77.160
76.842
170.482
156.169
154.391
136.851
131.347
123.613
121.278
120.104
119.771
109.894
96.519
61.826
50.851
35.365
33.641
33.413
26.045
22.976
14.109
210 200 190 180 170 160 150 140 130 120 110 100 90 80 70 60 50 40 30 20 10 0 -10
f1 (ppm)

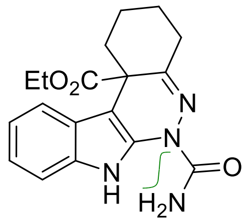
EtO2C
N
N
O
N
H
H2N

2a'

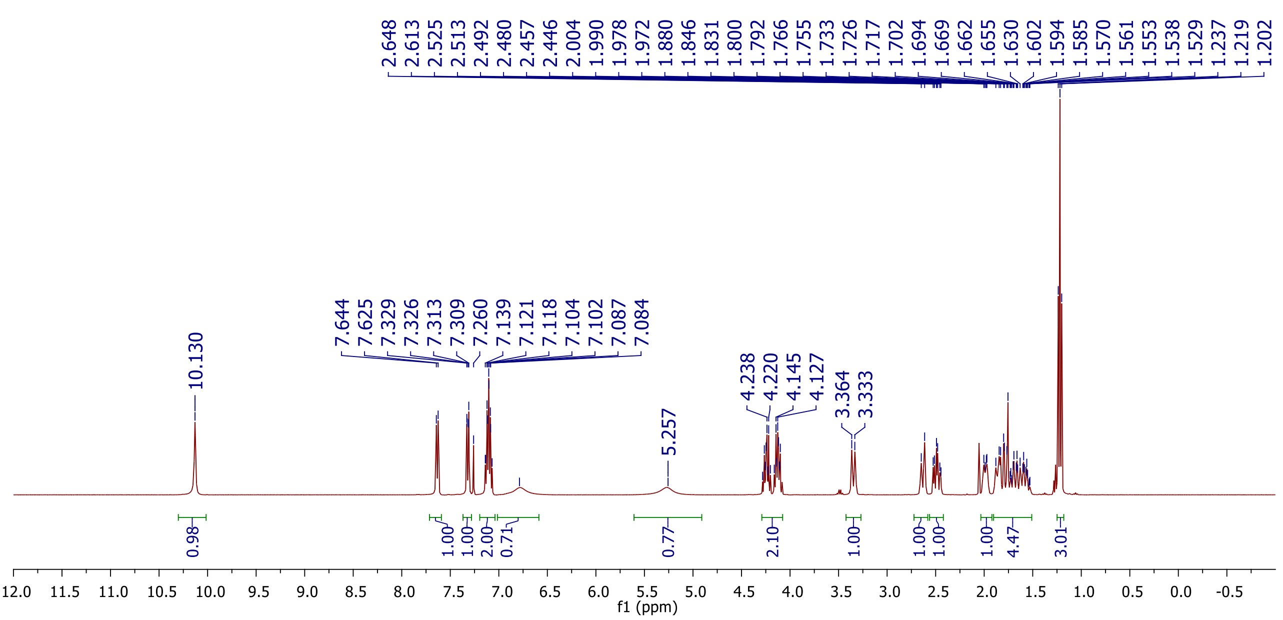
2.648 2.613 2.525 2.513 2.492 2.480 2.457 2.446 2.004 1.990 1.978 1.972 1.880 1.846 1.831 1.800 1.792 1.766 1.755 1.733 1.726 1.717 1.702 1.694 1.669 1.662 1.655 1.630 1.602 1.594 1.585 1.570 1.561 1.553 1.538 1.529 1.237 1.219 1.202
10.130
7.644 7.625 7.329 7.326 7.313 7.309 7.260 7.139 7.121 7.118 7.104 7.102 7.087 7.084
5.257
4.238 4.220 4.145 4.127
3.364 3.333
0.98 1.00 1.00 2.00 0.71 0.77 2.10 1.00 1.00 1.00 1.00 4.47 3.01
12.0 11.5 11.0 10.5 10.0 9.5 9.0 8.5 8.0 7.5 7.0 6.5 6.0 5.5 5.0 4.5 4.0 3.5 3.0 2.5 2.0 1.5 1.0 0.5 0.0 -0.5
f1 (ppm)

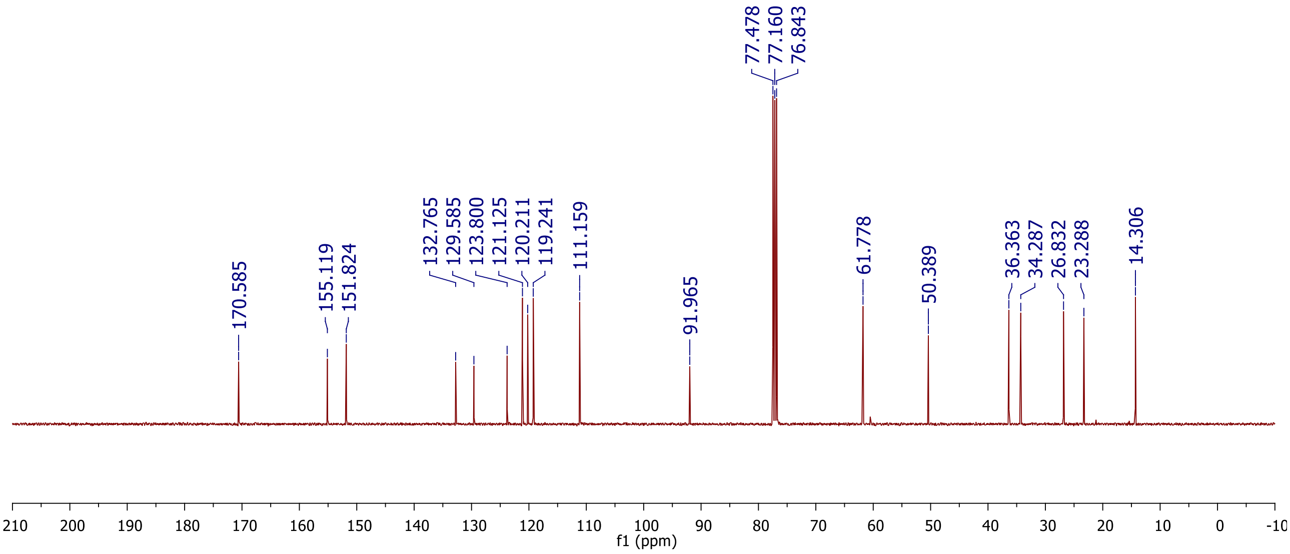
77.478 77.160 76.843
170.585
155.119 151.824
132.765 129.585 123.800 121.125 120.211 119.241
111.159
91.965
61.778
50.389
36.363 34.287 26.832 23.288
14.306
210 200 190 180 170 160 150 140 130 120 110 100 90 80 70 60 50 40 30 20 10 0 -10
f1 (ppm)

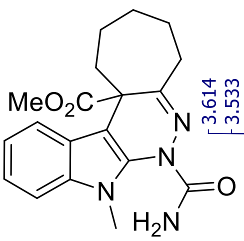
MeO2C
N
N
O
N
H2N

2b

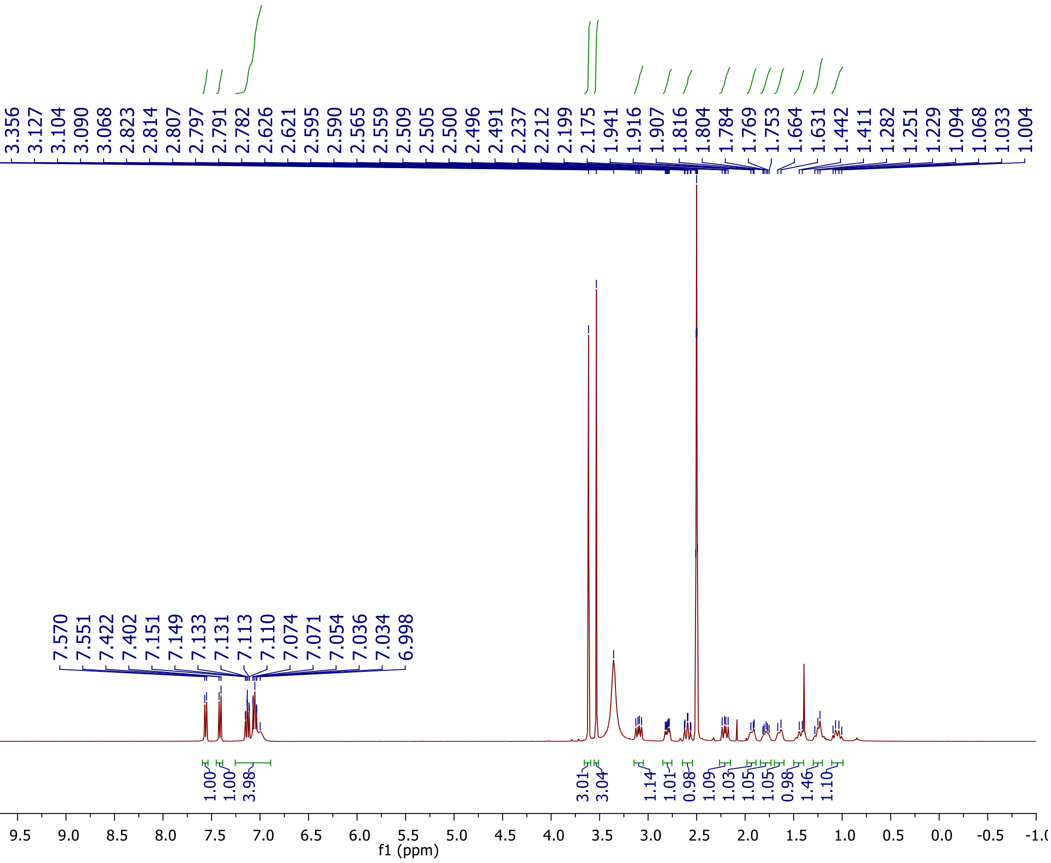
3.614
3.533
3.356
3.127
3.104
3.090
3.068
2.823
2.814
2.807
2.797
2.791
2.782
2.626
2.621
2.595
2.590
2.565
2.559
2.509
2.505
2.500
2.496
2.491
2.237
2.212
2.199
2.175
1.941
1.916
1.907
1.816
1.804
1.784
1.769
1.753
1.664
1.631
1.442
1.411
1.282
1.251
1.229
1.094
1.068
1.033
1.004
7.570
7.551
7.422
7.402
7.151
7.149
7.133
7.131
7.113
7.110
7.074
7.071
7.054
7.036
7.034
6.998
1.00
1.00
3.98
3.01
3.04
1.14
1.01
0.98
1.09
1.03
1.05
1.05
0.98
1.46
1.10
12.0 11.5 11.0 10.5 10.0 9.5 9.0 8.5 8.0 7.5 7.0 6.5 6.0 5.5 5.0 4.5 4.0 3.5 3.0 2.5 2.0 1.5 1.0 0.5 0.0 -0.5
f1 (ppm)

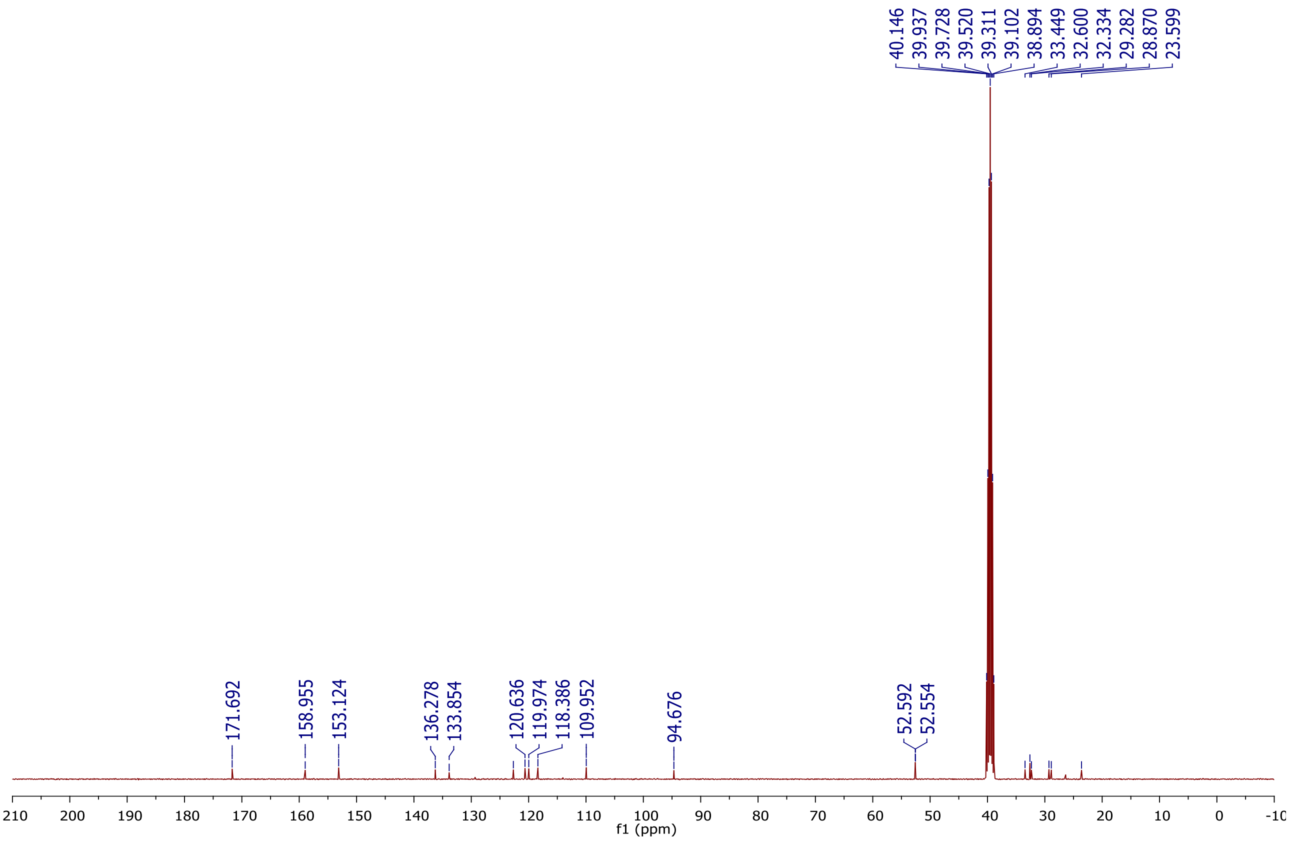
40.146
39.937
39.728
39.520
39.311
39.102
38.894
33.449
32.600
32.334
29.282
28.870
23.599
171.692
158.955
153.124
136.278
133.854
120.636
119.974
118.386
109.952
94.676
52.592
52.554
210 200 190 180 170 160 150 140 130 120 110 100 90 80 70 60 50 40 30 20 10 0
f1 (ppm)

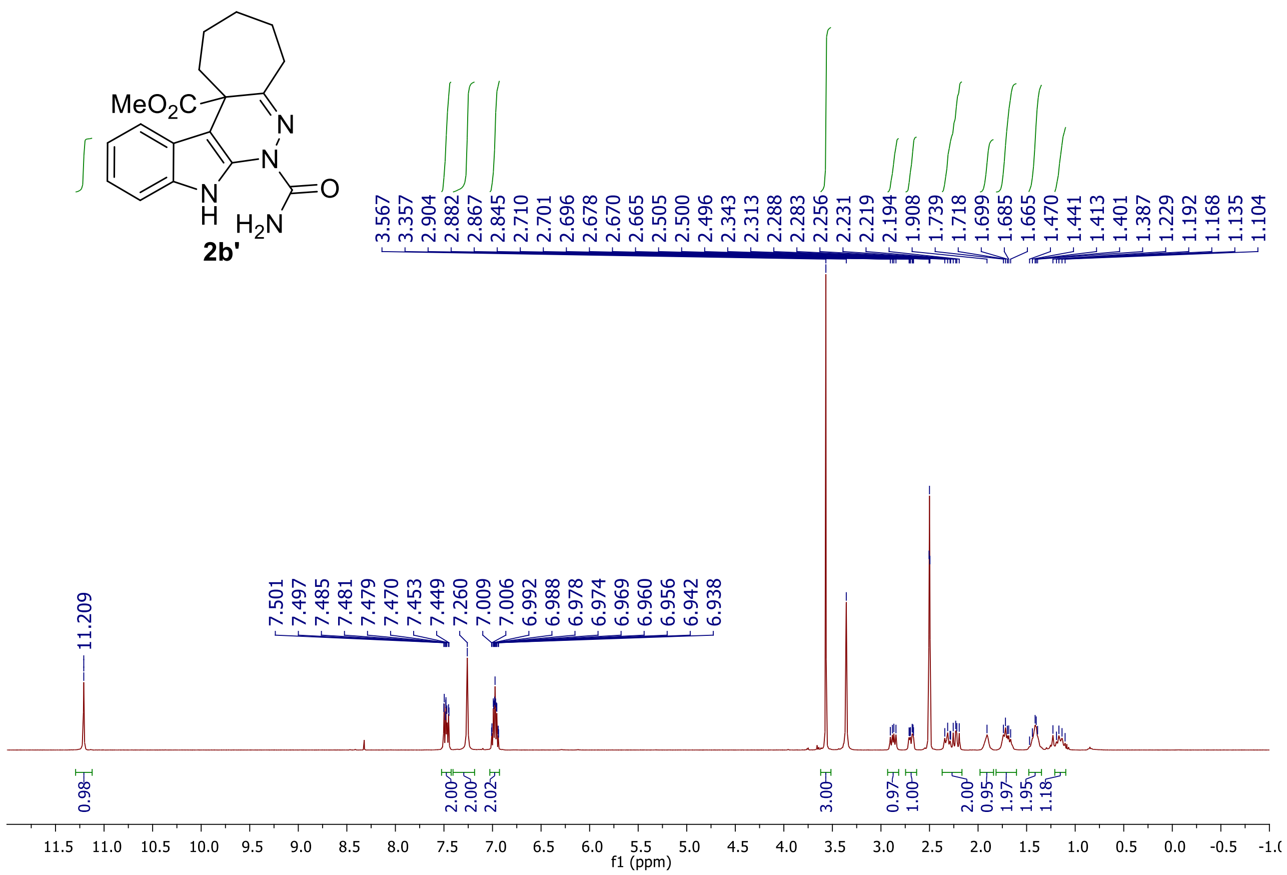
MeO2C
N
N
O
N
H
H2N
2b'
3.567 3.357 2.904 2.882 2.867 2.845 2.710 2.701 2.696 2.678 2.670 2.665 2.505 2.500 2.496 2.343 2.313 2.288 2.283 2.256 2.231 2.219 2.194 1.908 1.739 1.718 1.699 1.685 1.665 1.470 1.441 1.413 1.401 1.387 1.229 1.192 1.168 1.135 1.104
11.209
7.501 7.497 7.485 7.481 7.479 7.470 7.453 7.449 7.260 7.009 7.006 6.992 6.988 6.978 6.974 6.969 6.960 6.956 6.942 6.938
0.98
2.00 2.00 2.02
3.00
0.97 1.00
2.00 0.95 1.97 1.95 1.18
11.5 11.0 10.5 10.0 9.5 9.0 8.5 8.0 7.5 7.0 6.5 6.0 5.5 5.0 4.5 4.0 3.5 3.0 2.5 2.0 1.5 1.0 0.5 0.0 -0.5
f1 (ppm)

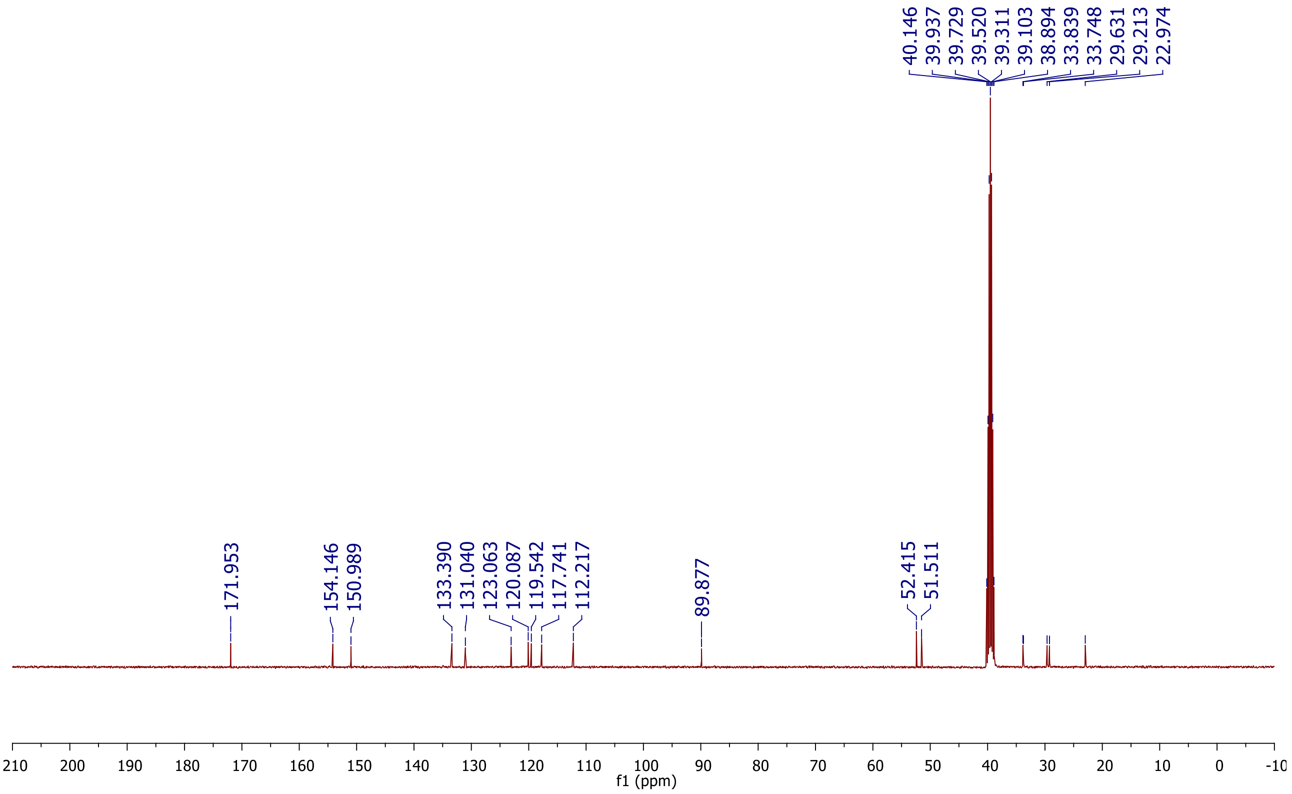
40.146 39.937 39.729 39.520 39.311 39.103 38.894 33.839 33.748 29.631 29.213 22.974
171.953
154.146 150.989
133.390 131.040 123.063 120.087 119.542 117.741 112.217
89.877
52.415 51.511
210 200 190 180 170 160 150 140 130 120 110 100 90 80 70 60 50 40 30 20 10 0 -10
f1 (ppm)

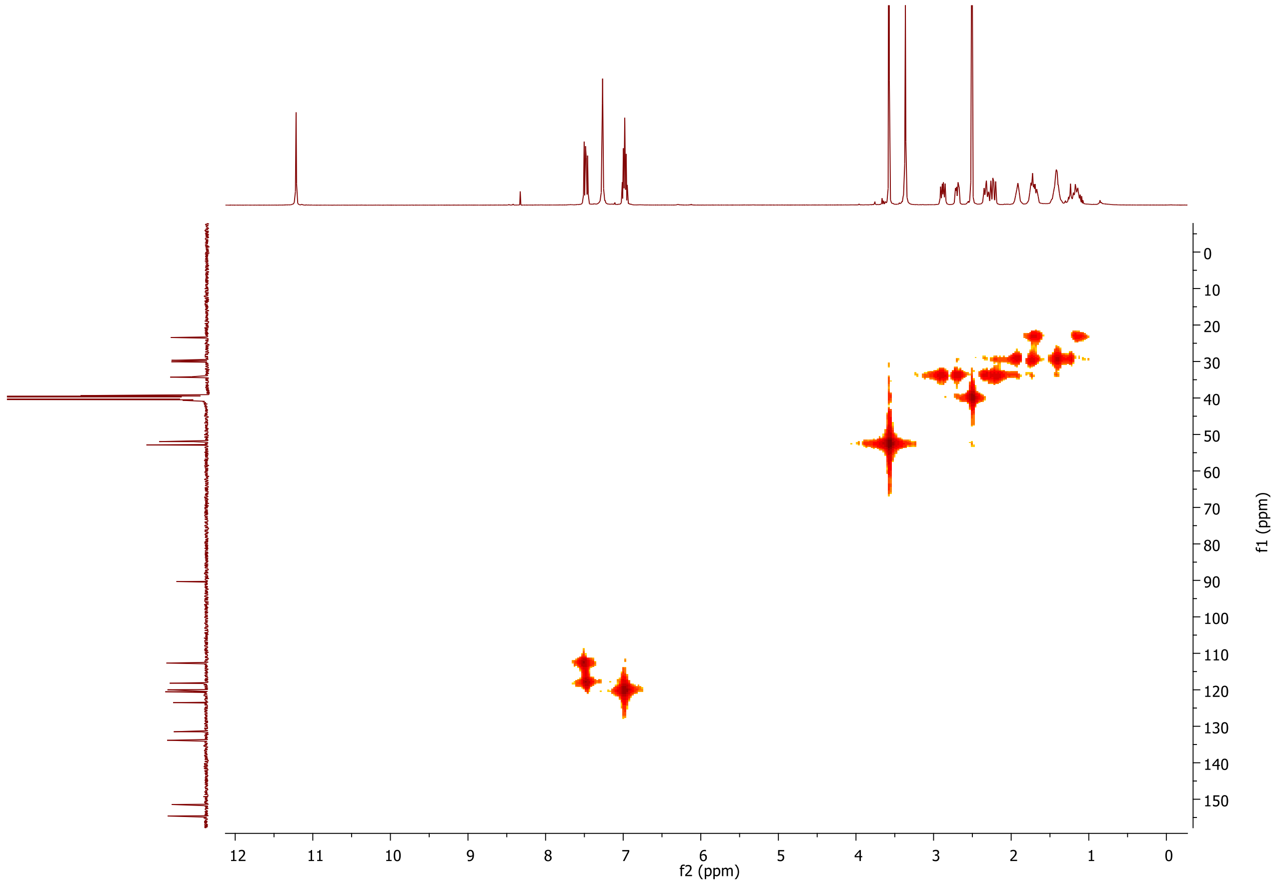

f2 (ppm)
f1 (ppm)

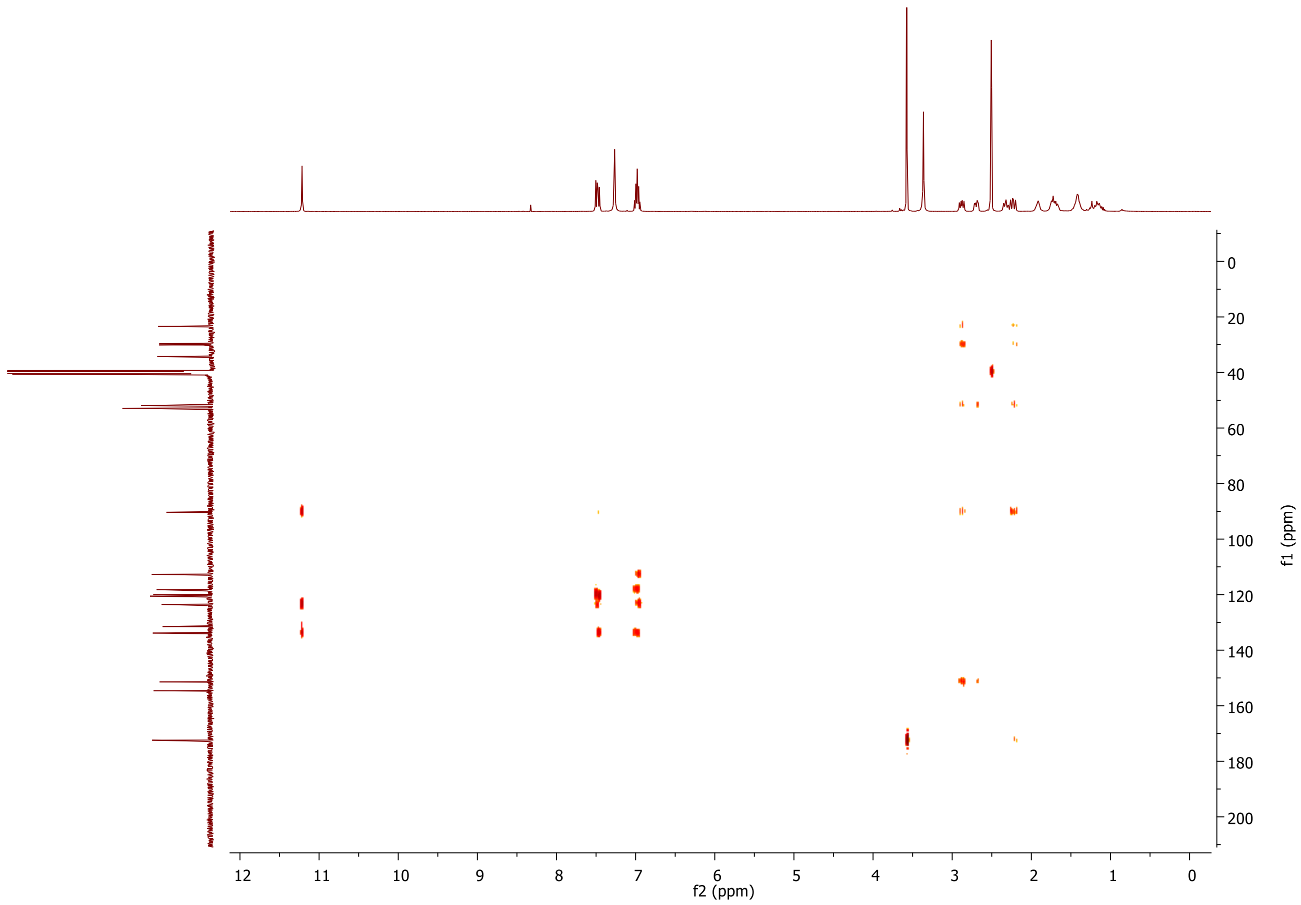

f2 (ppm)
f1 (ppm)

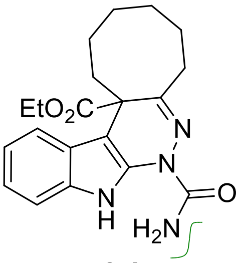
EtO2C
N
N
N
H
O
H2N

2c'

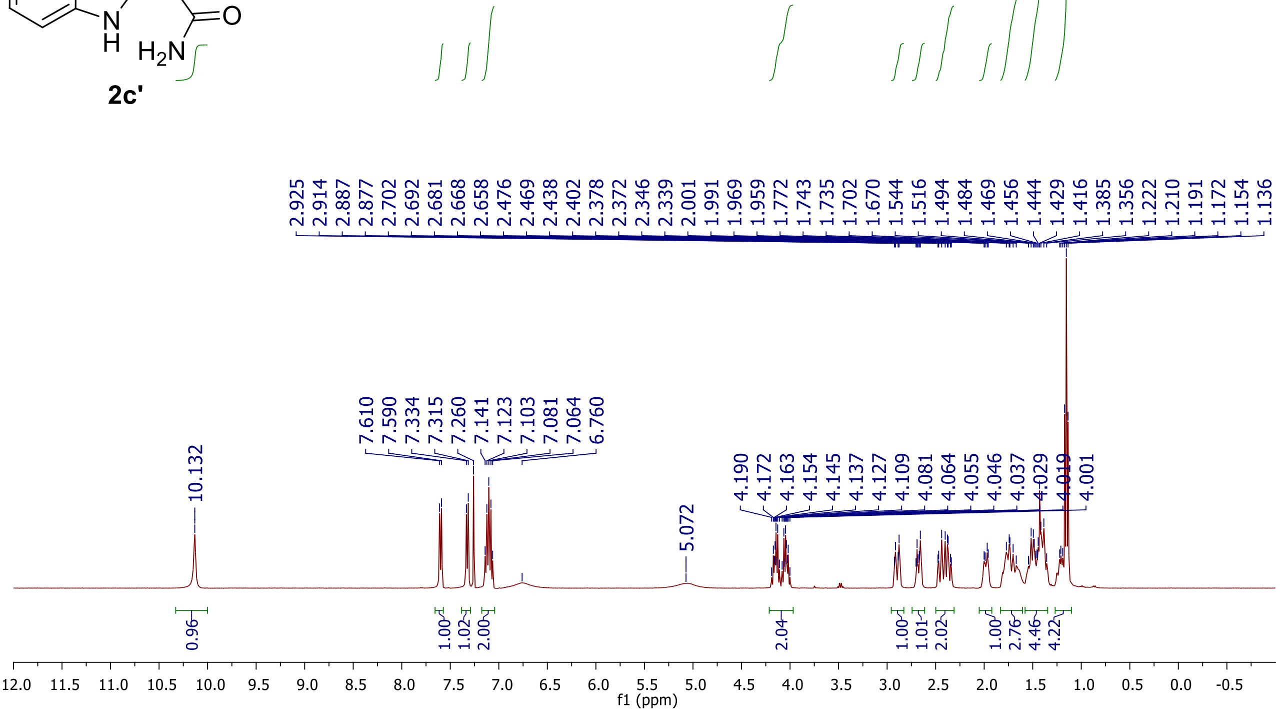
2.925 2.914 2.887 2.877 2.702 2.692 2.681 2.668 2.658 2.476 2.469 2.438 2.402 2.378 2.372 2.346 2.339 2.001 1.991 1.969 1.959 1.772 1.743 1.735 1.702 1.670 1.544 1.516 1.494 1.484 1.469 1.456 1.444 1.429 1.416 1.385 1.356 1.222 1.210 1.191 1.172 1.154 1.136
10.132
7.610 7.590 7.334 7.315 7.260 7.141 7.123 7.103 7.081 7.064 6.760
5.072
4.190 4.172 4.163 4.154 4.145 4.137 4.127 4.109 4.081 4.064 4.055 4.046 4.037 4.029 4.019 4.001
0.96
1.00 1.02 2.00
2.04
1.00 1.01 2.02
1.00 2.76 4.46 4.22
12.0 11.5 11.0 10.5 10.0 9.5 9.0 8.5 8.0 7.5 7.0 6.5 6.0 5.5 5.0 4.5 4.0 3.5 3.0 2.5 2.0 1.5 1.0 0.5 0.0 -0.5
f1 (ppm)

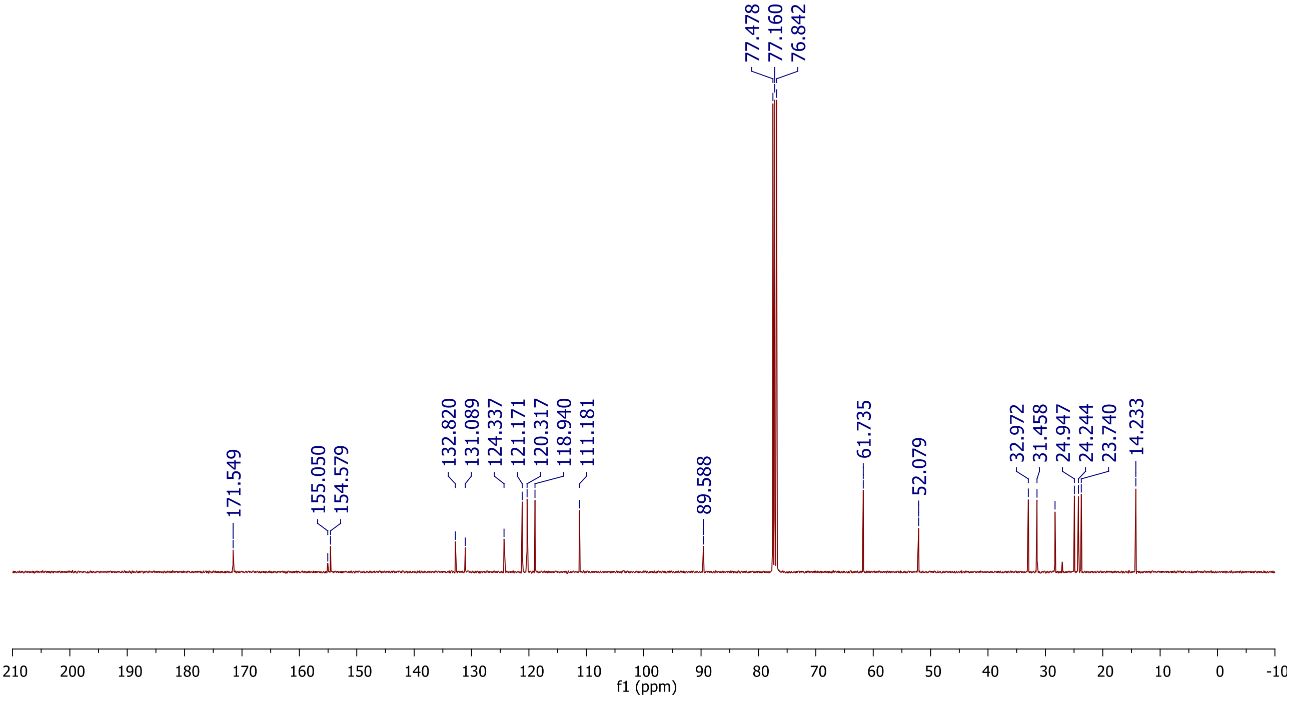
77.478 77.160 76.842
171.549
155.050 154.579
132.820 131.089 124.337 121.171 120.317 118.940 111.181
89.588
61.735
52.079
32.972 31.458 24.947 24.244 23.740 14.233
210 200 190 180 170 160 150 140 130 120 110 100 90 80 70 60 50 40 30 20 10 0 -10
f1 (ppm)

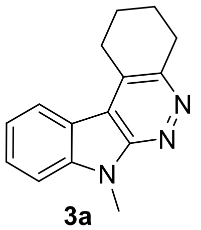
N
N
N

3a

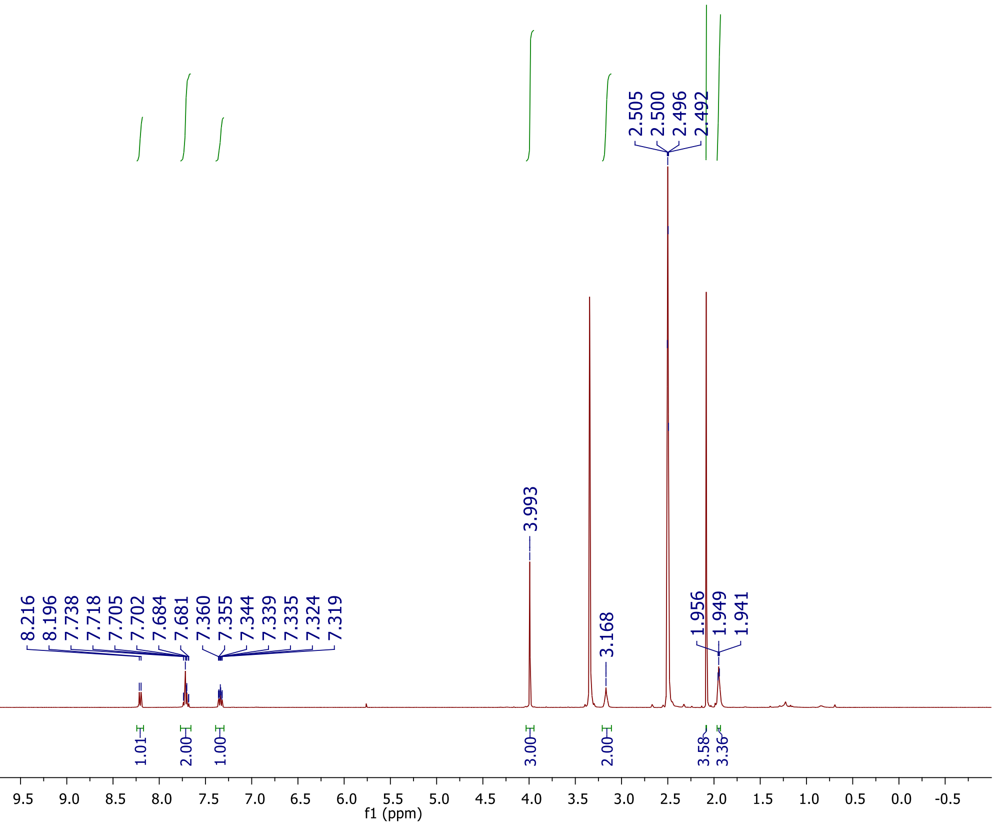
8.216
8.196
7.738
7.718
7.705
7.702
7.684
7.681
7.360
7.355
7.344
7.339
7.335
7.324
7.319
3.993
3.168
2.505
2.500
2.496
2.492
1.956
1.949
1.941
1.01
2.00
1.00
3.00
2.00
3.58
3.36
12.0 11.5 11.0 10.5 10.0 9.5 9.0 8.5 8.0 7.5 7.0 6.5 6.0 5.5 5.0 4.5 4.0 3.5 3.0 2.5 2.0 1.5 1.0 0.5 0.0 -0.5
f1 (ppm)

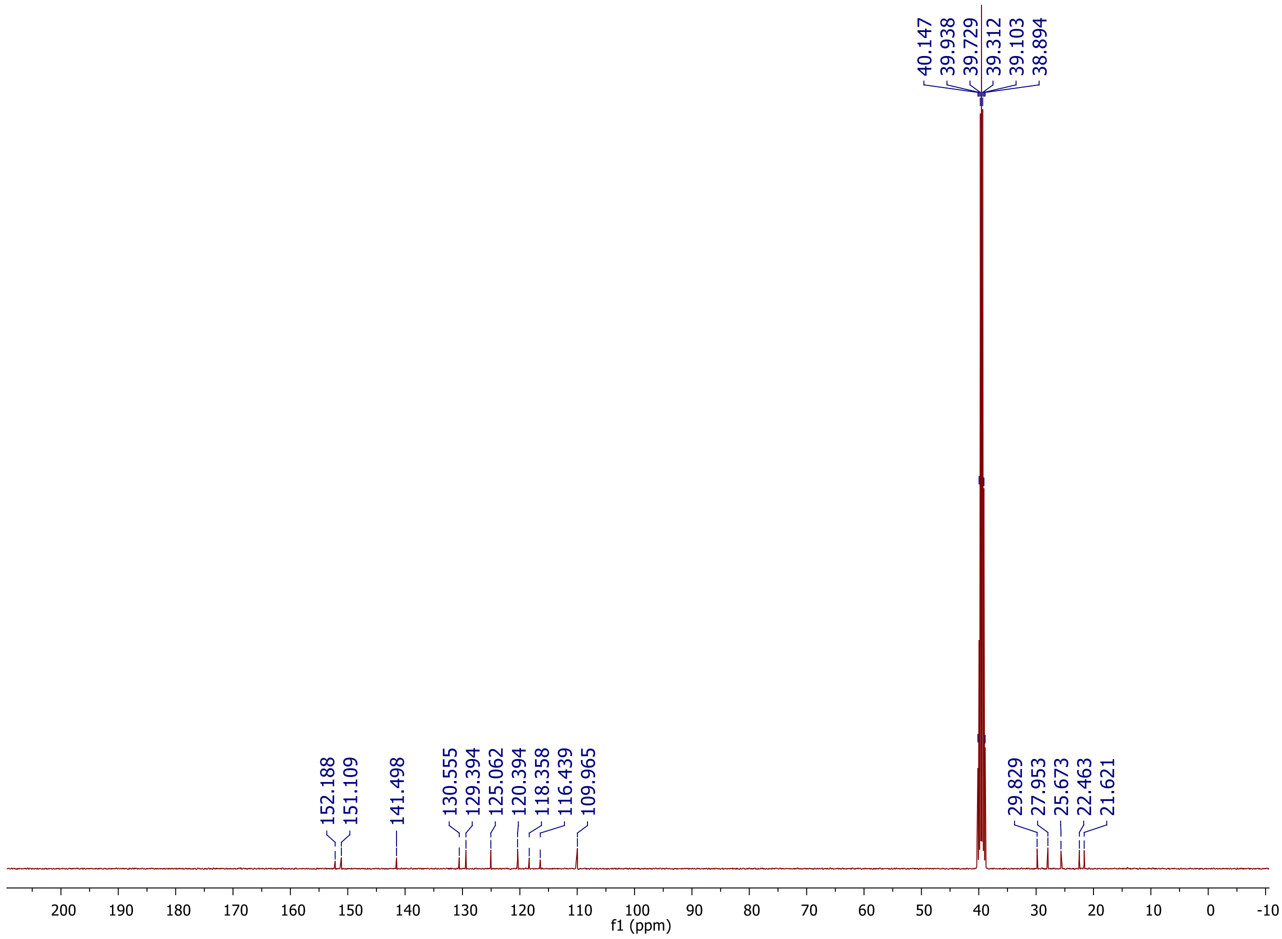
40.147
39.938
39.729
39.312
39.103
38.894
152.188
151.109
141.498
130.555
129.394
125.062
120.394
118.358
116.439
109.965
29.829
27.953
25.673
22.463
21.621
200 190 180 170 160 150 140 130 120 110 100 90 80 70 60 50 40 30 20 10 0 -10
f1 (ppm)

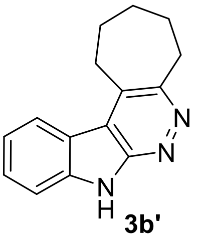
N
N
N
H

3b'

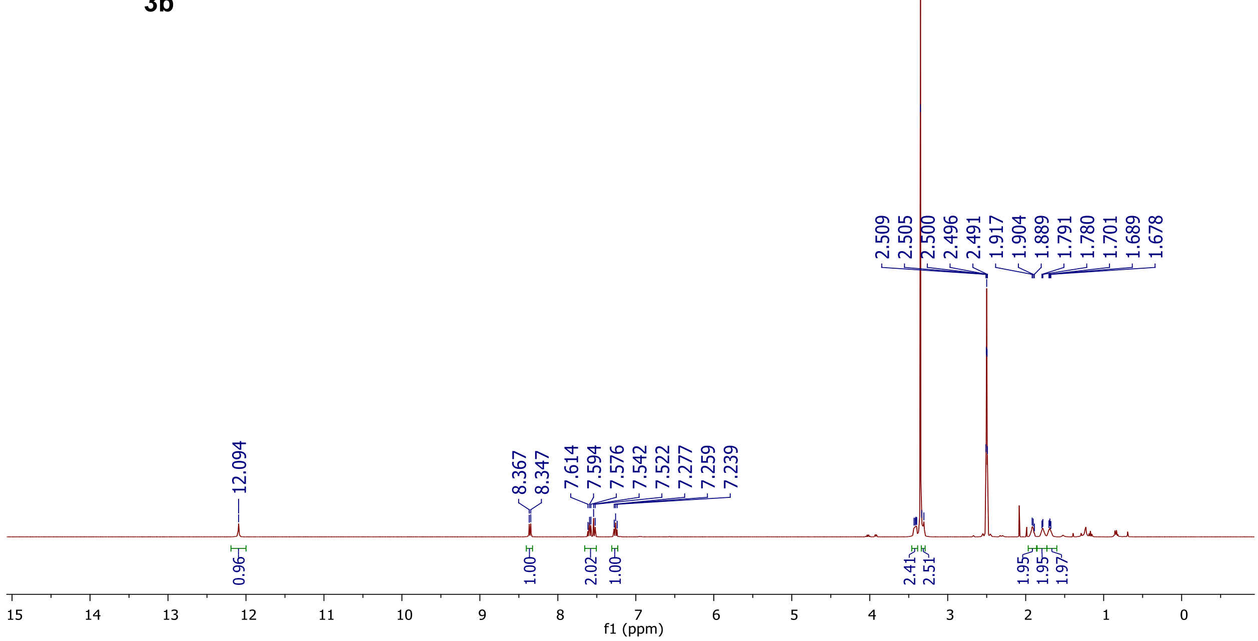
12.094
8.367
8.347
7.614
7.594
7.576
7.542
7.522
7.277
7.259
7.239
3.432
3.417
3.407
3.398
3.351
3.349
3.333
3.305
2.509
2.505
2.500
2.496
2.491
1.917
1.904
1.889
1.791
1.780
1.701
1.689
1.678
0.96
1.00
2.02
1.00
2.41
2.51
1.95
1.95
1.97
15 14 13 12 11 10 9 8 7 6 5 4 3 2 1 0
f1 (ppm)

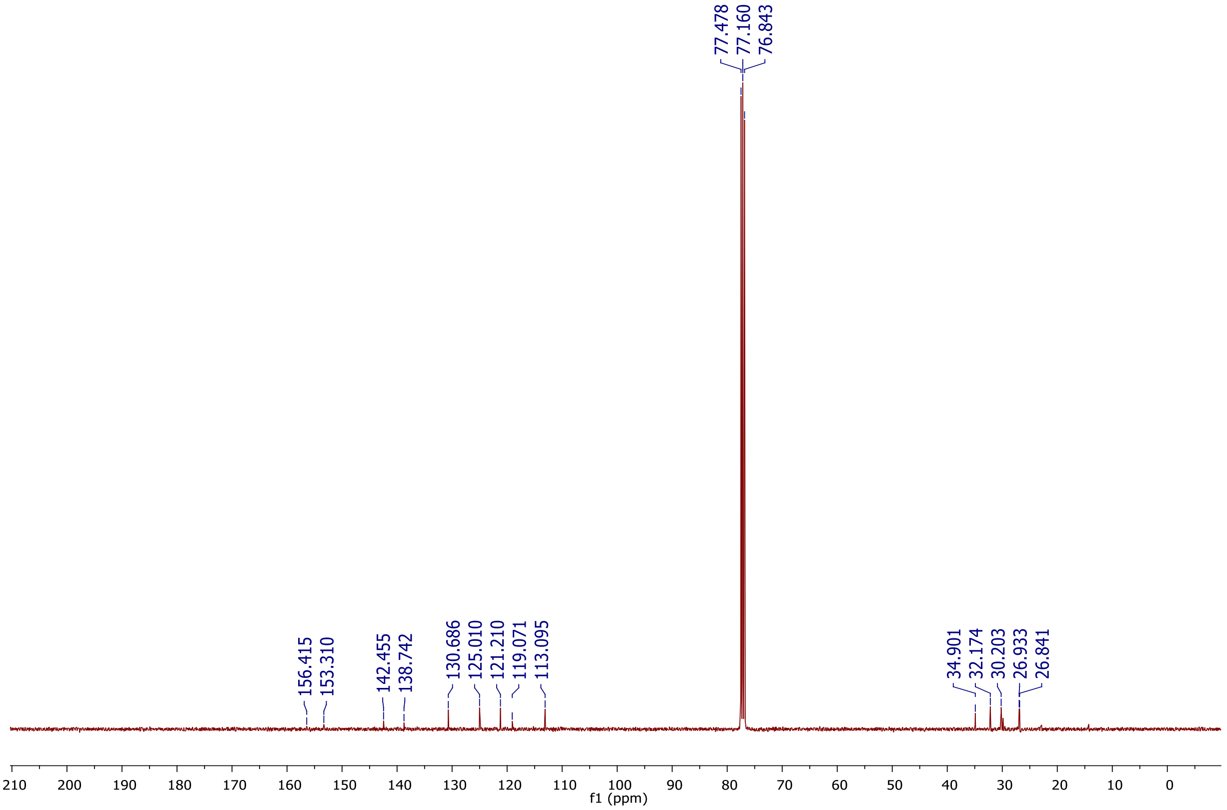
77.478
77.160
76.843
156.415
153.310
142.455
138.742
130.686
125.010
121.210
119.071
113.095
34.901
32.174
30.203
26.933
26.841
210 200 190 180 170 160 150 140 130 120 110 100 90 80 70 60 50 40 30 20 10 0
f1 (ppm)

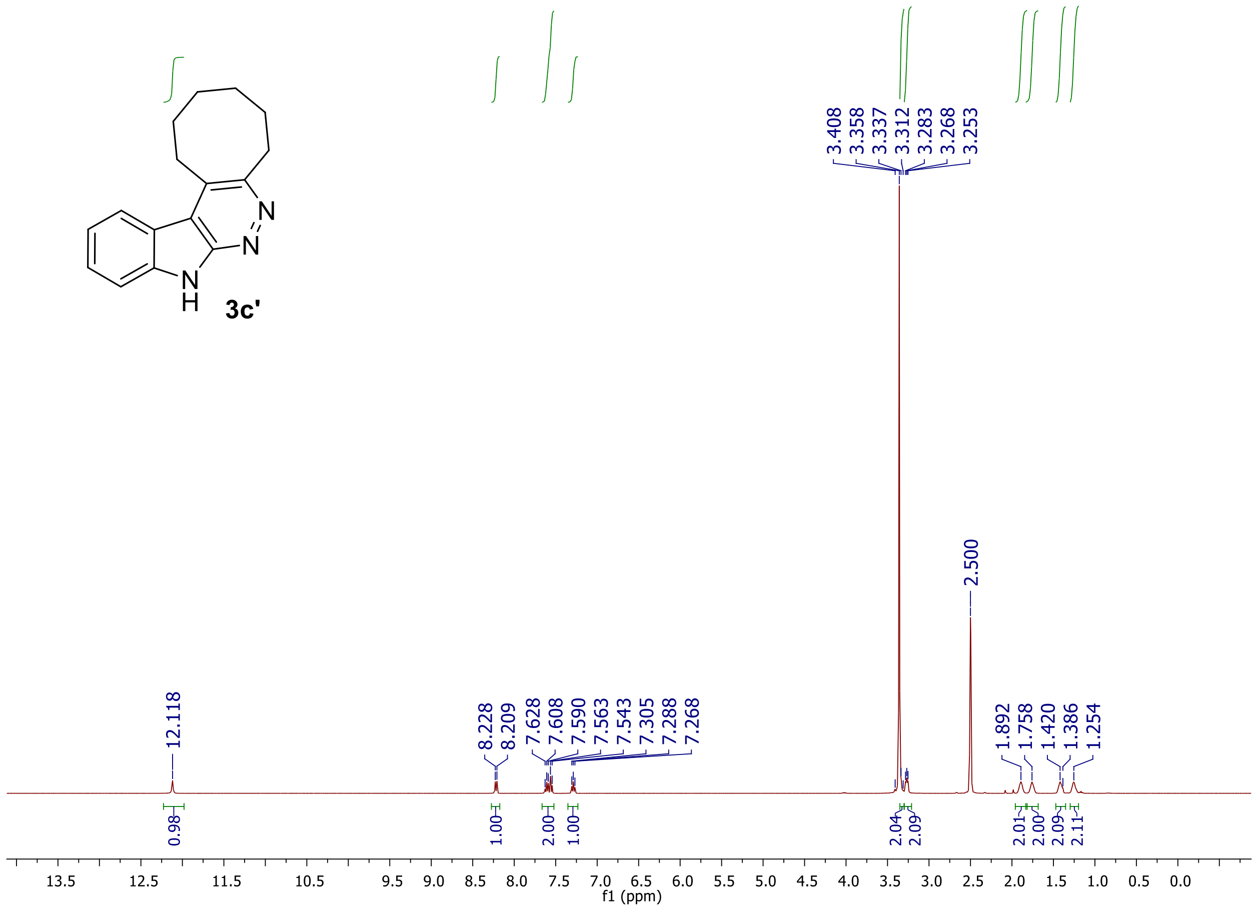
3c'
12.118
8.228
8.209
7.628
7.608
7.590
7.563
7.543
7.305
7.288
7.268
3.408
3.358
3.337
3.312
3.283
3.268
3.253
2.500
1.892
1.758
1.420
1.386
1.254
0.98
1.00
2.00
1.00
2.04
2.09
2.01
2.00
2.09
2.11
f1 (ppm)

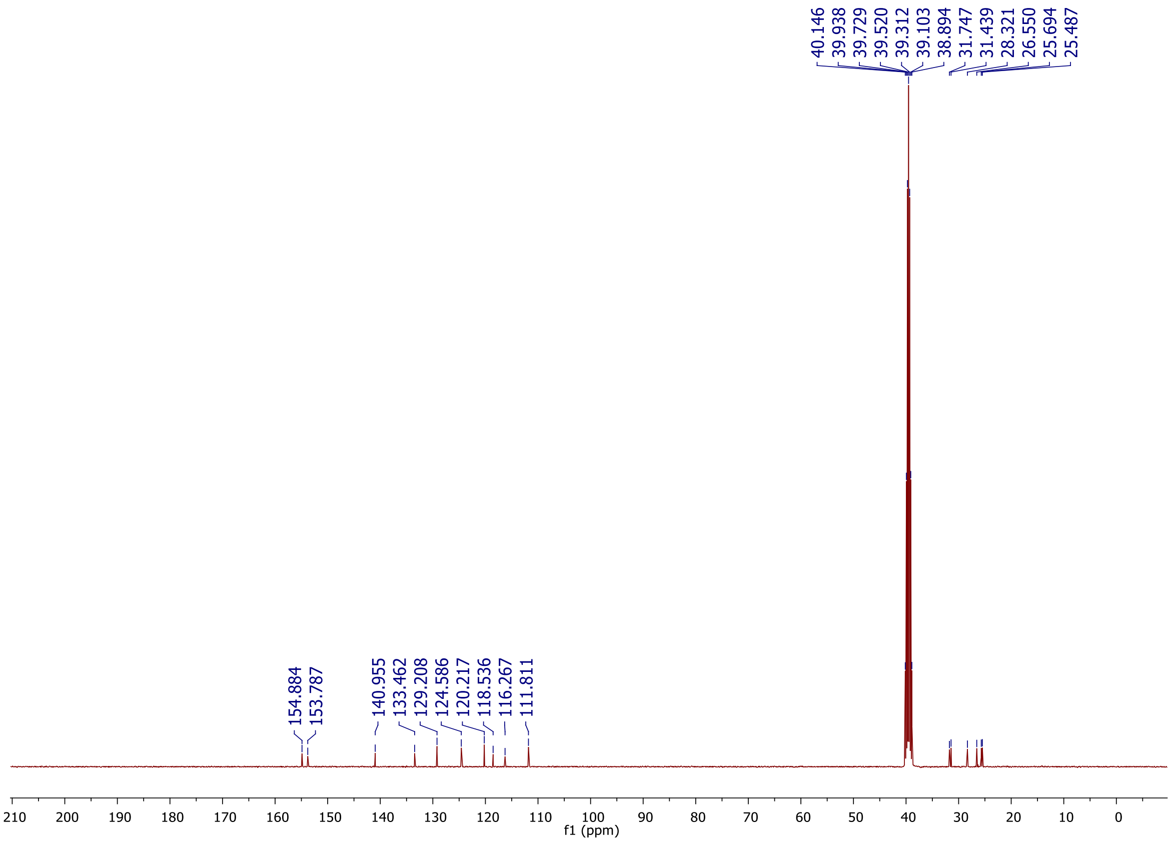
154.884
153.787
140.955
133.462
129.208
124.586
120.217
118.536
116.267
111.811
40.146
39.938
39.729
39.520
39.312
39.103
38.894
31.747
31.439
28.321
26.550
25.694
25.487
f1 (ppm)

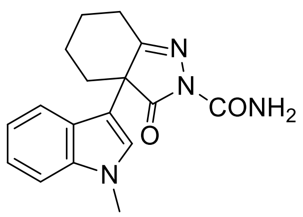
N
N
CONH2
O
N

4a

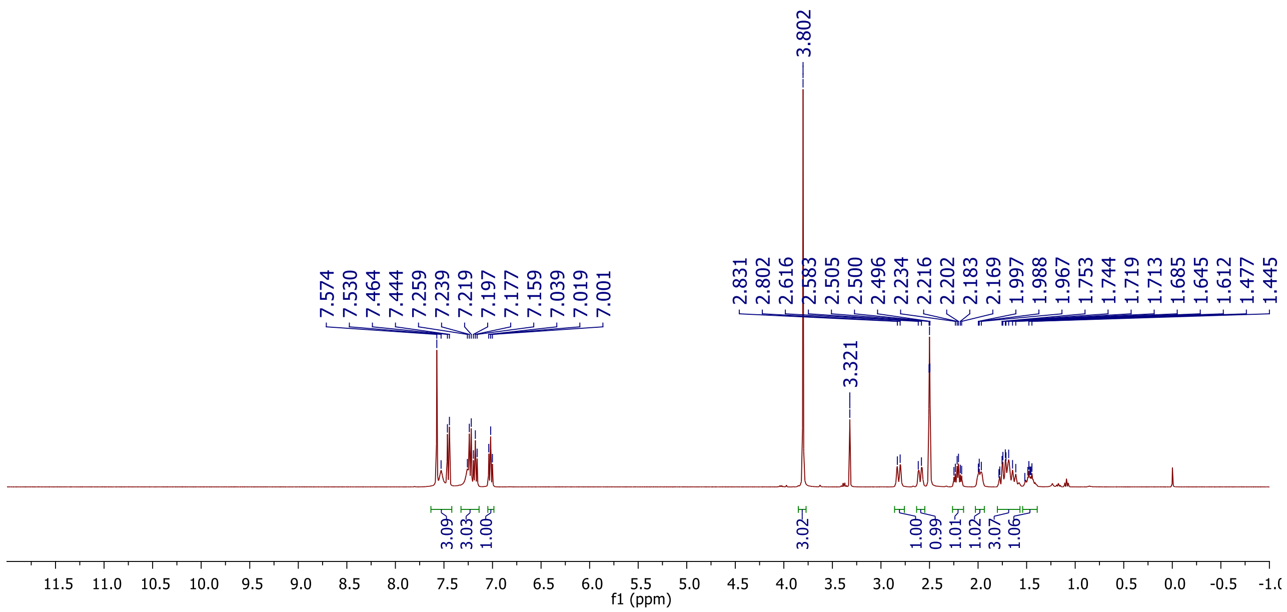
7.574 7.530 7.464 7.444 7.259 7.239 7.219 7.197 7.177 7.159 7.039 7.019 7.001
3.802
2.831 2.802 2.616 2.583 2.505 2.500 2.496 2.234 2.216 2.202 2.183 2.169 1.997 1.988 1.967 1.753 1.744 1.719 1.713 1.685 1.645 1.612 1.477 1.445
3.321
3.09 3.03 1.00
3.02
1.00 0.99 1.01 1.02 3.07 1.06
11.5 11.0 10.5 10.0 9.5 9.0 8.5 8.0 7.5 7.0 6.5 6.0 5.5 5.0 4.5 4.0 3.5 3.0 2.5 2.0 1.5 1.0 0.5 0.0 -0.5 -1.0
f1 (ppm)

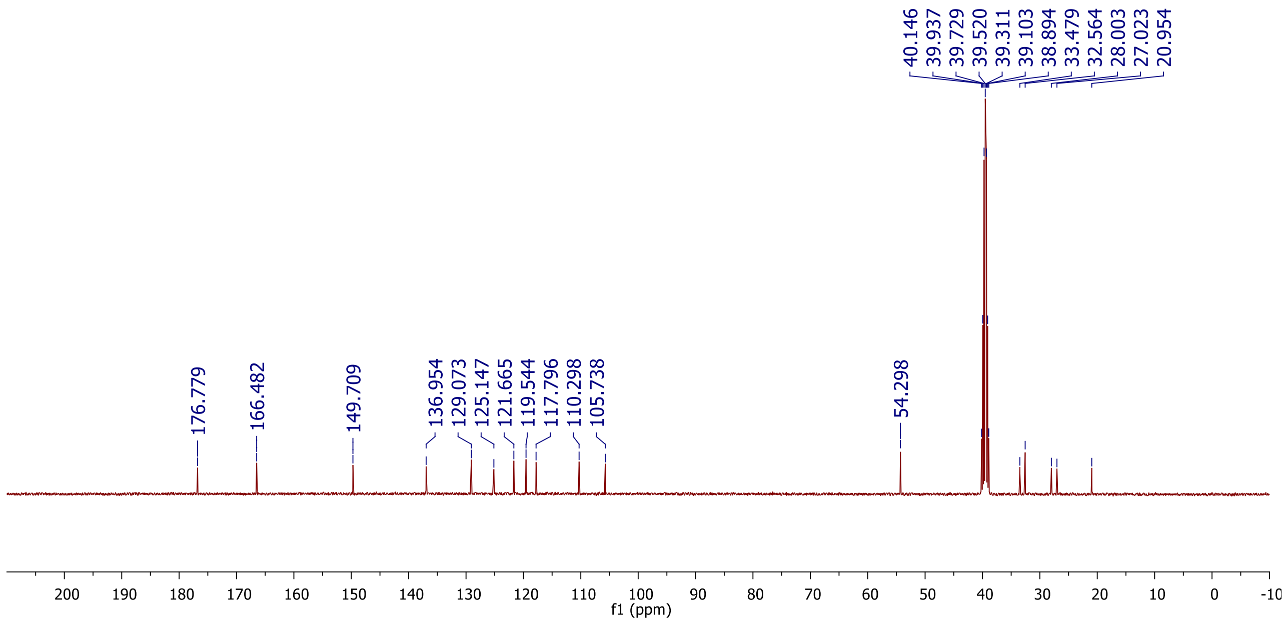
40.146 39.937 39.729 39.520 39.311 39.103 38.894 33.479 32.564 28.003 27.023 20.954
176.779
166.482
149.709
136.954 129.073 125.147 121.665 119.544 117.796 110.298 105.738
54.298
200 190 180 170 160 150 140 130 120 110 100 90 80 70 60 50 40 30 20 10 0 -10
f1 (ppm)

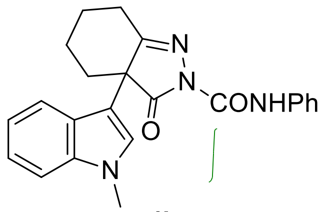
N
N
CONHPh
O
N

4b

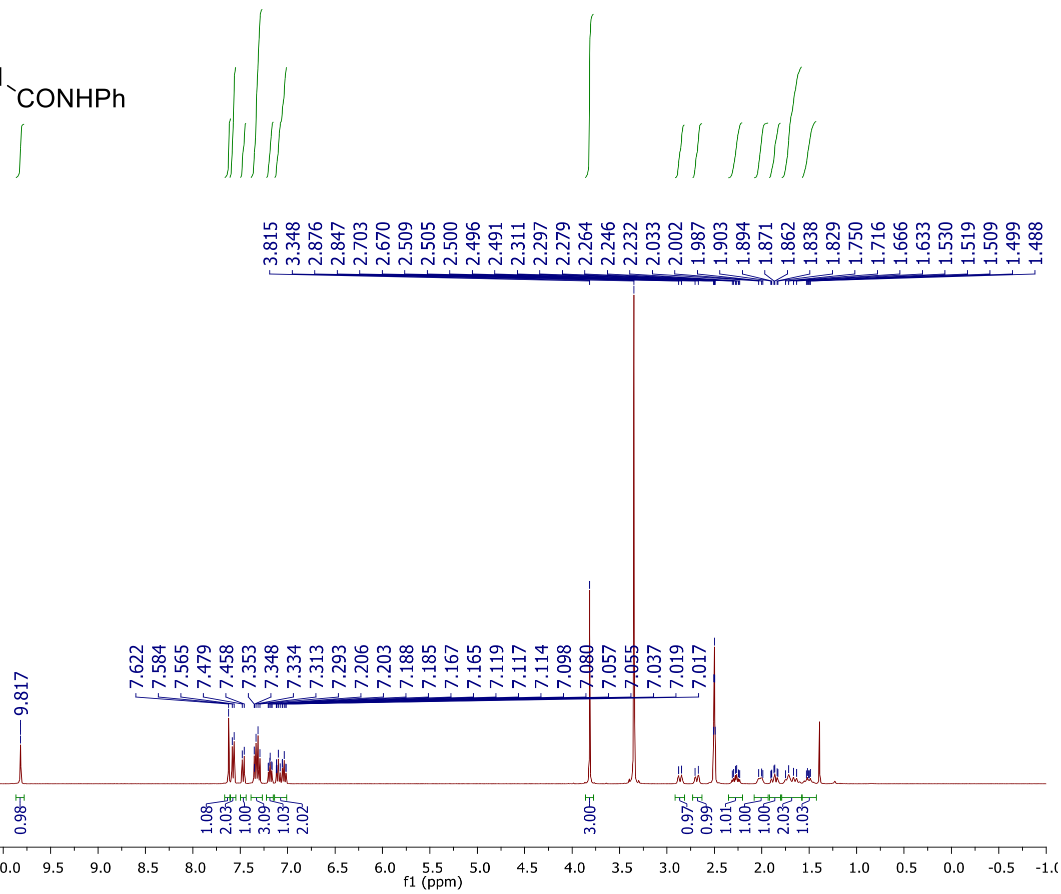
3.815 3.348 2.876 2.847 2.703 2.670 2.509 2.505 2.500 2.496 2.491 2.311 2.297 2.279 2.264 2.246 2.232 2.033 2.002 1.987 1.903 1.894 1.871 1.862 1.838 1.829 1.750 1.716 1.666 1.633 1.530 1.519 1.509 1.499 1.488
9.817
7.622 7.584 7.565 7.479 7.458 7.353 7.348 7.334 7.313 7.293 7.206 7.203 7.188 7.185 7.167 7.165 7.119 7.117 7.114 7.098 7.080 7.057 7.055 7.037 7.019 7.017
0.98 1.08 2.03 1.00 3.09 1.03 2.02 3.00 0.97 0.99 1.01 1.00 1.00 2.03 1.03
11.5 11.0 10.5 10.0 9.5 9.0 8.5 8.0 7.5 7.0 6.5 6.0 5.5 5.0 4.5 4.0 3.5 3.0 2.5 2.0 1.5 1.0 0.5 0.0 -0.5 -1.0
f1 (ppm)

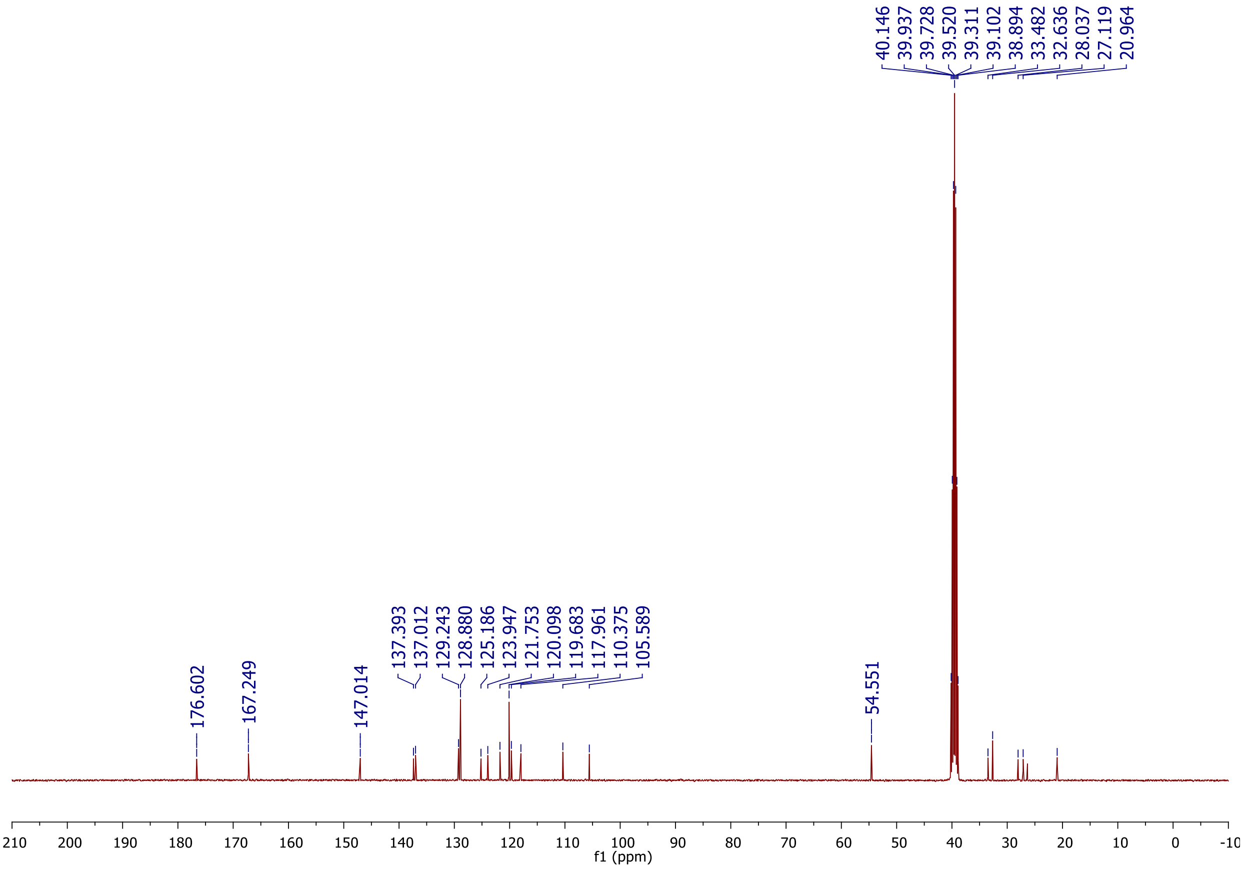
40.146 39.937 39.728 39.520 39.311 39.102 38.894 33.482 32.636 28.037 27.119 20.964
176.602 167.249 147.014
137.393 137.012 129.243 128.880 125.186 123.947 121.753 120.098 119.683 117.961 110.375 105.589
54.551
210 200 190 180 170 160 150 140 130 120 110 100 90 80 70 60 50 40 30 20 10 0 -10
f1 (ppm)

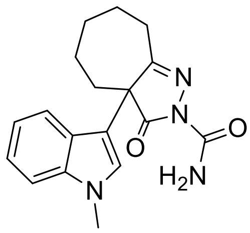
N
N
O
O
H2N
N

4c

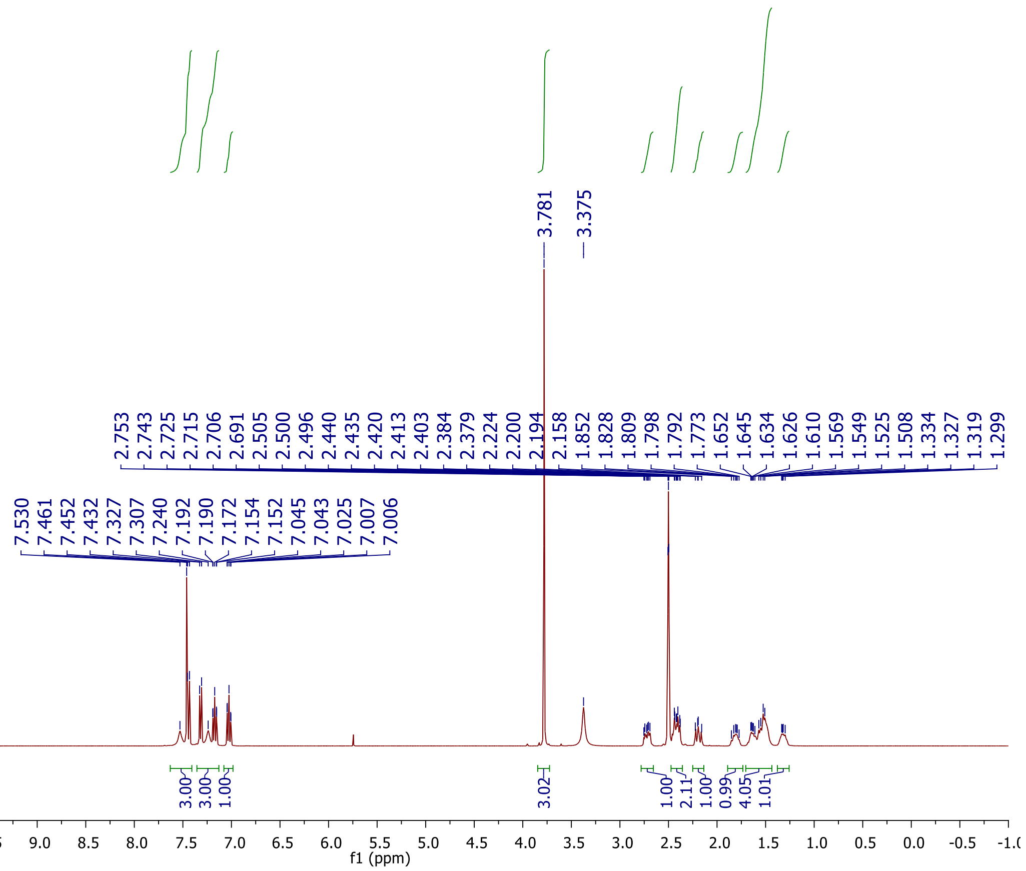
3.781
3.375
2.753 2.743 2.725 2.715 2.706 2.691 2.505 2.500 2.496 2.440 2.435 2.420 2.413 2.403 2.384 2.379 2.224 2.200 2.194 2.158 1.852 1.828 1.809 1.798 1.792 1.773 1.652 1.645 1.634 1.626 1.610 1.569 1.549 1.525 1.508 1.334 1.327 1.319 1.299
7.530 7.461 7.452 7.432 7.327 7.307 7.240 7.192 7.190 7.172 7.154 7.152 7.045 7.043 7.025 7.007 7.006
3.00
3.00
1.00
3.02
1.00
2.11
1.00
0.99
4.05
1.01
12.0 11.5 11.0 10.5 10.0 9.5 9.0 8.5 8.0 7.5 7.0 6.5 6.0 5.5 5.0 4.5 4.0 3.5 3.0 2.5 2.0 1.5 1.0 0.5 0.0 -0.5 -1.0
f1 (ppm)

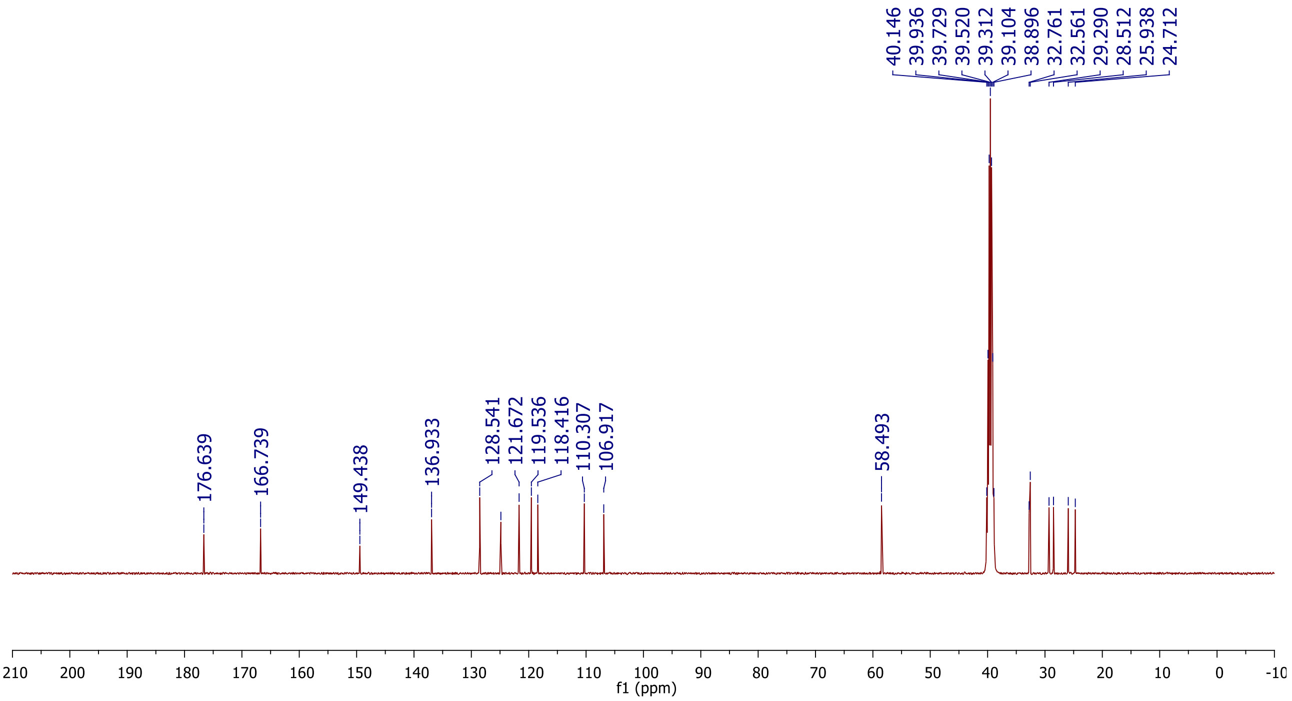
40.146 39.936 39.729 39.520 39.312 39.104 38.896 32.761 32.561 29.290 28.512 25.938 24.712
176.639
166.739
149.438
136.933
128.541
121.672
119.536
118.416
110.307
106.917
58.493
210 200 190 180 170 160 150 140 130 120 110 100 90 80 70 60 50 40 30 20 10 0 -10
f1 (ppm)

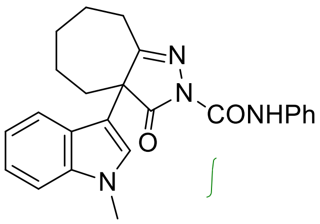
N
N
CONHPh
O
N

4d

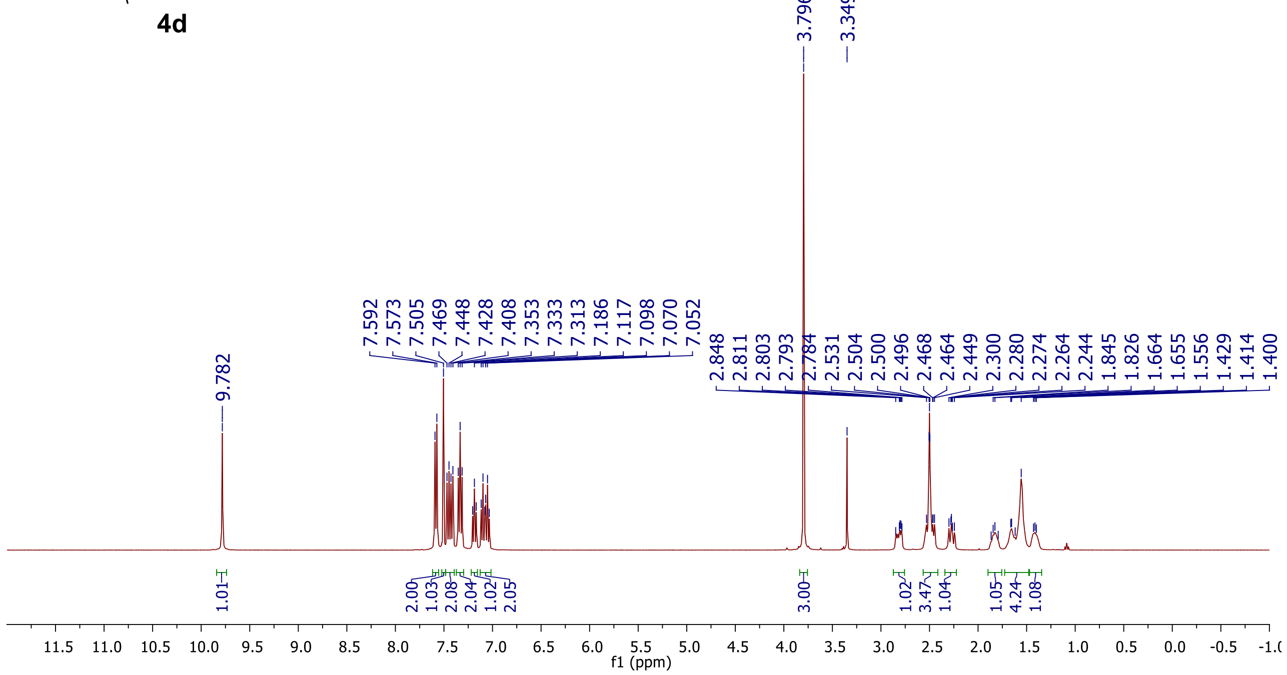
9.782
7.592
7.573
7.505
7.469
7.448
7.428
7.408
7.353
7.333
7.313
7.186
7.117
7.098
7.070
7.052
3.796
3.349
2.848
2.811
2.803
2.793
2.784
2.531
2.504
2.500
2.496
2.468
2.464
2.449
2.300
2.280
2.274
2.264
2.244
1.845
1.826
1.664
1.655
1.556
1.429
1.414
1.400
1.01
2.00
1.03
2.08
2.04
1.02
2.05
3.00
1.02
3.47
1.04
1.05
4.24
1.08
f1 (ppm)

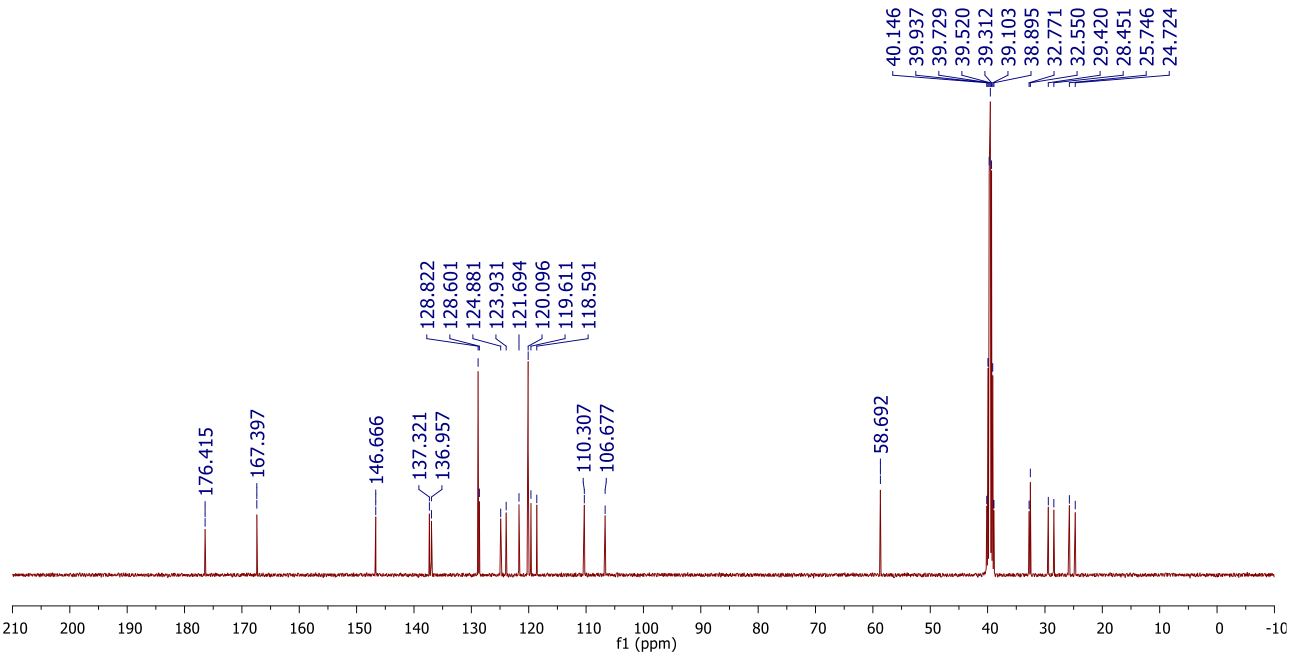
176.415
167.397
146.666
137.321
136.957
128.822
128.601
124.881
123.931
121.694
120.096
119.611
118.591
110.307
106.677
58.692
40.146
39.937
39.729
39.520
39.312
39.103
38.895
32.771
32.550
29.420
28.451
25.746
24.724
f1 (ppm)

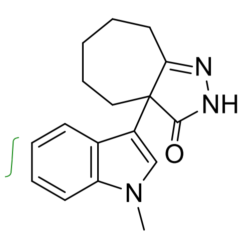
N
NH
O
N

4e

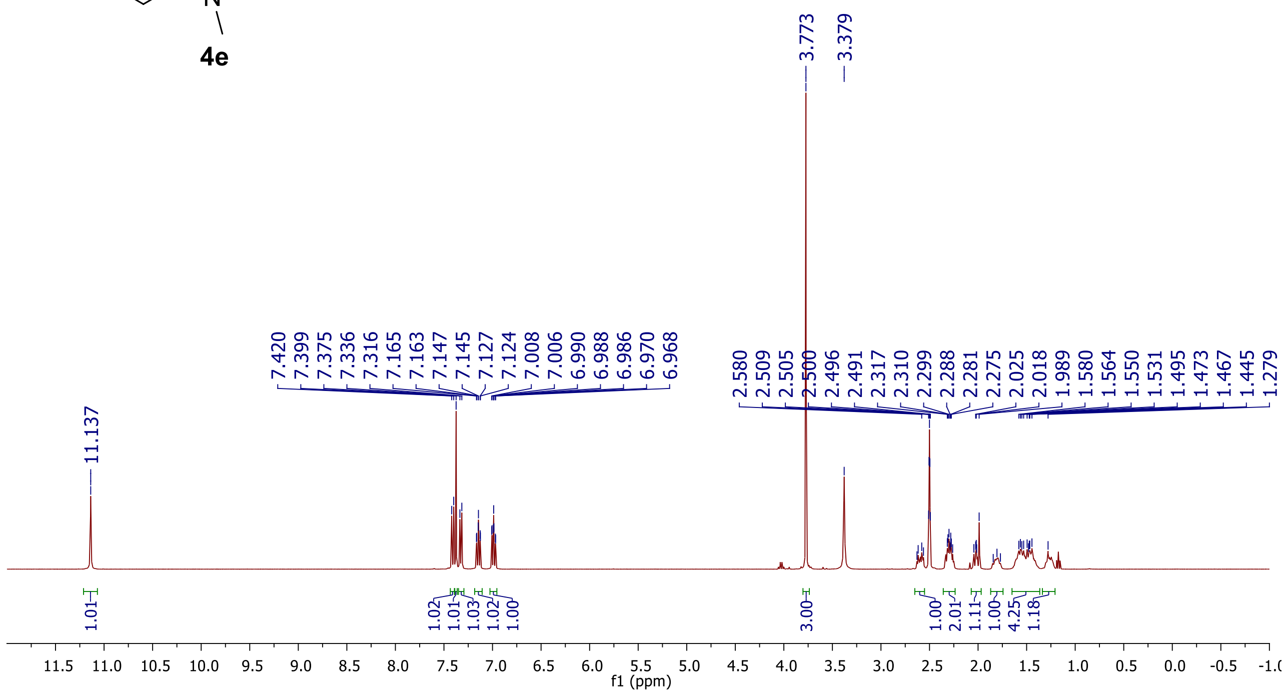
11.137
7.420 7.399 7.375 7.336 7.316 7.165 7.163 7.147 7.145 7.127 7.124 7.008 7.006 6.990 6.988 6.986 6.970 6.968
3.773
3.379
2.580 2.509 2.505 2.500 2.496 2.491 2.317 2.310 2.299 2.288 2.281 2.275 2.025 2.018 1.989 1.580 1.564 1.550 1.531 1.495 1.473 1.467 1.445 1.279
1.01
1.02 1.01 1.03 1.02 1.00
3.00
1.00 2.01 1.11 1.00 4.25 1.18
11.5 11.0 10.5 10.0 9.5 9.0 8.5 8.0 7.5 7.0 6.5 6.0 5.5 5.0 4.5 4.0 3.5 3.0 2.5 2.0 1.5 1.0 0.5 0.0 -0.5
f1 (ppm)

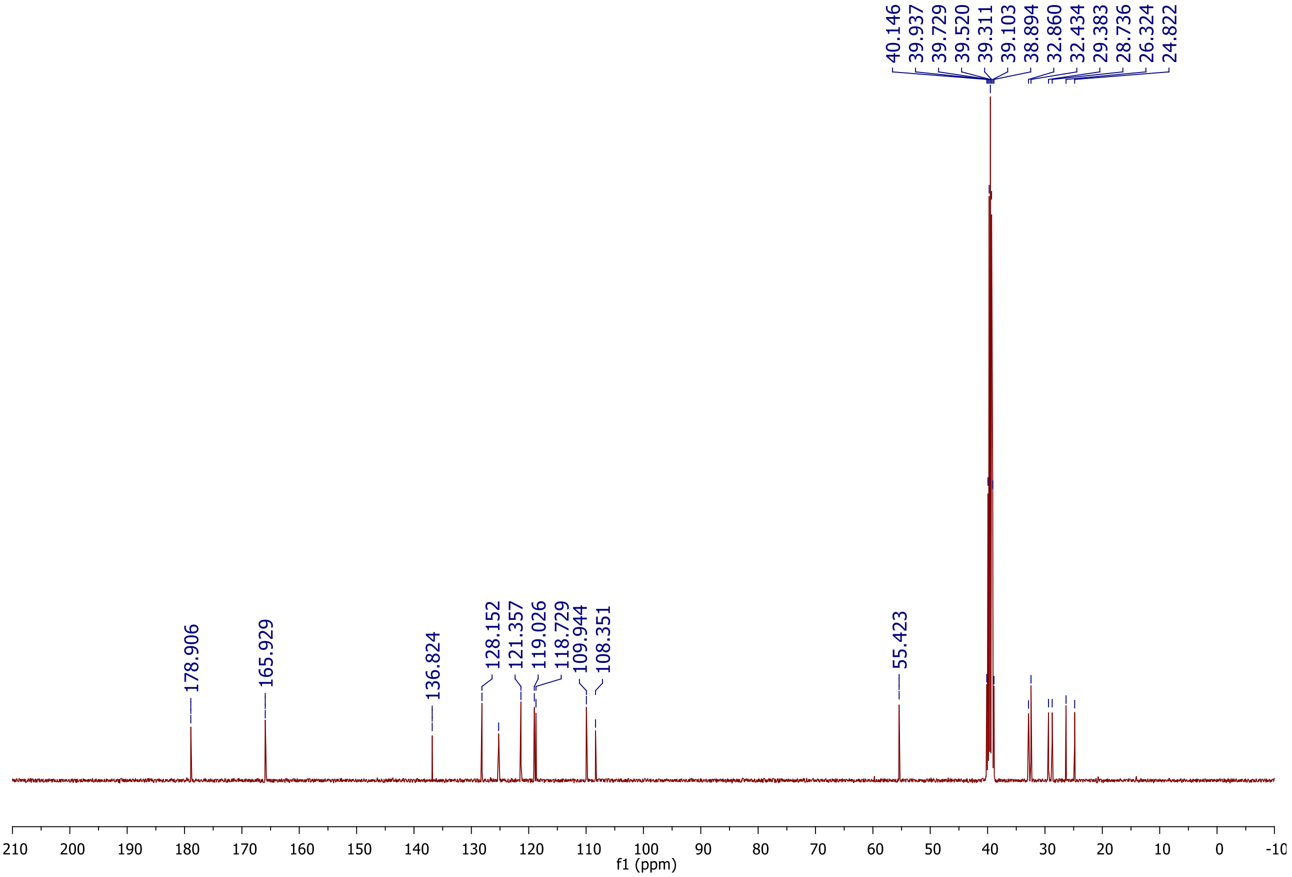
40.146 39.937 39.729 39.520 39.311 39.103 38.894 32.860 32.434 29.383 28.736 26.324 24.822
178.906
165.929
136.824
128.152 121.357 119.026 118.729 109.944 108.351
55.423
210 200 190 180 170 160 150 140 130 120 110 100 90 80 70 60 50 40 30 20 10 0 -10
f1 (ppm)

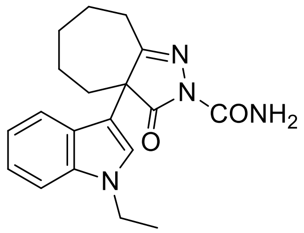

4f

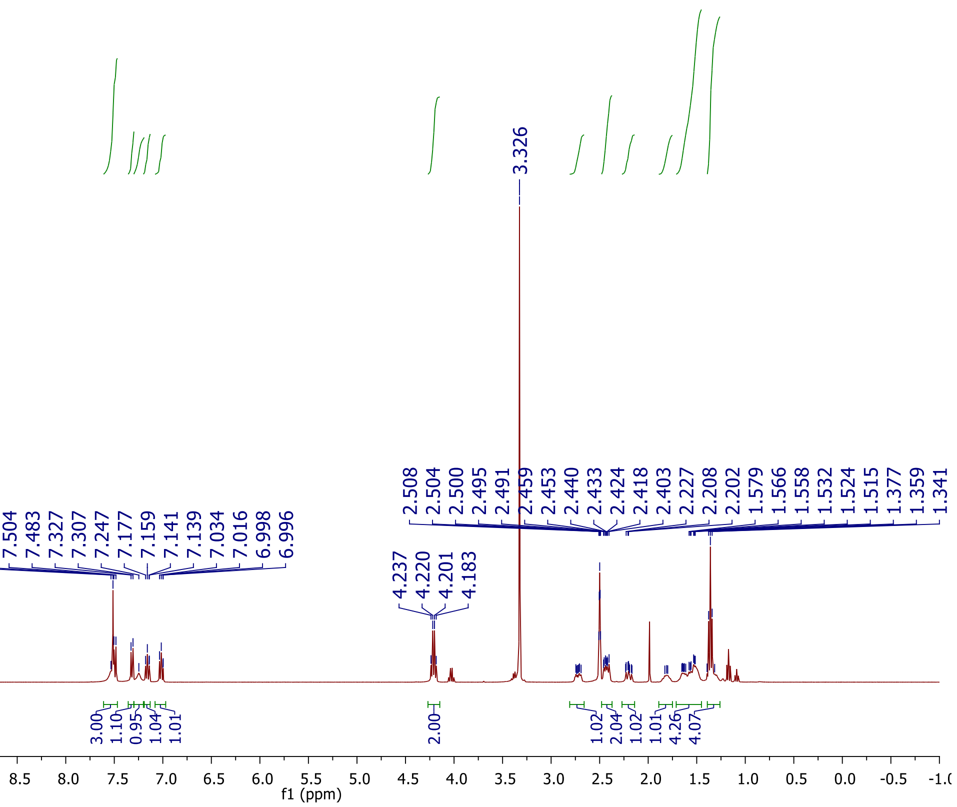

7.534
7.513
7.504
7.483
7.327
7.307
7.247
7.177
7.159
7.141
7.139
7.034
7.016
6.998
6.996
4.237
4.220
4.201
4.183
3.326
2.508
2.504
2.500
2.495
2.491
2.459
2.453
2.440
2.433
2.424
2.418
2.403
2.227
2.208
2.202
1.579
1.566
1.558
1.532
1.524
1.515
1.377
1.359
1.341
3.00
1.10
0.95
1.04
1.01
2.00
1.02
2.04
1.02
1.01
4.26
4.07
f1 (ppm)

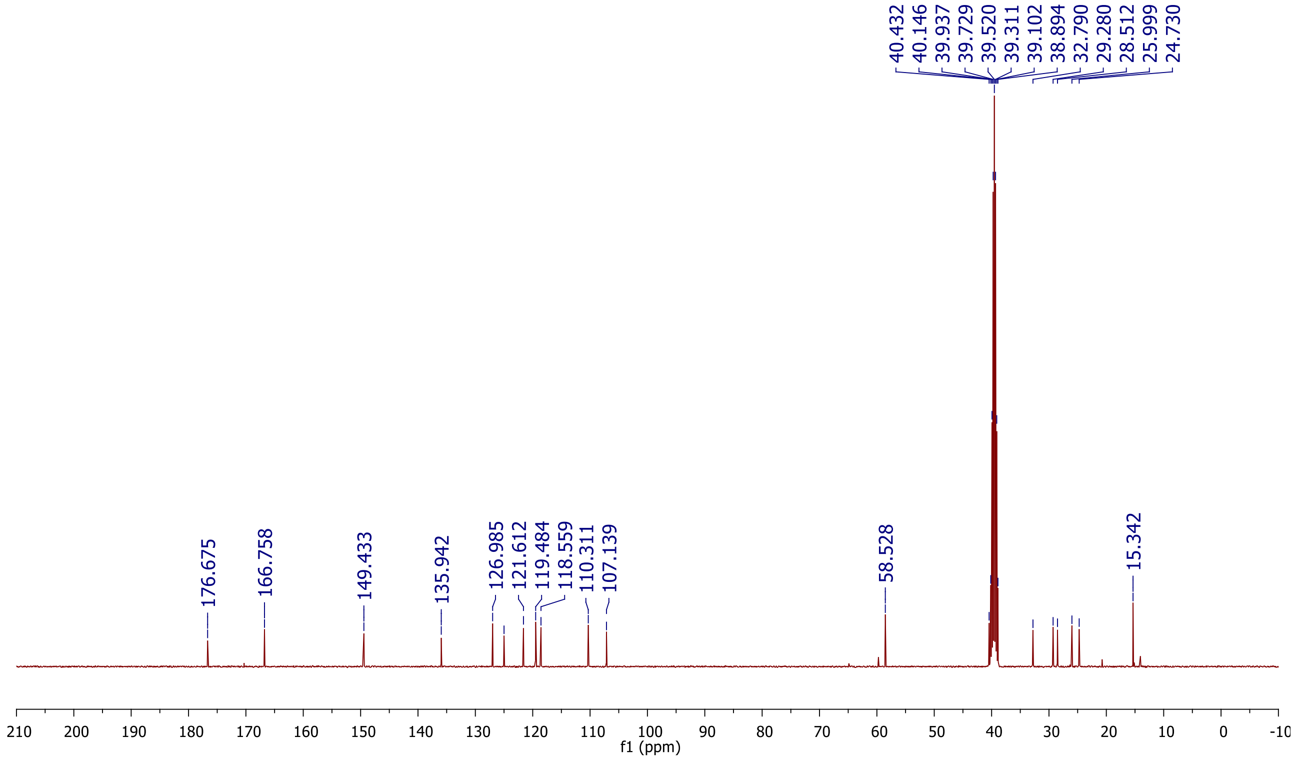

176.675
166.758
149.433
135.942
126.985
121.612
119.484
118.559
110.311
107.139
58.528
40.432
40.146
39.937
39.729
39.520
39.311
39.102
38.894
32.790
29.280
28.512
25.999
24.730
15.342
f1 (ppm)

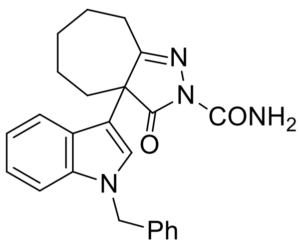
N
N
CONH2
O
N
Ph

4g

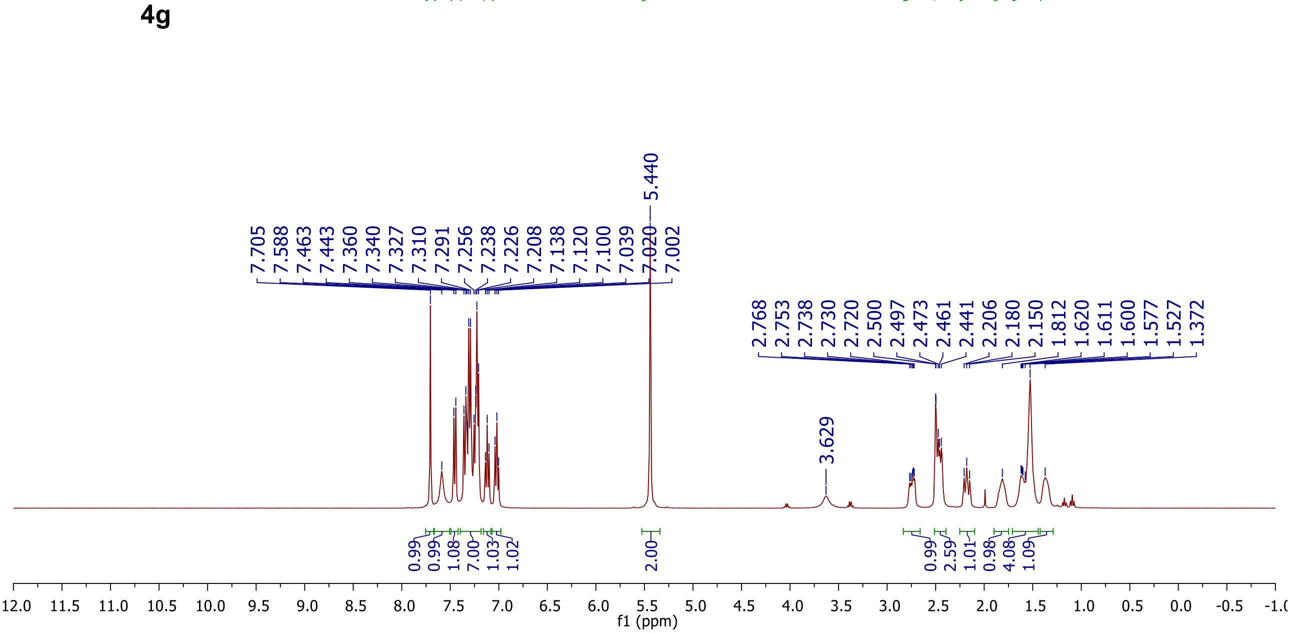
7.705
7.588
7.463
7.443
7.360
7.340
7.327
7.310
7.291
7.256
7.238
7.226
7.208
7.138
7.120
7.100
7.039
7.020
7.002
5.440
2.768
2.753
2.738
2.730
2.720
2.500
2.497
2.473
2.461
2.441
2.206
2.180
2.150
1.812
1.620
1.611
1.600
1.577
1.527
1.372
3.629
0.99
0.99
1.08
7.00
1.03
1.02
2.00
0.99
2.59
1.01
0.98
4.08
1.09
f1 (ppm)

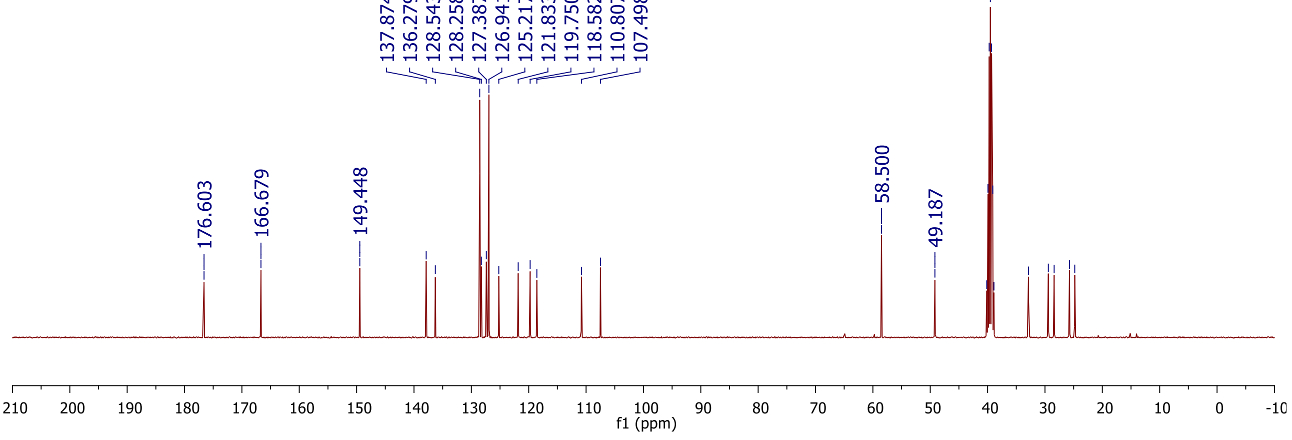
176.603
166.679
149.448
137.874
136.279
128.543
128.258
127.387
126.941
125.217
121.833
119.750
118.582
110.807
107.498
58.500
49.187
40.146
39.937
39.729
39.520
39.311
39.103
38.894
32.873
29.411
28.399
25.724
24.808
f1 (ppm)

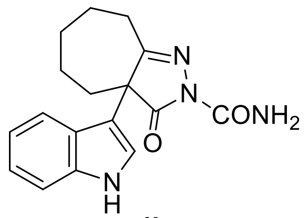
N
N
CONH2
O
N
H

4h

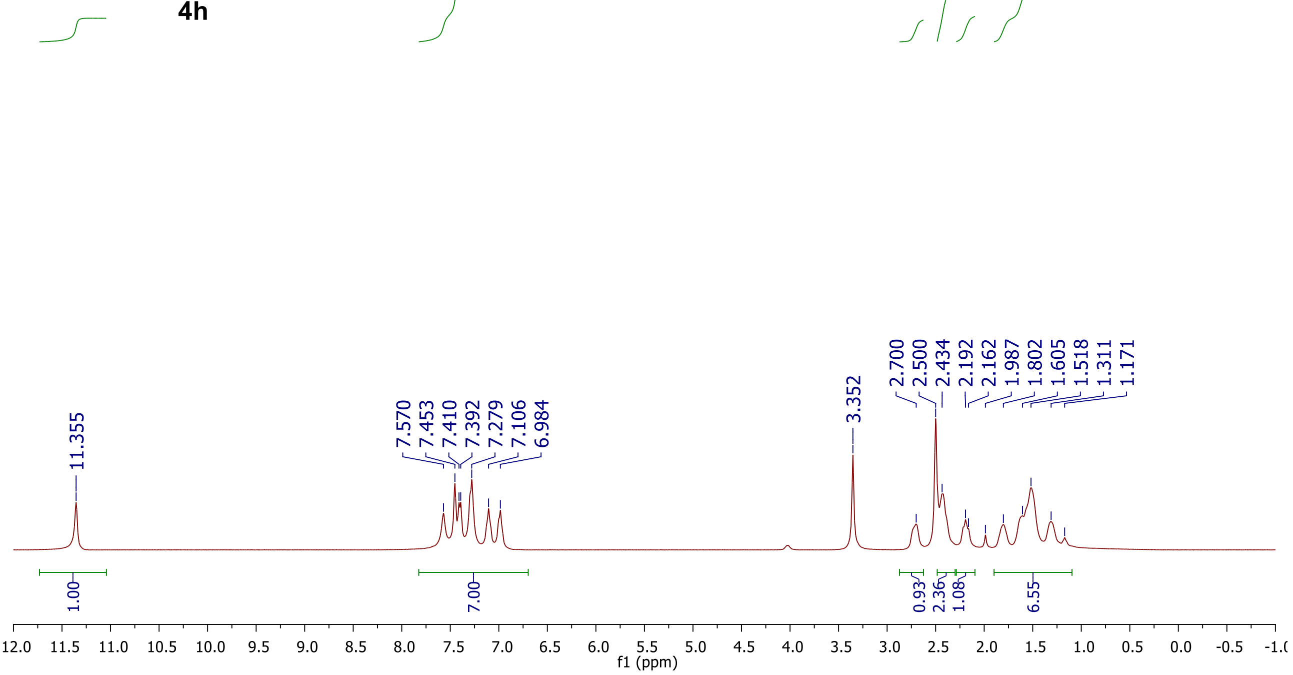
11.355
7.570
7.453
7.410
7.392
7.279
7.106
6.984
3.352
2.700
2.500
2.434
2.192
2.162
1.987
1.802
1.605
1.518
1.311
1.171
1.00
7.00
0.93
2.36
1.08
6.55
12.0 11.5 11.0 10.5 10.0 9.5 9.0 8.5 8.0 7.5 7.0 6.5 6.0 5.5 5.0 4.5 4.0 3.5 3.0 2.5 2.0 1.5 1.0 0.5 0.0 -0.5
f1 (ppm)

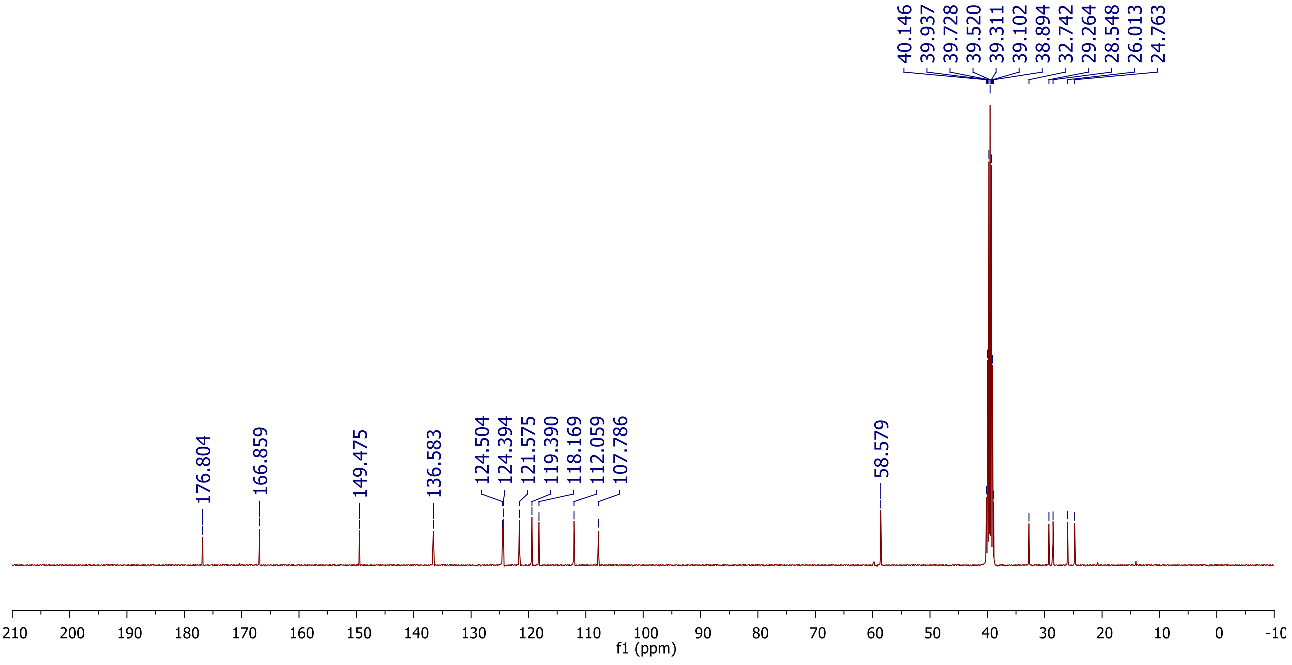
176.804
166.859
149.475
136.583
124.504
124.394
121.575
119.390
118.169
112.059
107.786
58.579
40.146
39.937
39.728
39.520
39.311
39.102
38.894
32.742
29.264
28.548
26.013
24.763
210 200 190 180 170 160 150 140 130 120 110 100 90 80 70 60 50 40 30 20 10 0
f1 (ppm)

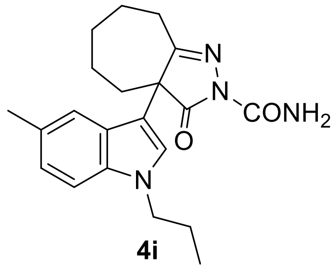
N
N
CONH2
O
N

4i

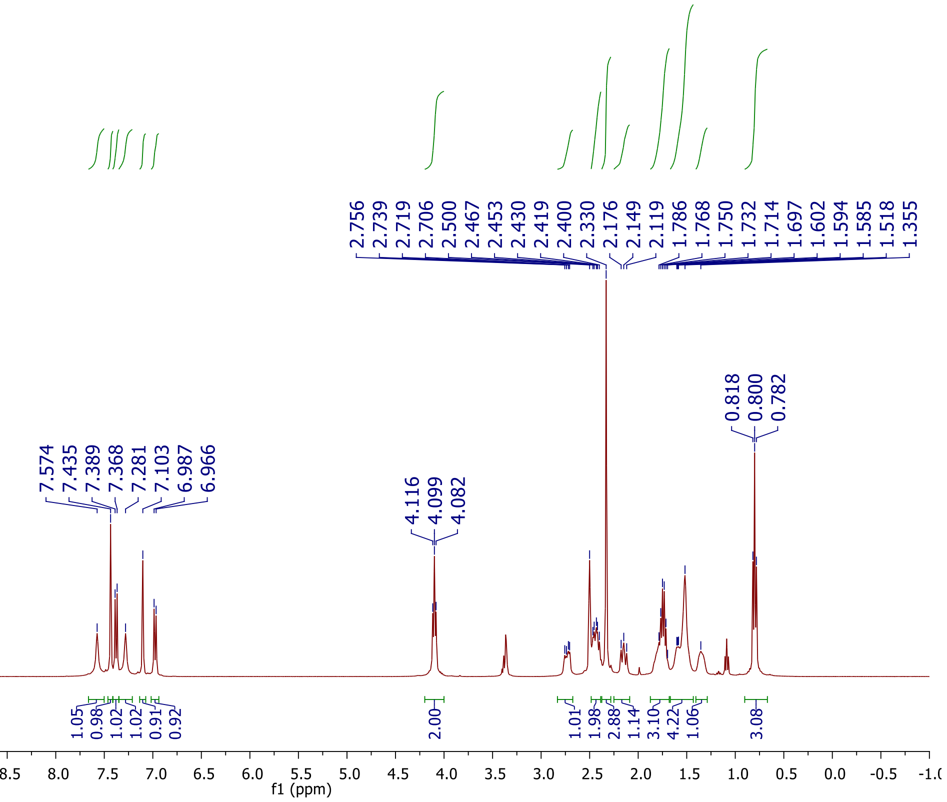
7.574
7.435
7.389
7.368
7.281
7.103
6.987
6.966
4.116
4.099
4.082
2.756
2.739
2.719
2.706
2.500
2.467
2.453
2.430
2.419
2.400
2.330
2.176
2.149
2.119
1.786
1.768
1.750
1.732
1.714
1.697
1.602
1.594
1.585
1.518
1.355
0.818
0.800
0.782
1.05
0.98
1.02
1.02
0.91
0.92
2.00
1.01
1.98
2.88
1.14
3.10
4.22
1.06
3.08
12.0 11.5 11.0 10.5 10.0 9.5 9.0 8.5 8.0 7.5 7.0 6.5 6.0 5.5 5.0 4.5 4.0 3.5 3.0 2.5 2.0 1.5 1.0 0.5 0.0 -0.5
f1 (ppm)

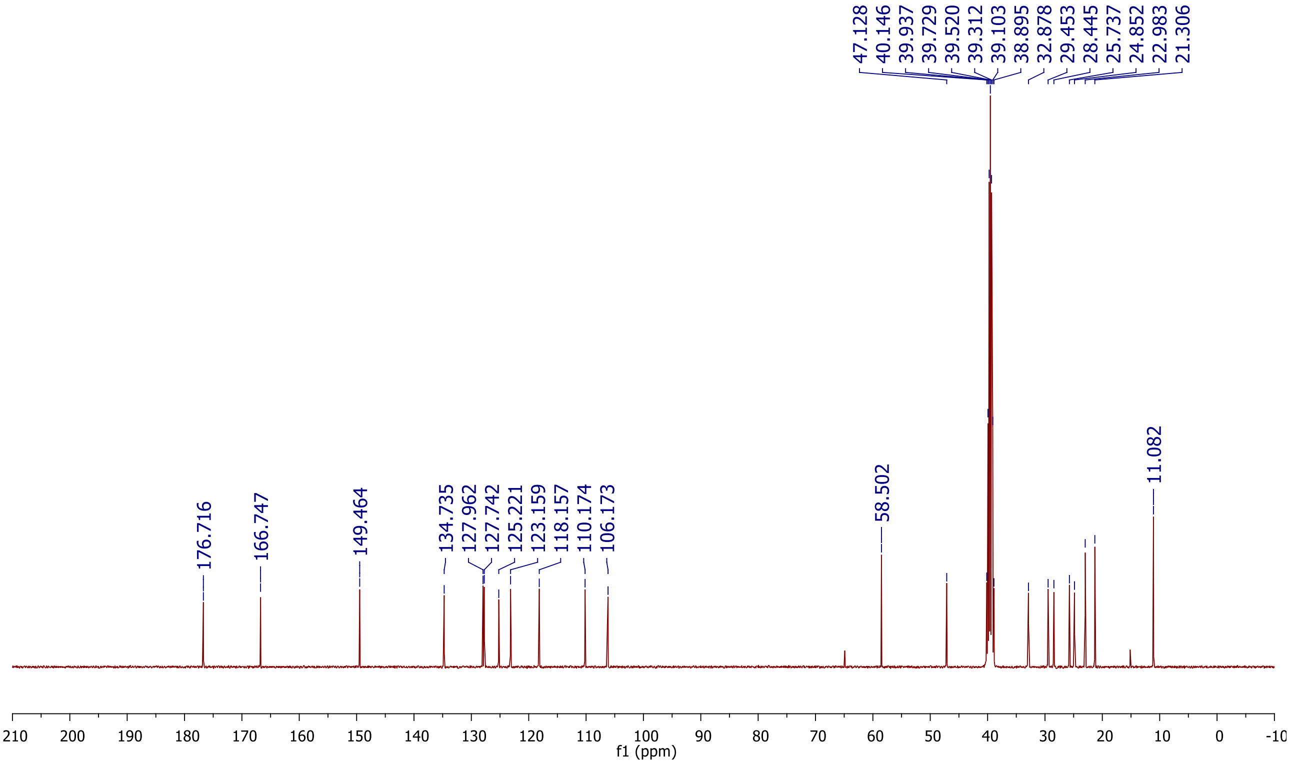
176.716
166.747
149.464
134.735
127.962
127.742
125.221
123.159
118.157
110.174
106.173
58.502
47.128
40.146
39.937
39.729
39.520
39.312
39.103
38.895
32.878
29.453
28.445
25.737
24.852
22.983
21.306
11.082
210 200 190 180 170 160 150 140 130 120 110 100 90 80 70 60 50 40 30 20 10 0 -10
f1 (ppm)

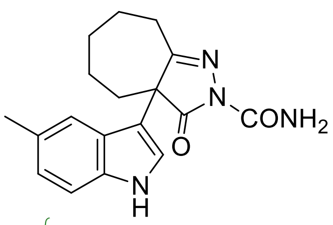
N
N
CONH2
O
N
H

4j

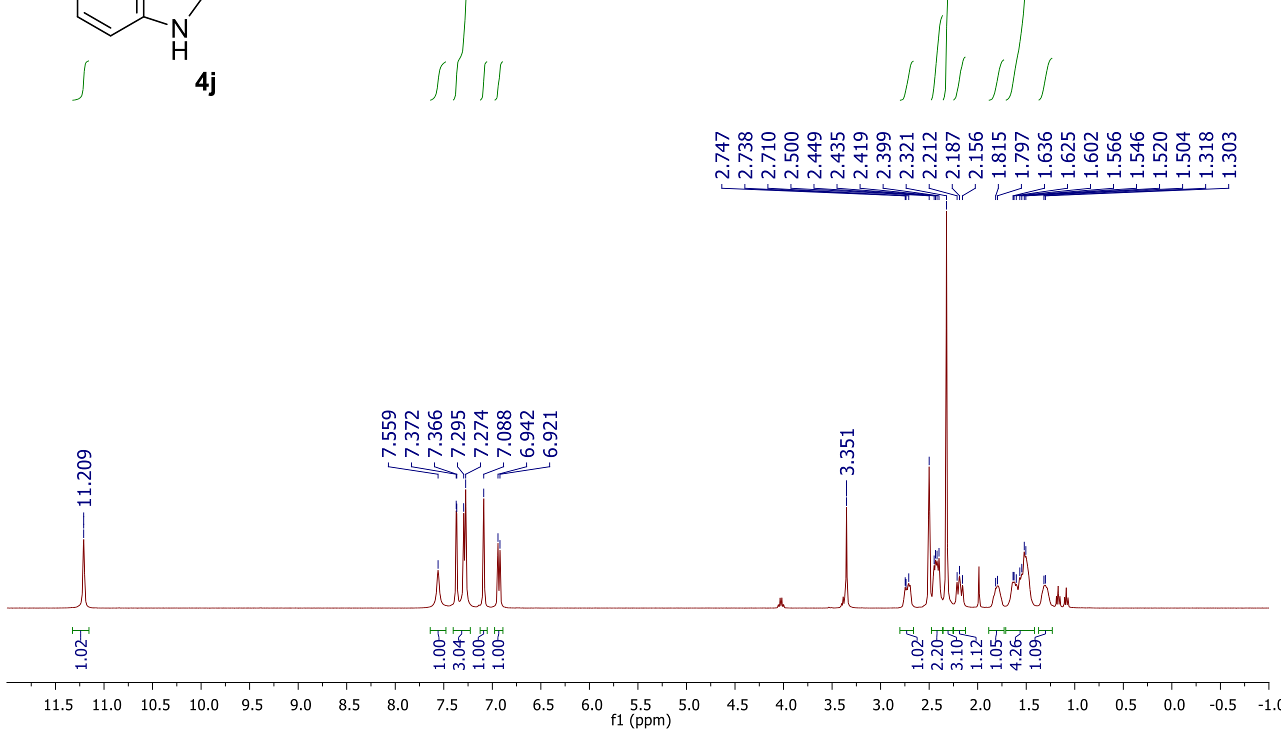
2.747
2.738
2.710
2.500
2.449
2.435
2.419
2.399
2.321
2.212
2.187
2.156
1.815
1.797
1.636
1.625
1.602
1.566
1.546
1.520
1.504
1.318
1.303
11.209
7.559
7.372
7.366
7.295
7.274
7.088
6.942
6.921
3.351
1.02
1.00
3.04
1.00
1.00
1.02
2.20
3.10
1.12
1.05
4.26
1.09
f1 (ppm)

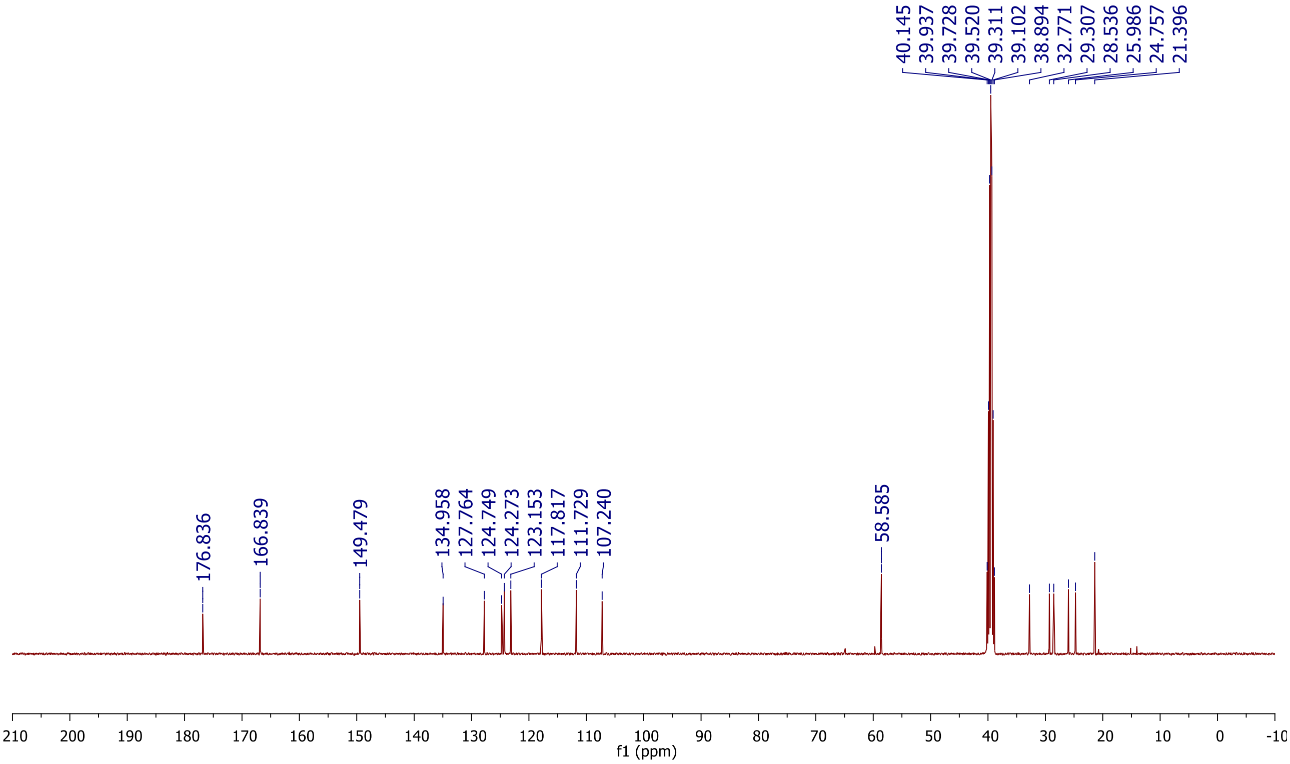
40.145
39.937
39.728
39.520
39.311
39.102
38.894
32.771
29.307
28.536
25.986
24.757
21.396
176.836
166.839
149.479
134.958
127.764
124.749
124.273
123.153
117.817
111.729
107.240
58.585
f1 (ppm)

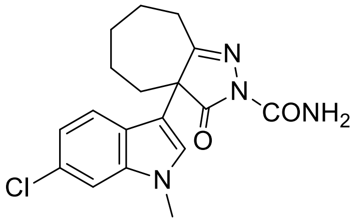
N
N
CONH2
O
Cl
N

4k

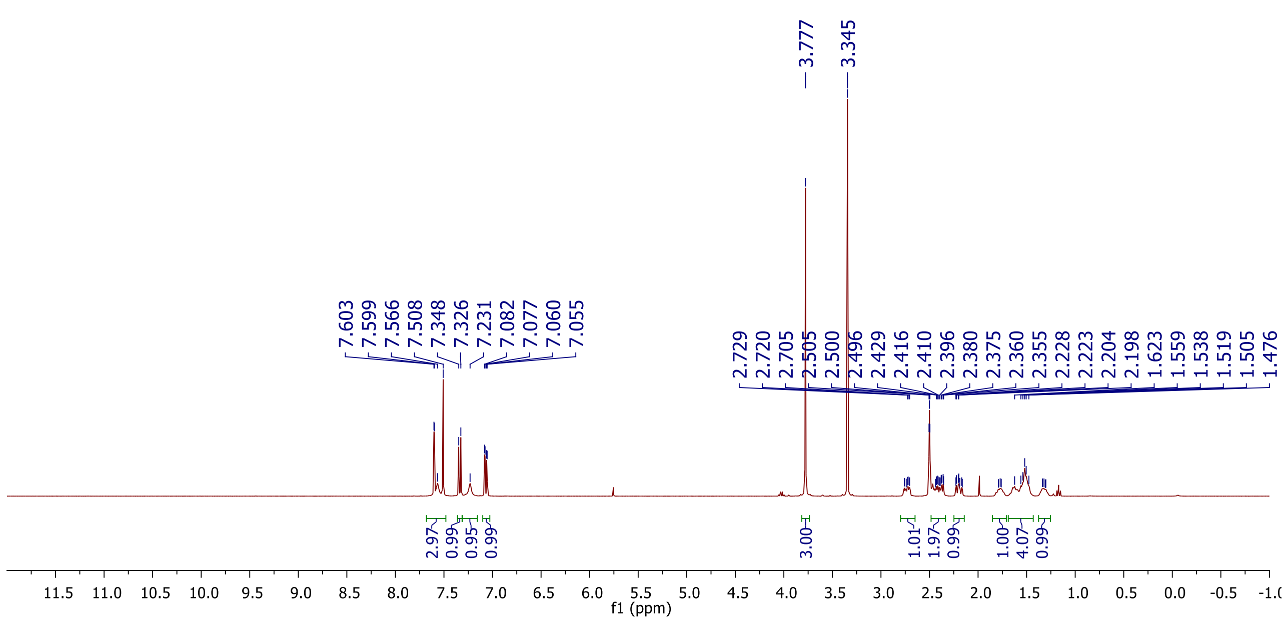
3.777
3.345
7.603
7.599
7.566
7.508
7.348
7.326
7.231
7.082
7.077
7.060
7.055
2.729
2.720
2.705
2.505
2.500
2.496
2.429
2.416
2.410
2.396
2.380
2.375
2.360
2.355
2.228
2.223
2.204
2.198
1.623
1.559
1.538
1.519
1.505
1.476
2.97
0.99
0.95
0.99
3.00
1.01
1.97
0.99
1.00
4.07
0.99
11.5 11.0 10.5 10.0 9.5 9.0 8.5 8.0 7.5 7.0 6.5 6.0 5.5 5.0 4.5 4.0 3.5 3.0 2.5 2.0 1.5 1.0 0.5 0.0 -0.5 -1.0
f1 (ppm)

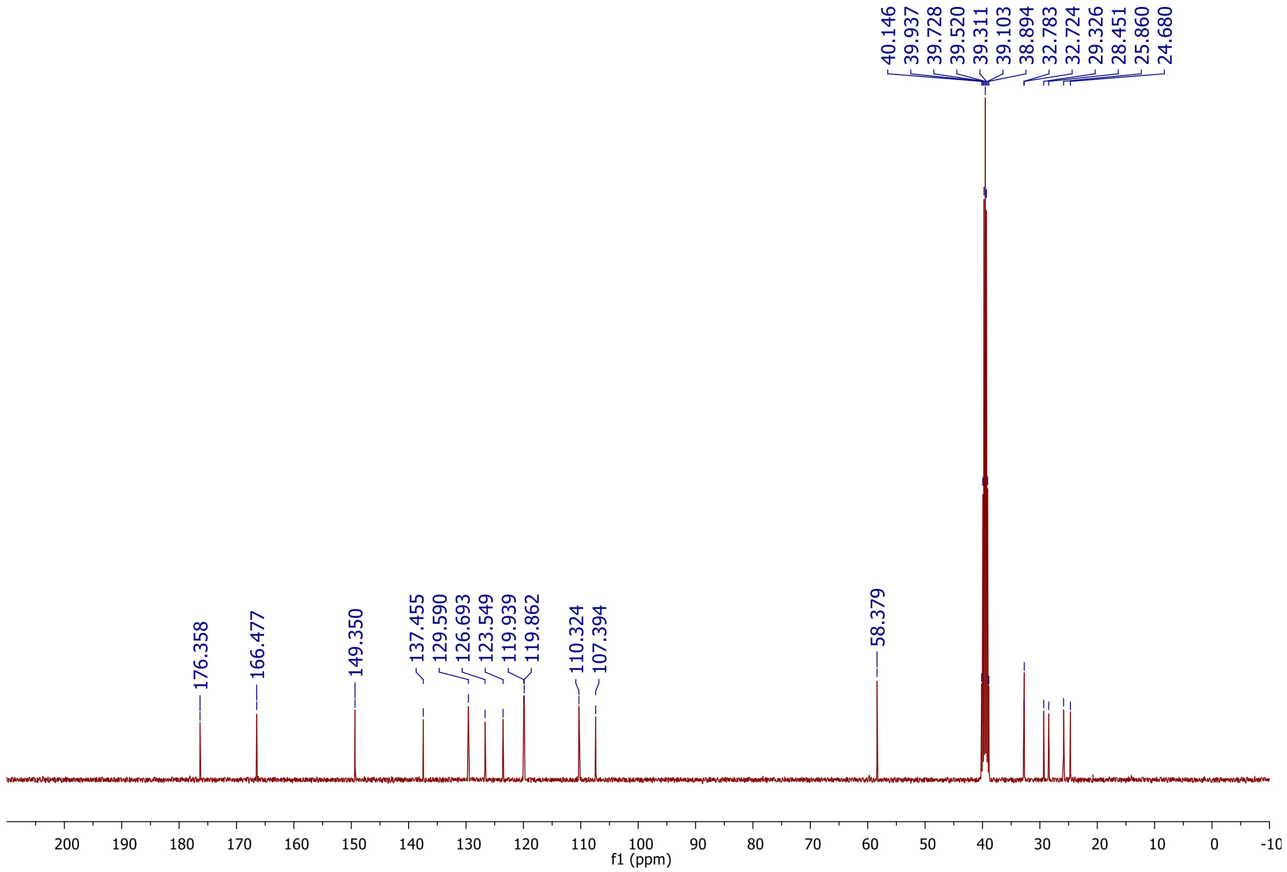
40.146
39.937
39.728
39.520
39.311
39.103
38.894
32.783
32.724
29.326
28.451
25.860
24.680
176.358
166.477
149.350
137.455
129.590
126.693
123.549
119.939
119.862
110.324
107.394
58.379
200 190 180 170 160 150 140 130 120 110 100 90 80 70 60 50 40 30 20 10 0 -10
f1 (ppm)

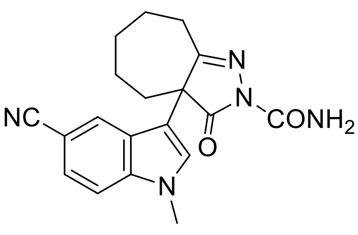
NC
N
N
CONH2
O
N

4l

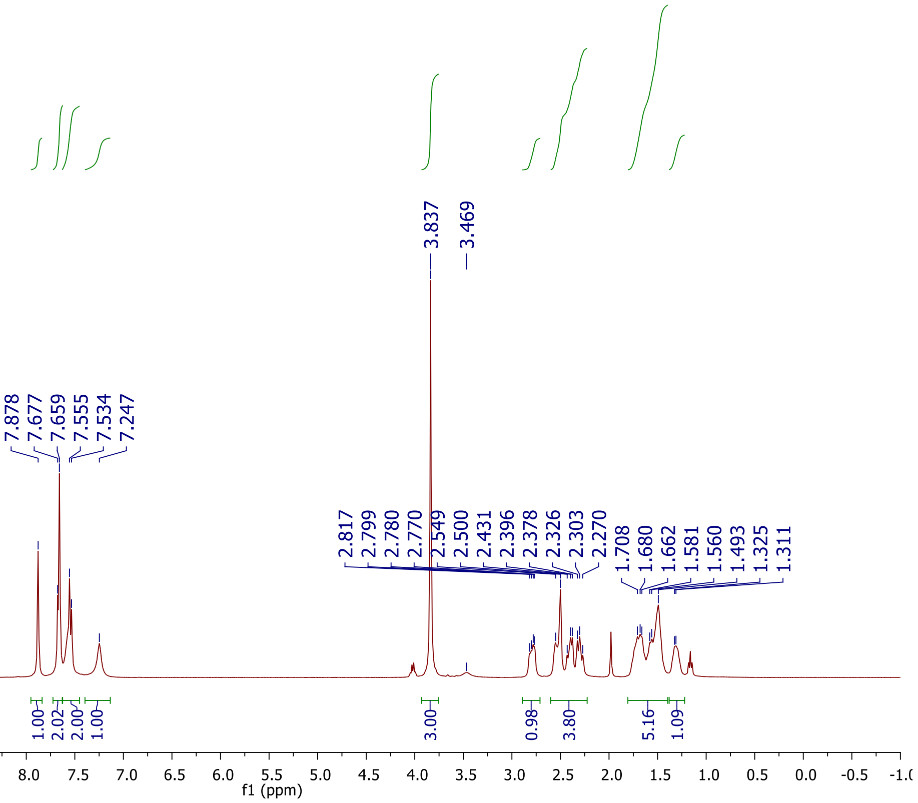
7.878
7.677
7.659
7.555
7.534
7.247
3.837
3.469
2.817
2.799
2.780
2.770
2.549
2.500
2.431
2.396
2.378
2.326
2.303
2.270
1.708
1.680
1.662
1.581
1.560
1.493
1.325
1.311
1.00
2.02
2.00
1.00
3.00
0.98
3.80
5.16
1.09
11.5 11.0 10.5 10.0 9.5 9.0 8.5 8.0 7.5 7.0 6.5 6.0 5.5 5.0 4.5 4.0 3.5 3.0 2.5 2.0 1.5 1.0 0.5 0.0 -0.5
f1 (ppm)

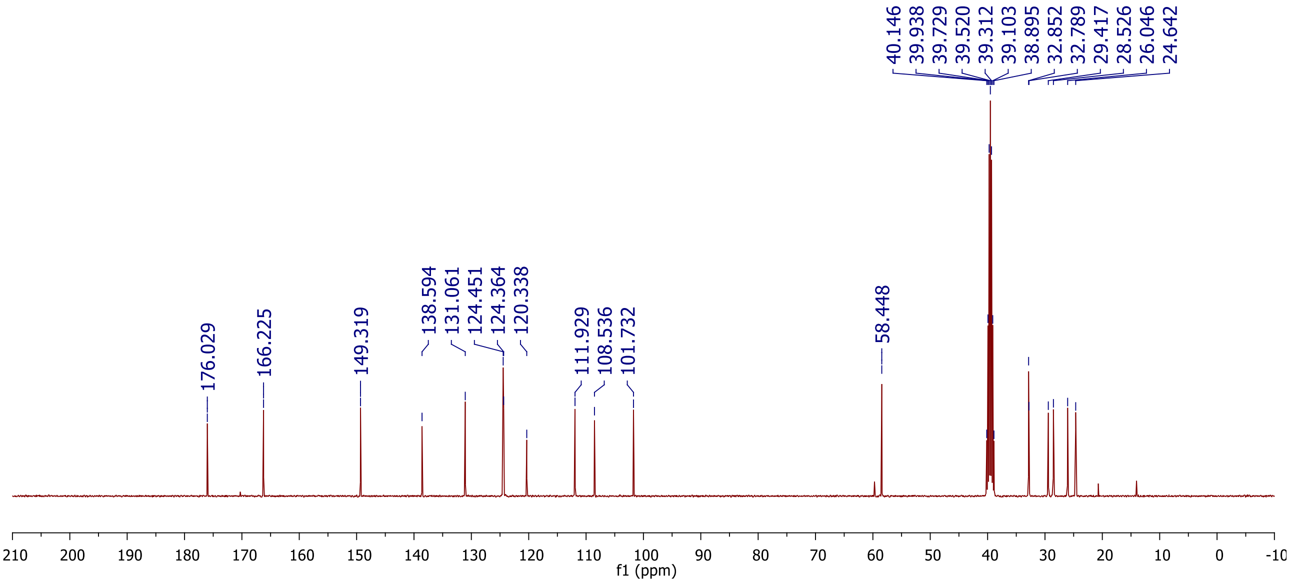
176.029
166.225
149.319
138.594
131.061
124.451
124.364
120.338
111.929
108.536
101.732
58.448
40.146
39.938
39.729
39.520
39.312
39.103
38.895
32.852
32.789
29.417
28.526
26.046
24.642
210 200 190 180 170 160 150 140 130 120 110 100 90 80 70 60 50 40 30 20 10 0 -10
f1 (ppm)

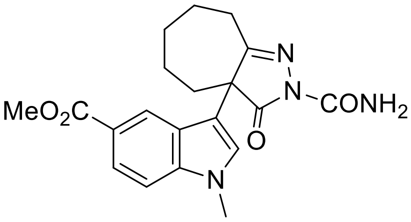
MeO2C
N
N
CONH2
O
N

4m

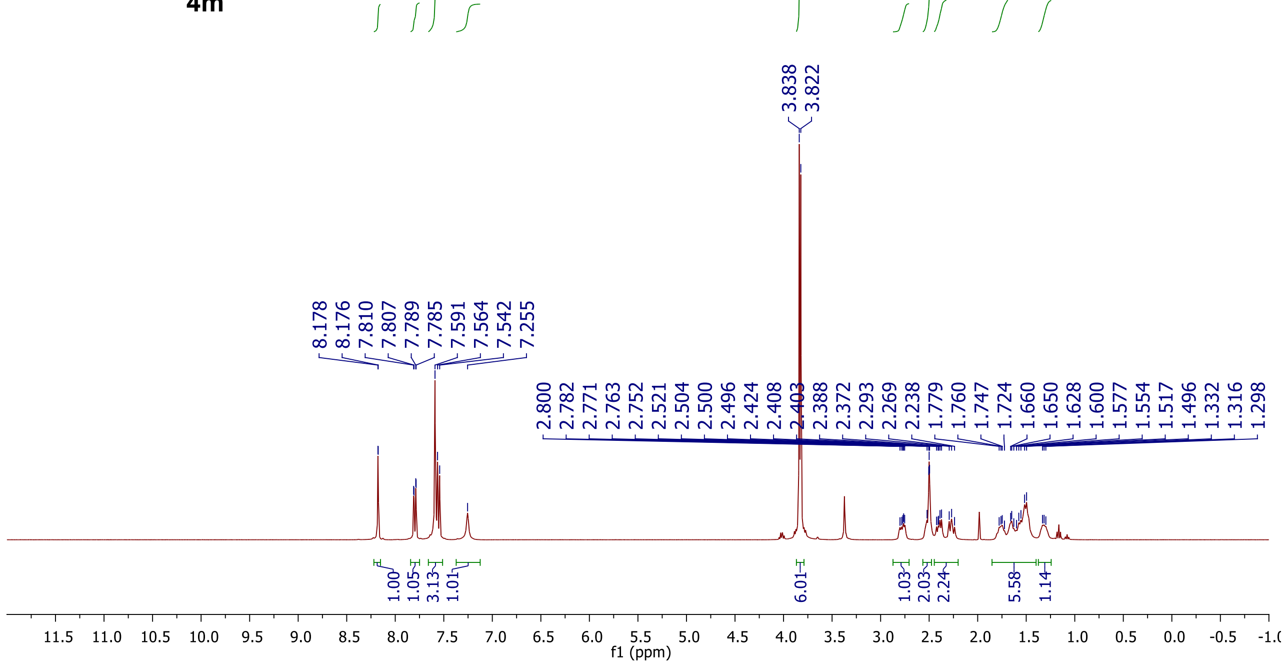
3.838
3.822
8.178
8.176
7.810
7.807
7.789
7.785
7.591
7.564
7.542
7.255
2.800
2.782
2.771
2.763
2.752
2.521
2.504
2.500
2.496
2.424
2.408
2.403
2.388
2.372
2.293
2.269
2.238
1.779
1.760
1.747
1.724
1.660
1.650
1.628
1.600
1.577
1.554
1.517
1.496
1.332
1.316
1.298
1.00
1.05
3.13
1.01
6.01
1.03
2.03
2.24
5.58
1.14
f1 (ppm)

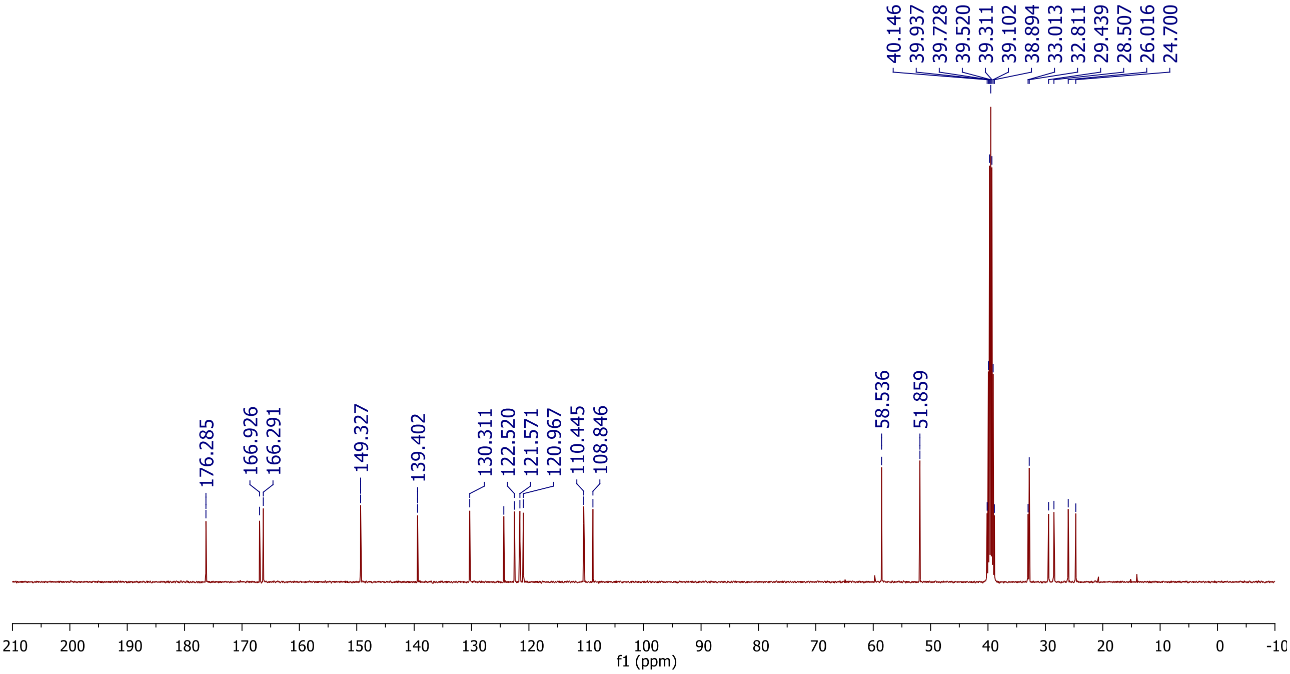
40.146
39.937
39.728
39.520
39.311
39.102
38.894
33.013
32.811
29.439
28.507
26.016
24.700
176.285
166.926
166.291
149.327
139.402
130.311
122.520
121.571
120.967
110.445
108.846
58.536
51.859
f1 (ppm)

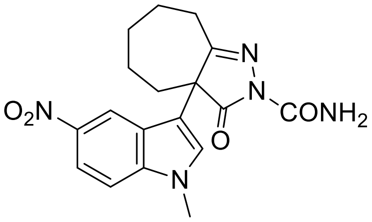
O2N
N
N
CONH2
O
N

4n

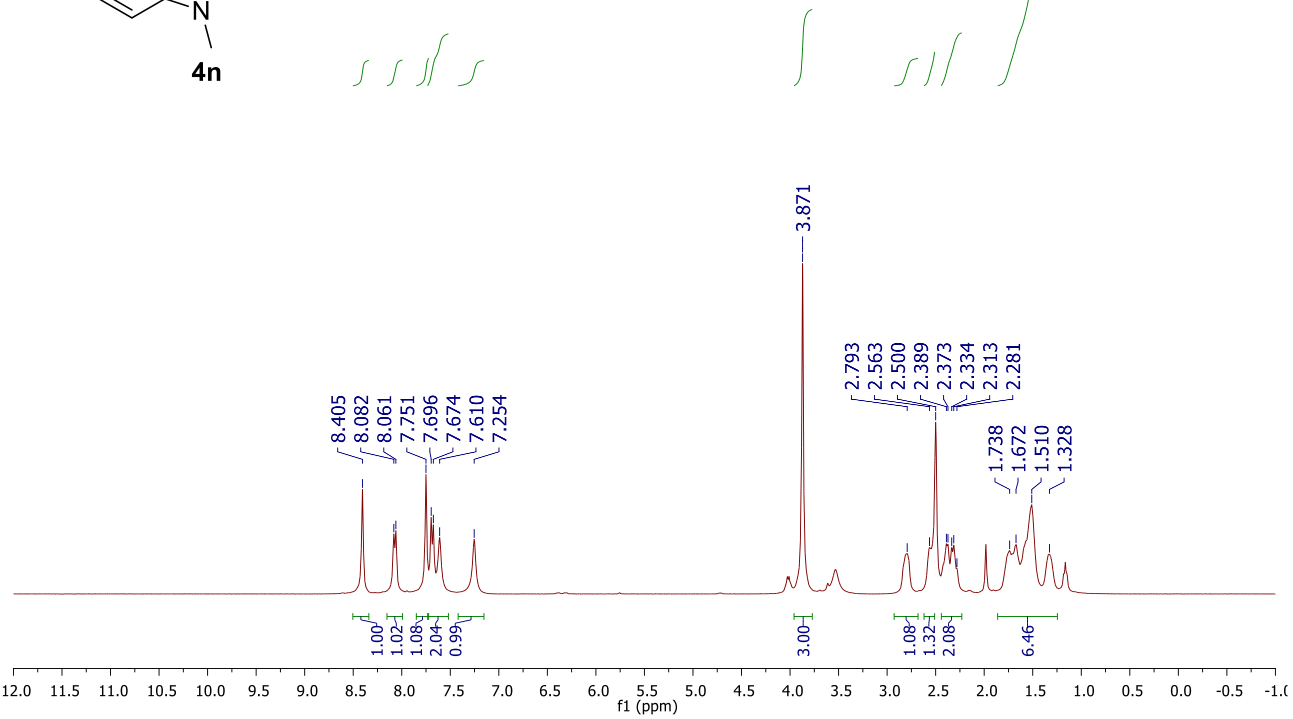
8.405
8.082
8.061
7.751
7.696
7.674
7.610
7.254
3.871
2.793
2.563
2.500
2.389
2.373
2.334
2.313
2.281
1.738
1.672
1.510
1.328
1.00
1.02
1.08
2.04
0.99
3.00
1.08
1.32
2.08
6.46
12.0 11.5 11.0 10.5 10.0 9.5 9.0 8.5 8.0 7.5 7.0 6.5 6.0 5.5 5.0 4.5 4.0 3.5 3.0 2.5 2.0 1.5 1.0 0.5 0.0 -0.5
f1 (ppm)

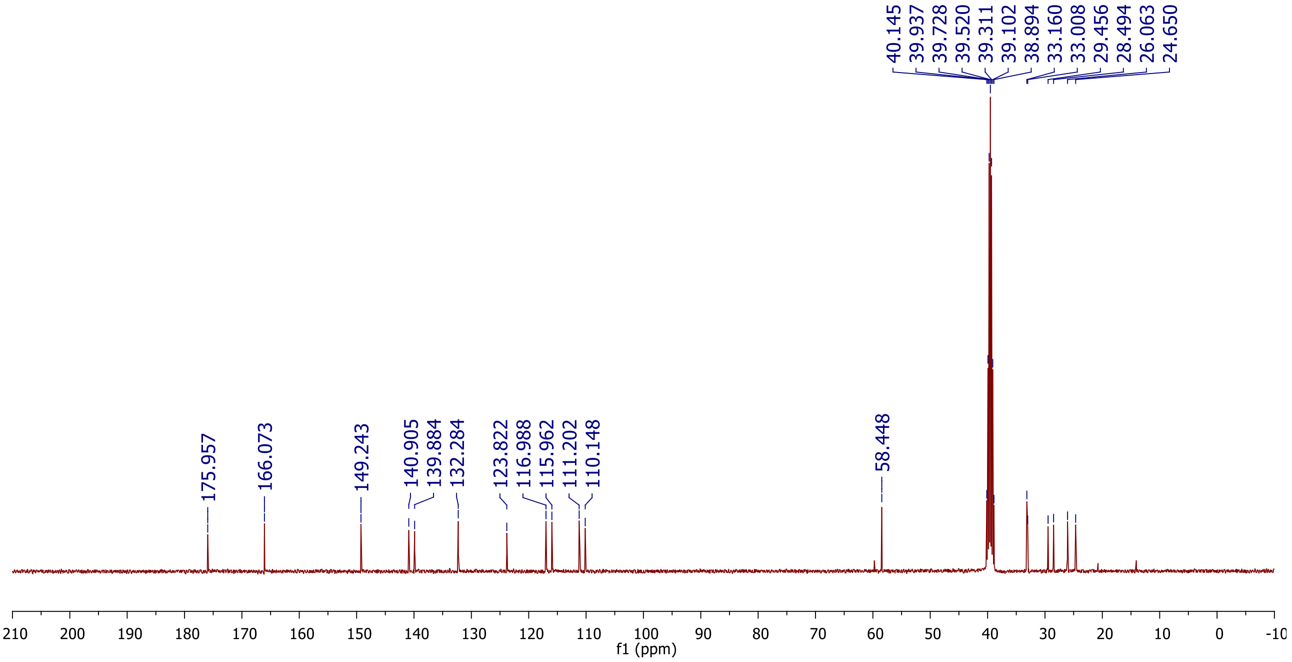
175.957
166.073
149.243
140.905
139.884
132.284
123.822
116.988
115.962
111.202
110.148
58.448
40.145
39.937
39.728
39.520
39.311
39.102
38.894
33.160
33.008
29.456
28.494
26.063
24.650
210 200 190 180 170 160 150 140 130 120 110 100 90 80 70 60 50 40 30 20 10 0 -10
f1 (ppm)

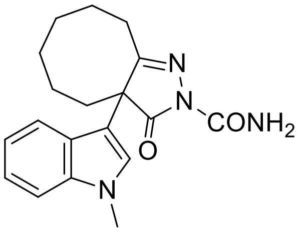
N
N
CONH2
O
N

4o

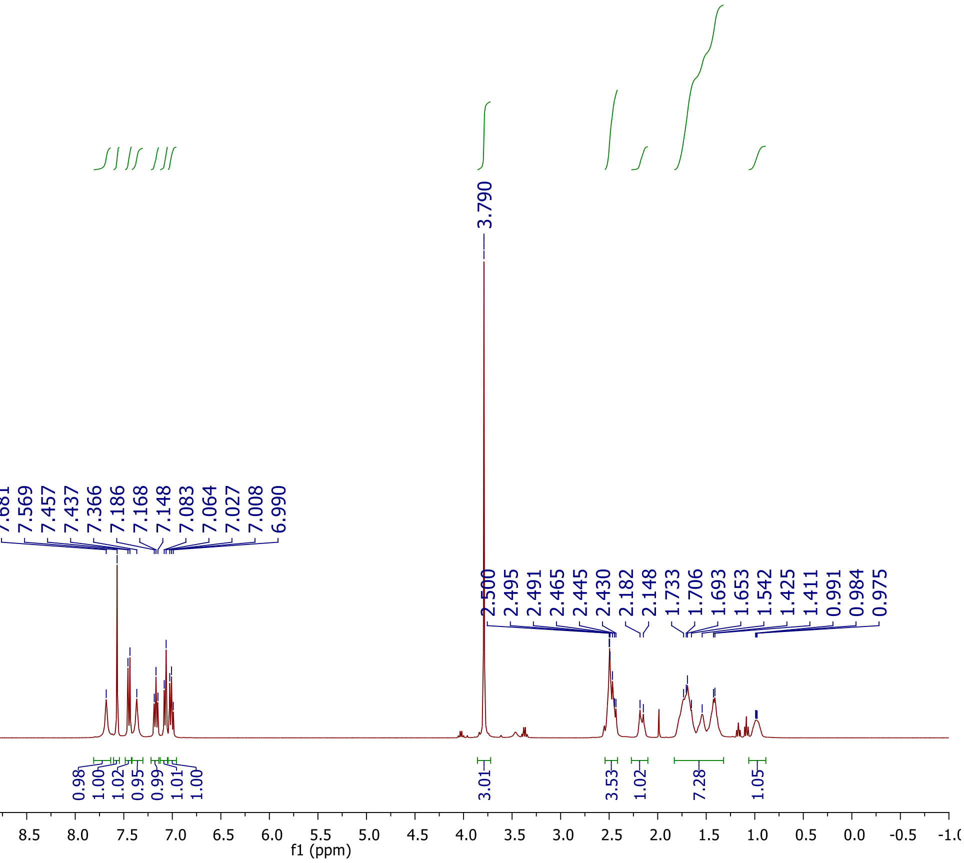
7.681
7.569
7.457
7.437
7.366
7.186
7.168
7.148
7.083
7.064
7.027
7.008
6.990
3.790
2.500
2.495
2.491
2.465
2.445
2.430
2.182
2.148
1.733
1.706
1.693
1.653
1.542
1.425
1.411
0.991
0.984
0.975
0.98
1.00
1.02
0.95
0.99
1.01
1.00
3.01
3.53
1.02
7.28
1.05
f1 (ppm)

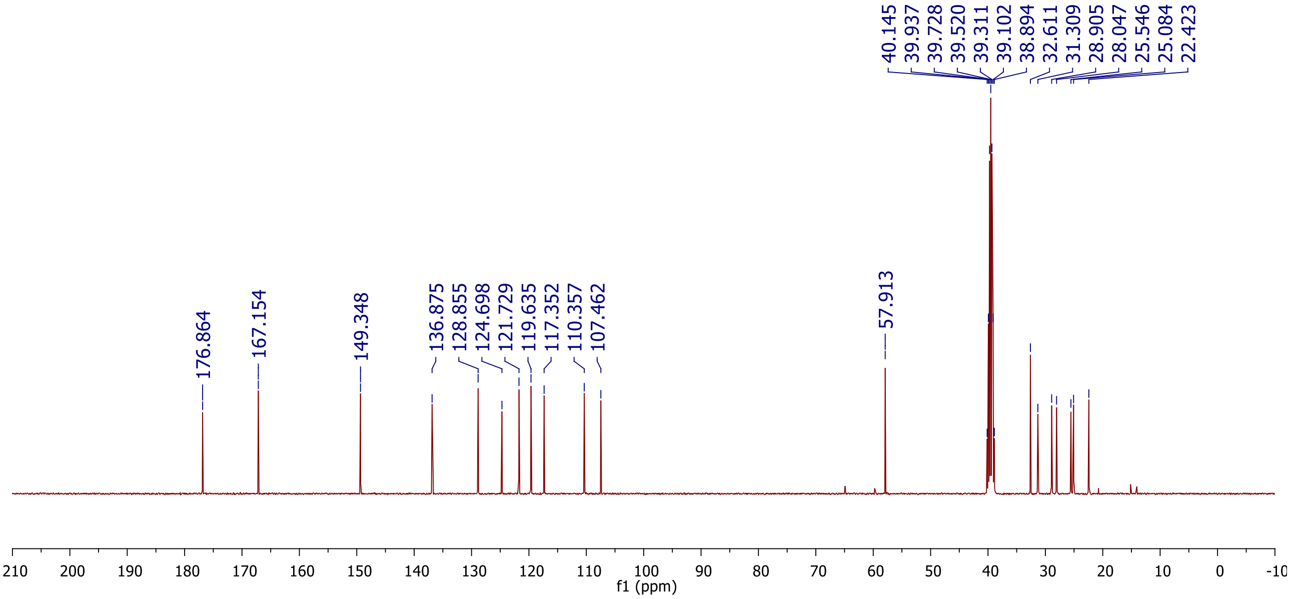
176.864
167.154
149.348
136.875
128.855
124.698
121.729
119.635
117.352
110.357
107.462
57.913
40.145
39.937
39.728
39.520
39.311
39.102
38.894
32.611
31.309
28.905
28.047
25.546
25.084
22.423
f1 (ppm)

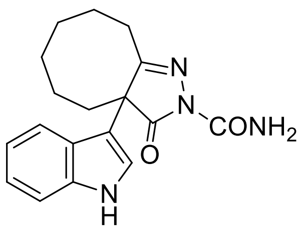
N
N
CONHₓ
O
N
H

4p

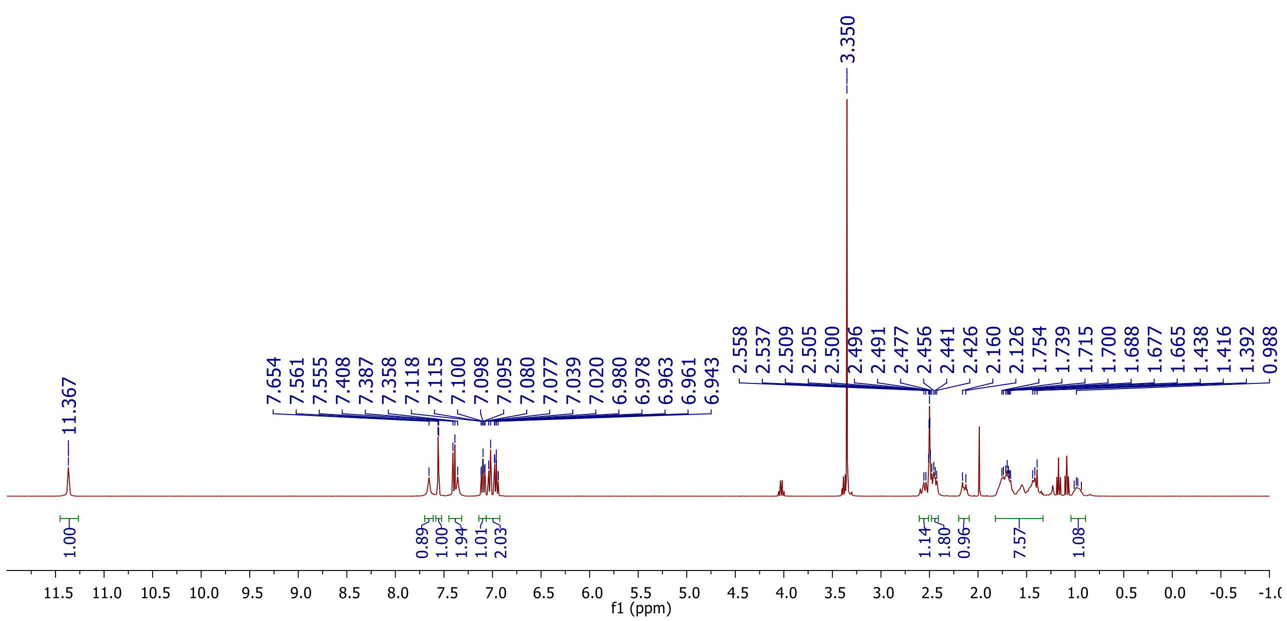
3.350
11.367
7.654
7.561
7.555
7.408
7.387
7.358
7.118
7.115
7.100
7.098
7.095
7.080
7.077
7.039
7.020
6.980
6.978
6.963
6.961
6.943
2.558
2.537
2.509
2.505
2.500
2.496
2.491
2.477
2.456
2.441
2.426
2.160
2.126
1.754
1.739
1.715
1.700
1.688
1.677
1.665
1.438
1.416
1.392
0.988
1.00
0.89
1.00
1.94
1.01
2.03
1.14
1.80
0.96
7.57
1.08
f1 (ppm)

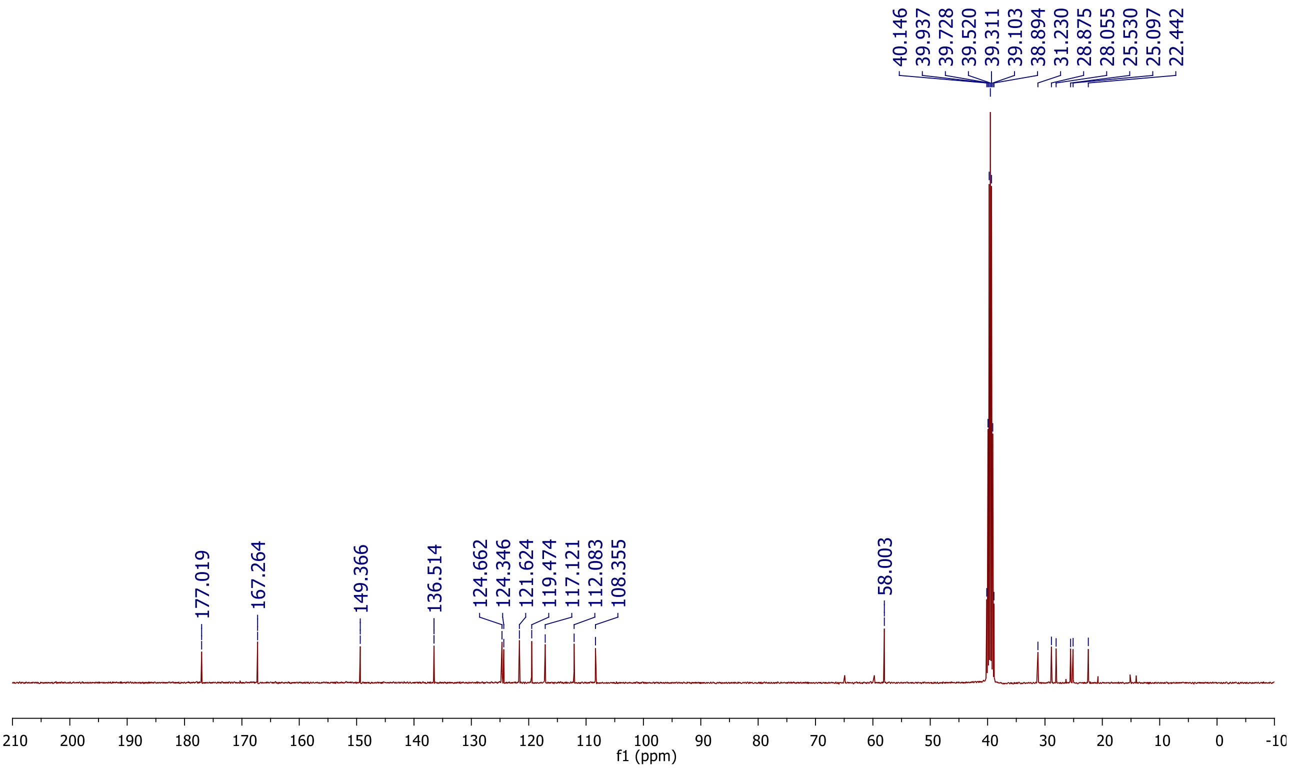
40.146
39.937
39.728
39.520
39.311
39.103
38.894
31.230
28.875
28.055
25.530
25.097
22.442
177.019
167.264
149.366
136.514
124.662
124.346
121.624
119.474
117.121
112.083
108.355
58.003
f1 (ppm)
